# Disordered regions in proteusin peptides guide post-translational modification by a flavin-dependent RiPP brominase

**DOI:** 10.1038/s41467-024-45593-5

**Published:** 2024-02-10

**Authors:** Nguyet A. Nguyen, F. N. U. Vidya, Neela H. Yennawar, Hongwei Wu, Andrew C. McShan, Vinayak Agarwal

**Affiliations:** 1 School of Chemistry and Biochemistry, Georgia Institute of Technology, Atlanta, GA 30332 USA; 2 The Huck Institutes of the Life Sciences, Pennsylvania State University, University Park, PA 16802 USA; 3 School of Biological Sciences, Georgia Institute of Technology, Atlanta, GA 30332 USA

**Keywords:** Biosynthesis, Peptides, Solution-state NMR, SAXS, Computational biophysics

## Abstract

To biosynthesize ribosomally synthesized and post-translationally modified peptides (RiPPs), enzymes recognize and bind to the N-terminal leader region of substrate peptides which enables catalytic modification of the C-terminal core. Our current understanding of RiPP leaders is that they are short and largely unstructured. Proteusins are RiPP precursor peptides that defy this characterization as they possess unusually long leaders. Proteusin peptides have not been structurally characterized, and we possess scant understanding of how these atypical leaders engage with modifying enzymes. Here, we determine the structure of a proteusin peptide which shows that unlike other RiPP leaders, proteusin leaders are preorganized into a rigidly structured region and a smaller intrinsically disordered region. With residue level resolution gained from NMR titration experiments, the intermolecular peptide-protein interactions between proteusin leaders and a flavin-dependent brominase are mapped onto the disordered region, leaving the rigidly structured region of the proteusin leader to be functionally dispensable. Spectroscopic observations are biochemically validated to identify a binding motif in proteusin peptides that is conserved among other RiPP leaders as well. This study provides a structural characterization of the proteusin peptides and extends the paradigm of RiPP modification enzymes using not only unstructured peptides, but also structured proteins as substrates.

## Abstract (web summary)

Here the authors use NMR, SAXS and MD simulations to characterise the structure of proteusin peptides, which are atypically long RiPP substrates. They show a small, unstructured region in the proteusin leader is sufficient for its interaction with a halogenase that brominates the terminal tryptophan residue.

## Introduction

The biosynthesis of RiPPs is predicated upon the recognition of a precursor peptide by peptide modifying enzymes. Typically, the RiPP precursor peptides are divided among an N-terminal leader region, which serves as the recognition element for the modifying enzymes, and the C-terminal core region that is post-translationally modified. Subsequent proteolytic removal of the leader furnishes the mature RiPP^1^.

**Fig. 1:**
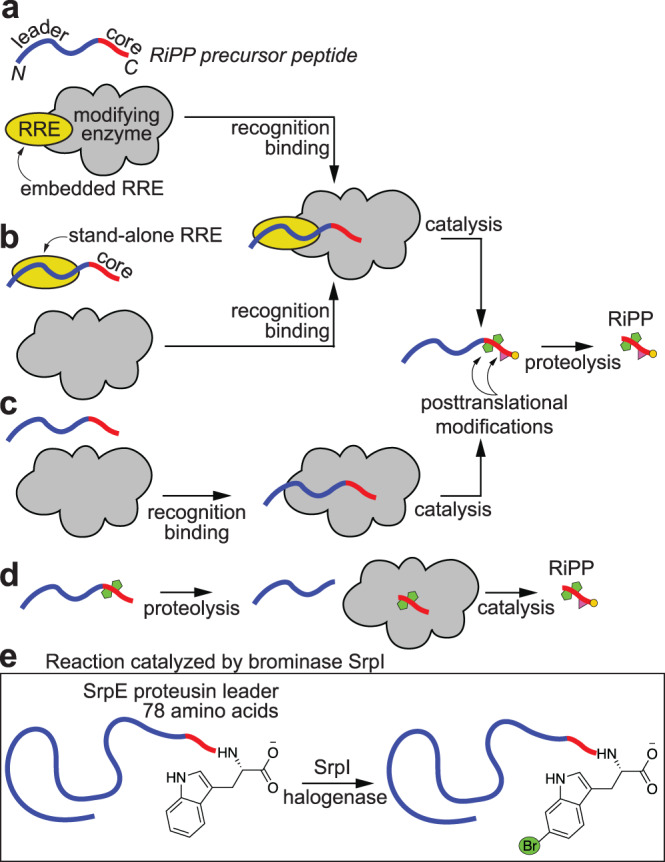
Models for leader peptide engagement. RiPP leader peptide engagement with peptide modifying enzymes can be mediated by (**a**) fused RRE domains, or (**b**) by *trans*-acting stand-alone RRE domains. **c** Peptide-enzyme interaction may involve no RRE domains entirely. **d** Model for leader-independent tailoring. Tailoring of a partially modified core can proceed without the leader peptide being present. **e** Reaction catalyzed by the SrpI brominase. The SrpI brominase requires the presence of the unusually long proteusin leader for brominating the C-terminal Trp side chain of the proteusin SrpE core.

The intermolecular peptide-protein interactions between the RiPP precursor and the modifying enzymes can be mediated by domains called the RiPP precursor peptide recognition element (RRE, Fig. 1)^2^. RRE domains can either be embedded within the peptide modifying enzymes, or they can be stand-alone proteins wherein they bind the leader and deliver the precursor peptide in trans to the modifying enzymes (Fig. 1a, b, respectively)^3–8^. The peptide-protein interaction can be RRE independent, in that, no leader-binding RRE domain is involved (Fig. 1c)^9^. Finally, interaction with and modification of the RiPP precursor peptide core can be independent of the leader peptide itself, in that, tailoring of the core proceeds without the leader peptide being present (Fig. 1d). Within a RiPP biosynthetic scheme, different enzymes can adopt different peptide binding models.

We have recently described the discovery and activity of a leader-dependent RiPP halogenase, SrpI, that brominated the side chain indole of the C-terminal Trp residue of proteusin peptides (Fig. 1e)^10,11^. Together with nitrogen fixation protein 11-like peptides (Nif11 peptides), proteusins are a class of RiPP precursor peptides that are classified by the presence of atypically long leader sequences, often 80–90 amino acids or longer, as compared to that of lasso- and lanthipeptides that are typically 40 amino acids in length (lasso- and lanthipeptides are illustrative examples from among numerous other RiPP families)^1^. Proteusin peptides and their long leader sequences were initially detected bioinformatically, followed by Piel’s discovery and characterization of the polytheonamide biosynthetic gene cluster (BGC), which provided experimentally validated BGC→natural product correspondence for proteusin peptides^12,13^. Proteusin peptides are widely encoded in bacterial genomes and ubiquitously detected in metagenomic datasets. Since the characterization of the polytheonamide BGC, other BGCs with proteusin peptides encoded within and the corresponding RiPPs have been described by Piel and others, including the marine sponge derived RiPP/proteusin BGCs (*srp* BGCs) from which SrpI is derived (Supplementary Fig. 1)^10,14–17^.

The activity of the leader-dependent proteusin brominase SrpI is in contrast to that of the leader-independent lanthipeptide chlorinase MibH (Supplementary Fig. 2). While SrpI requires the presence of the leader in the proteusin precursor peptide SrpE (Fig. 1e)^11^, MibH chlorinated a Trp side chain on the precursor peptide MibA only when the leader had been proteolytically removed from the modified MibA core (model illustrated in Fig. 1d)^18^. Furthermore, SrpI does not possess an embedded RRE domain, and no trans-acting RRE was needed for the SrpI-mediated bromination of the SrpE core^11^. Progressing from these observations, while it is apparent that SrpI follows the model illustrated in Fig. 1c for engaging with the SrpE proteusin precursor, details concerning the intermolecular peptide-protein interactions have remained elusive. The atypical length of the proteusin precursor peptide, which in itself is bereft of any structural characterization, further complicates deciphering the rules governing SrpI substrate engagement. Bromination of Trp side chains in peptides and proteins enables their chemoenzymatic diversification via bioorthogonal transition metal-assisted catalysis^11,19–22^. Lack of description of the molecular determinants for proteusin peptide/SrpI engagement inhibits the further development of SrpI as a biocatalyst. Furthermore, the long proteusin leader in itself compromises the atom economy of producing a derivatized core peptide; any reduction in the leader length that would still allow for efficient SrpI-mediated bromination to proceed would thus be desirable from a biocatalysis point-of-view.

In this study, using solution state nuclear magnetic resonance (NMR) spectroscopy and small-angle X-ray scattering (SAXS), we determine the three-dimensional structure of a proteusin precursor peptide. With support from molecular dynamics (MD) simulations, we show that unlike our current understanding of canonical RiPP leaders, the much longer proteusin leader is divided into a rigidly ordered N-terminal helical bundle, followed by a largely unstructured C-terminal region that is followed by the RiPP core. Using NMR titration spectroscopy, we map the intermolecular peptide-protein interaction interface between the proteusin substrate and the SrpI brominase to the loosely structured C-terminal region of the proteusin leader. We truncate the proteusin leader and demonstrate that despite not possessing an RRE domain, the SrpI halogenase interacts with a highly conserved motif in the SrpE leader that has been previously shown to modulate interactions with the RRE domain in other RiPP tailoring enzymes^4^.

## Results and Discussion

### Design of a chimeric proteusin substrate

**Fig. 2:**
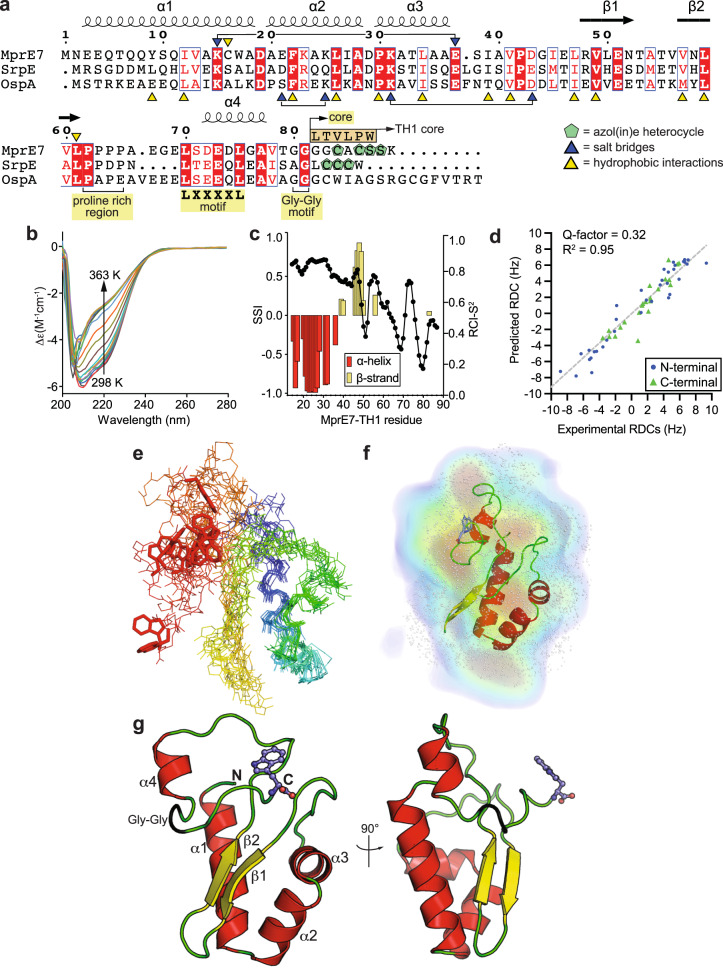
Proteusin peptide structure. **a** Sequence alignment between proteusin peptides MprE7, SrpE, and OspA. For this study, the MprE7 core was replaced with TH1 hexapeptide. The Cys/Ser residues in the cognate MprE7 and SrpE cores are converted to azol(in)es; no heterocycles are installed in OspA. Residues involved in constructing the hydrophobic nucleus in the MprE7 leader and those involved in salt bridge interactions are marked with yellow and blue triangles, respectively (*vide infra*). **b** CD spectra for MprE7 leader measured across 298–363 K in 5 K increments. **c** TALOS-N predicted SSI (red and yellow histogram) and RCI-S^2^ (black scatter) derived from assigned backbone chemical shifts of “state A” of MprE7-TH1. **d** Correlation between experimental ^1^D_NH_ RDCs for MprE7-TH1 measured in *Pf1* phage and Bax DC server^69^ predicted ^1^D_NH_ RDCs values for the lowest energy NMR structure of MprE7-TH1. RDCs corresponding to the rigid N-terminal domain (residues 1–59) and the disordered C-terminal domain (residues 60–87) are shown as blue spheres and green triangles, respectively. The Q-factor and linear regression R^2^ values for the best fit of all data are noted. **e** Overlay of the ten lowest energy NMR structures of MprE7-TH1 guided by chemical shift, NOE, and RDC data of “state A” NMR peaks. The peptide main chain is shown as lines with colors ranging from blue at the N-terminus to red at the C-terminus. Side chain of the terminal Trp residue that is brominated by SrpI is shown in stick representation. **f** The density from solution scattering (DENSS)^36^ electron density envelope, represented as a transparent surface, generated from the SAXS dataset for MprE7-TH1 at a concentration of 523 μM. The top-ten NMR-derived models were assessed using the ATSAS CRYSOL^70^ and OLIGOMER^71^ programs and further refined through explicit-solvent all-atom molecular dynamics simulations with the WAXSiS server; the best model was manually superimposed onto the electron density envelope using PyMOL. **g** Two views of the lowest energy NMR structure of MprE7-TH1 with secondary structure elements denoted as per panel A.

The physiological substrate for the brominase SrpI is the proteusin peptide SrpE wherein the three contiguous Cys residues in the SrpE core (LCCCW) have been cyclodehydrated to thiazolines by the YcaO cyclodehydratase SrpC (Fig. 2a)^10,11,23^. Recombinant expression and purification of SrpE was challenging, as was the enzymatic post-translational cyclodehydration of the three Cys residues in the SrpE core^11^. Thus, we sought alternate substrates for SrpI. Gratifyingly, leader sequences from other proteusin peptides, MprE7 and OspA, supported SrpI activity^10,15,17^. Of these, a gene encoding the MprE7 leader sequence offered robust expression in *Escherichia coli* (Supplementary Figs. 3, 4, Supplementary Table 1).

Circular dichroism (CD) spectroscopy spectra for recombinant MprE7 leader demonstrated characteristic bands at 208 and 222 nm, which indicated the presence of stable α-helical secondary structural elements (Fig. 2b). Deconvolution of the CD spectra with the BeStSel v2023 server suggested a mix of α-helical, β-sheet, and disordered secondary structure elements (Supplementary Fig. 5)^24^. A moderate thermal denaturation temperature (T_m_) of 50 ± 0.5 °C was indicative of a stable tertiary structure (Supplementary Fig. 6). These findings were in contrast to other, significantly shorter RiPP leader peptides that form random coils or singular transiently stable amphipathic helices^25–29^. Driven by these spectroscopic hints at the possible structural features of proteusin leaders, we sought a comprehensive structure determination of MprE7 as a model to understand proteusin peptide structure and function. To eliminate the complication of post-translational installation of azol(in)e rings in the core region of SrpI substrates, we appended the tumor homing peptide-1 (TH1) hexapeptide core to the MprE7 leader to generate the MprE7-TH1 chimeric proteusin peptide (Fig. 2a; TH1: LTVLPW). We have previously demonstrated that the terminal Trp residue in the TH1 core, which possesses no azol(in)e heterocycles, is efficiently brominated by SrpI (Supplementary Fig. 4, Supplementary Table 2)^11^.

### Solution structure of a proteusin precursor

SAXS indicated globular monomers for MprE7-TH1 in solution with radius of gyration of approximately 18.4 Å which supported our abovementioned hypothesis that proteusin peptides possess tertiary structure (Supplementary Figs. 7–9, Supplementary Table 3). The Kratky plots at three different protein concentrations exhibited semi-gaussian profiles which was suggestive of flexibility in MprE7-TH1 conformation (Supplementary Fig. 10). Structure determination for the chimeric MprE7-TH1 proteusin peptide was pursued using solution NMR spectroscopy. Uniformly ^15^N and ^13^C isotopically labeled (U-[^15^N/^13^C]) MprE7-TH1 proteins were produced (Supplementary Table 4), and we assigned 72.5% of chemical shifts corresponding to backbone H^N^, N^H^, Cα, Cβ, and C atoms using sequential assignment techniques based on three dimensional triple-resonance NMR experiments (Supplementary Figs. 11–14, Supplementary Table 5)^30^. As we discuss below (*vide infra*), in the 2D ^1^H-^15^N HSQC of unbound MprE7-TH1, two sets of amide cross peaks corresponding to residues spanning C-terminal domain were observed at different populations (residues 66–87, Supplementary Figs. 11–13, 20); for structural assignment, we used the most intense (~80%) cross peaks for each residue. Next, using TALOS-N^31^, we calculated the secondary structure index (SSI) for the assigned “state A” NMR peaks, which indicated the presence of α-helices at the N-terminus of MprE7-TH1, followed by a short span of residues comprising of β-sheets (Fig. 2c). NMR derived SSI values agree with CD spectroscopy and SAXS observations that MprE7 is a mixture of α-helical, random coil, and β-sheet elements organized in a tertiary structure. Note that all spectroscopy experiments were performed in aqueous solvents that contain no secondary structure stabilizing organic co-solvents^32,33^. The estimated random coil index order parameter (RCI-S^2^, 0 and 1 signify disorder and rigid structure, respectively)^34^ provided further support to the proteusin MprE7-TH1 peptide N-terminus being highly ordered followed by increasing disorder towards the C-terminus (Fig. 2c). Next, we combined the chemical shifts, dihedral angle restraints from TALOS-N, amide to amide NOE-based distance constraints, and amide residual dipolar couplings (RDCs) to generate a set of energy minimized solution structure models for the MprE7-TH1 proteusin peptide^35^. Based on these models, an excellent agreement between the experimental and theoretically predicted RDCs was observed (Fig. 2d, e). Furthermore, the overlay of the NMR/WAXSiS MprE7-TH1 model into the volume data of the SAXS-derived solvent envelopes, calculated in Chimera, demonstrated a good correlation of 0.9 (Fig. 2f, Supplementary Fig. 15)^36–38^. This agreement between the two analyses provides additional support for the accuracy and consistency of the obtained room-temperature solution structural model of MprE7-TH1.

The N-terminus of the MprE7-TH1 peptide is folded into a tri-helical bundle (α1–α3, Fig. 2g), followed by two antiparallel β sheets (β1, β2). The α1–α3 and β1, β2 bury a hydrophobic core (in gray, Supplementary Figs. 16, 17). The residues, the side chains of which construct this hydrophobic core, are largely conserved in the proteusin leader sequences that support SrpI activity (yellow triangles, Fig. 2a). The hydrophobic nucleus of the proteusin leader is reminiscent of acyl carrier proteins in which α-helices similarly bury a hydrophobic cavity^39^. Three salt bridges, formed between the side chains of Glu21 and Lys25, Glu37 and Lys15, and Asp43 and Lys31 rigidify the tertiary structure (Supplementary Fig. 16). Of these, the Glu37/Lys15 and Asp43/Lys31 pairs are conserved in the proteusin leader sequences (blue triangles, Fig. 2a). Progressing from β2, the remainder of the proteusin leader peptide is largely disordered, apart from a short α4 helix. A proline rich region separates β2 and α4 (Fig. 2a). The presence of the α4 helix is supported by experimental NOE patterns consistent with an alpha helical structure spanning residues 73–77 (Supplementary Fig. 18). The α4 helix, which spans the conserved LXXXXL sequence motif, makes minimal contacts with the tertiary structure at the N-terminus of the proteusin leader. Four amino acids separate the LXXXXL motif from the double glycine motif that marks the leader/core boundary; these residues likewise make no contact with the rest of the N-terminal tertiary structure. Counting backwards from the leader/core boundary, the LXXXXL motif occupies residues -12 through -7 (Fig. 2a).

In 2010, Mitchell and co-workers bioinformatically detected the presence of the atypically long proteusin substrate peptides in sequence databases and identified their similarity to the alpha subunit of nitrile hydratases, leading to their prevailing annotation as nitrile hydratase-like proteins (NHLPs)^12^. The structural similarity between the N-terminal structured region of the MprE7 proteusin leader and the *Bacillus smithii* nitrile hydratase is immediately apparent, with the α1–α3 and β1 secondary structural elements in good alignment (Supplementary Fig. 17)^40,41^. Progressing further from β1, the nitrile hydratase possesses additional structural elements that bind a cobalt cation; these secondary structural elements are absent in proteusin peptides. Downstream of β2 and the proline rich region, there is little to no structural correspondence between the proteusin precursor and the nitrile hydratase alpha subunit. The α1–α3 helical bundle also possesses similarity to the C-terminal domains of self-methylating fungal peptides that furnish borosin RiPPs^42–44^.

### Molecular dynamics simulation of the proteusin peptide structure

Taken together, a structural model for proteusin peptides emerges. The proteusin leader is divided into two sections; the N-terminal 60 residues are rigidly structured, and the tertiary structure is organized around a hydrophobic core and stabilized by multiple salt bridges. Separated by a proline rich region, the remaining leader peptide residues are disordered (Fig. 2a). Relative to the N-terminal half, the second half of the proteusin leader and the proteusin core are flexible; this model is supported by an alignment of the ten most energy minimized structures of MprE7-TH1 (Fig. 2e).

**Fig. 3:**
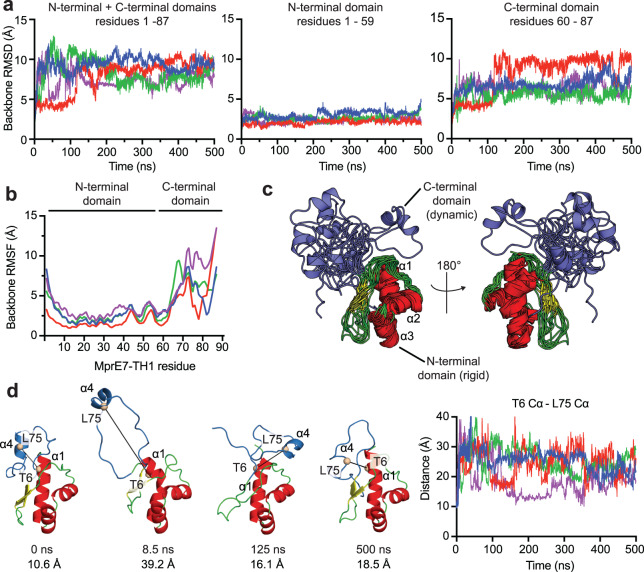
Conformational flexibility in proteusin peptides. **a** Plot of backbone RMSD versus time for MprE7-TH1 calculated relative to the input structure along four independent 500 ns trajectories. For comparison, RMSD calculations were performed separately for Left: combined N-terminal and C-terminal domains (residues 1–87), Middle: the N-terminal domain (residues 1–59), and Right: the C-terminal domain (residues 60–87). **b** Plot of backbone RMSF versus MprE7-TH1 residue number along four independent 500 ns trajectories. Both (panels a and b) backbone calculations include N, Cα, and C atoms. **c** Representative structures of MprE7-TH1 obtained from GROMACS cluster analysis. N-terminal domain residues comprising of α1–α3 helices and β1 and β2 strands are colored according to secondary structure elements. The remaining C-terminal residues are colored blue, which comprise of the proline rich region, the a4 helix, and the TH1 core. **d** Left: Representative structural snapshots of MprE7-TH1 from MD simulations at specific timepoints with distance measurements between the T6 Cα atom (located on α1 helix) and L75 Cα atom (located on α4 helix). Right: Distance measurements between T6 Cα and L75 Cα atoms along four independent 500 ns trajectories. For (**a**, **b**, **d**) plots of MD data from the four replicates are shown in different colors for ease of visualization. For (**c**, **d**), MprE7-TH1 structures are shown with the N-terminal domain colored red (helix)/green(random coil)/yellow(β-sheet) and the C-terminal domain colored blue.

To explore the validity of the abovementioned model, we queried the backbone dynamics of MprE7-TH1 using all-atom MD simulations performed in explicit solvent in four independent 500 nanosecond trajectories. Mapping the backbone root mean square deviation (RMSD) and backbone root mean square fluctuation (RMSF) demonstrated rigidness of the structured N-terminal domain and large conformational flexibility of the C-terminal half of the leader peptide following the proline rich region (Fig. 3a, b, Supplementary Fig. 18). A GROMACS cluster analysis corroborated these findings, indicating large-scale movement of α4, while α1–α3 and β1, β2 remained rigidly in place (Fig. 3c)^45^. Mapping the α4 dynamics and its contribution to the overall RMSDs was enabled by monitoring the time-dependent change in the distance between the Cα atoms of Thr6 (residue on α1) and Leu75 (α4 residue, Leu75 is the terminal residue of the LXXXXL motif) (Fig. 3d). Two lines of evidence suggest that the α4 helix is likely only transiently populated: i) the α4 helix undergoes transitions between α-helical and random-coil like secondary structures during MD simulations (Fig. 3c, d; Supplementary Fig. 18), and ii) chemical shift derived SSI predictions did not reveal strong α-helical signal for residues 70–80 (Fig. 2c). However, the α4 helix is supported by a moderate to high degree of stability in MD simulations, increased RCI-S^2^ values, and NOE patterns consistent with α-helical structures (Fig. 2c, Supplementary Fig. 18).

### Interaction of a proteusin precursor with halogenase SrpI

**Fig. 4:**
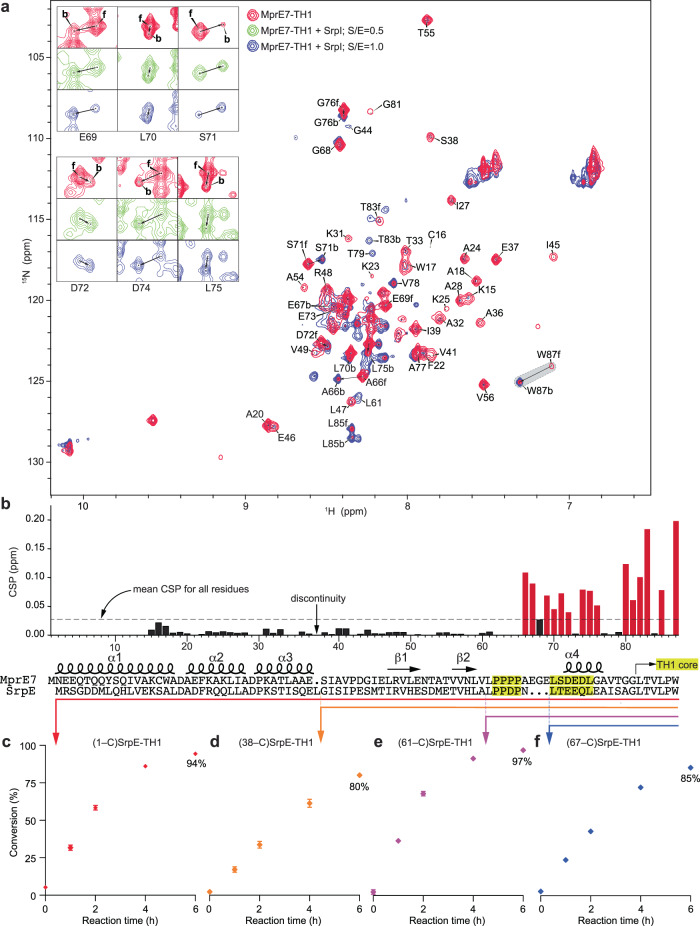
SrpI binds to disordered regions in proteusin peptides. **a** Overlaid ^1^H-^15^N HSQC spectra for MprE7-TH1 (in red), and MprE7-TH1 titrated with equimolar ratio of SrpI (in blue). Cross peaks corresponding to select MprE7-TH1 residues are labeled. For residues that demonstrate shifts in cross peaks upon titration with SrpI, ‘f’ and ‘b’ refer to cross peaks observed for ‘free’ MprE7-TH1 and for MprE7-TH1 ‘bound’ to equimolar SrpI, respectively. Cross peaks corresponding to the C-terminal Trp residue that is brominated by SrpI are highlighted in grey. Inset shows cross migration for six select MprE7-TH1 residues at two different MprE7-TH1:SrpI ratios. **b** Calculated CSP values for MprE7-TH1 residues upon titration with equimolar SrpI. CSP was calculated for state “A” peaks in free MrpE7-TH1 relative to state “B” peaks in the complex with SrpI. The dashed line represents the mean CSP for all MprE7-TH1 residues. CSPs greater than the mean are highlighted in red. Note that the alignment between the MprE7-TH1 and SrpE-TH1 chimeric sequences necessitated the manual introduction of a single residue discontinuity in the CSP histogram. CSP values cannot be determined for Pro residues as they possess no cross peaks in the ^1^H-^15^N HSQC spectra. The proline-rich region and the LXXXXL motif are highlighted in yellow. Time-dependent evaluation of SrpI activity for (**c**) full length SrpE-TH1 proteusin substrate, and (**d**–**f**) SrpE leader truncation mutants. Reaction aliquots were quenched at designated time points, and product formation was evaluated using liquid chromatography/mass spectrometry (LC/MS) by detection of the GluC-digested peptide fragments. Area under the extracted ion chromatograms (EICs) corresponding to the substrate and product peptide fragments was used to determine the conversion. Data points and error bars denote the mean and the standard deviation from three independent experiments, respectively. Source data are provided as a Source Data file.

Given the bipartite structure of the atypically long proteusin leader, we were curious to know which regions of the MprE7 leader contributed to binding to the SrpI brominase. While MprE7 is not the cognate substrate for SrpI, the MprE7 leader supports SrpI activity (Supplementary Fig. 4)^11^. We titrated unlabeled SrpI into ^15^N-labeled MprE7-TH1 in the presence of the flavin adenine dinucleotide (FAD) co-factor and monitored chemical shift changes in the 2D ^1^H-^15^N HSQC spectra (Fig. 4a, Supplementary Table 6). NMR cross peaks corresponding to the MprE7 leader exhibited NMR line broadening upon addition of SrpI (SrpI-dependent decrease in peak intensity), which demonstrated a specific interaction between SrpI and MprE7. The observed NMR line broadening of MprE7-TH1 amide N-H signals could be due to rapid transverse spin relaxation in the ~60 kDa MprE7-TH1/SrpI complex, or other changes in the exchange behavior of MprE7-TH1 in the SrpI complex. A peak intensity ratio analysis of “state A” peaks obtained at the 1:1 MprE7-TH1:SrpI titration stoichiometry did not identify a specific site of MprE7 sequence experiencing lower than average line broadening (Supplementary Fig. 19). We also observed slow exchange NMR chemical shift behavior for the C-terminal residues of MprE7-TH1 upon addition of SrpI (SrpI-dependent peak shifts between free and bound states), which hinted that the C-terminal half of the MprE7 undergoes a conformational change upon SrpI interactions, possibly due to serving as a major site for interaction with SrpI. This was supported by a peak intensity analysis of “state B” NMR peaks for MrpE7-TH1 in the absence and presence of SrpI (Supplementary Figs. 19 and 20). The overall all intensity of “state A” peaks relative to “state B” peaks decrease in the presence of SrpI, suggesting SrpI binding drives a transition between the two states of MprE7-TH1 (Supplementary Figs. 19 and 20).

Without definitively precluding either the conformational selection or the induced fit models for substrate–enzyme binding, it could be inferred that interaction with SrpI biases the conformation of the C-terminal region of the proteusin leader along with the core peptide for productive peptide-enzyme complex formation and for catalysis to proceed (Fig. 4a-inset, Supplementary Fig. 20)^46^. This assertion was supported by the residue-specific chemical shift perturbation (CSP) analyses for MprE7-TH1 upon titration with SrpI. Here, MprE7 residues following the proline rich region, and the TH1 core demonstrated higher than mean CSP scores (Fig. 4b). Importantly, the “state B” NMR peaks that exhibit CSPs also undergo an increase in peak intensity upon complex formation with SrpI (Supplementary Figs. 19 and 20). These residues (amino acids 66 – 82) include the α4 helix, which spans the LXXXXL motif. Taken together, these data suggest that the intermolecular peptide-protein interactions between MprE7-TH1 and SrpI are driven by the loosely structured C-terminal half of the MprE7 leader. Note that Pro residues, such as the four contiguous Pro residues in the MprE7-TH1 proline rich region (highlighted in Fig. 4b), do not possess cross peaks in a ^1^H-^15^N HSQC spectra. Global NMR lineshape analysis performed in TITAN v1.6-12-g9041 for eight residues in the MprE7 leader demonstrating CSP greater than the mean determined interaction parameters for the MprE7-TH1/SrpI complex: apparent K_D_ of 24.9 ± 0.6 µM and k_off_ of 3.7 ± 0.5 s^−^^1^ (Supplementary Figs. 21–27, Supplementary Table 7)^47^; the equilibrium dissociation constant K_D_ is in good agreement with other RiPP leaders binding to their corresponding modifying enzymes^4,7,27^.

### Truncated proteusin leaders support SrpI activity

Next, we sought to biochemically corroborate the spectroscopic observation that the loosely structured C-terminal half of the proteusin leader, and not the rigidly structured N-terminal half, was the primary determinant for binding to SrpI. Here, we mapped the structural regions of the MprE7-TH1 proteusin peptide to the chimeric SrpE-TH1 substrate (Fig. 4b); SrpE leader is the physiological interaction partner for SrpI. A series of truncation mutants were created, and ability of SrpI to brominate the C-terminal Trp side chain for these truncation mutants were evaluated relative to the full length SrpE-TH1.

SrpI is expected to follow the well-accepted mechanistic model for flavin-dependent halogenases wherein a flavin-reductase partner enzyme provides the reduced flavin cofactor, FADH_2_, to SrpI^48,49^. The reduced FADH_2_ cofactor facilitates formation for the peroxyflavin intermediate which is resolved by halide oxidation followed by halonium/hypohalous acid stabilization by an active site lysine residue^50,51^. We have previously demonstrated that the mutation of this lysine residue, Lys84 for SrpI, to an alanine abolished SrpI activity^10^. In line with this mechanistic framework, we employed the flavin reductase RebF for in vitro activity reconstitution of SrpI together with phosphite dehydrogenase PTDH for in situ production of NADH, the electron source for flavin reduction by RebF^52,53^.

SrpI demonstrated a near stoichiometric time-dependent bromination of the full length SrpE-TH1 (residues 1–C-term, Fig. 4c). The SrpE α1−α3 secondary structure elements could be eliminated, with only a modest decrease in the bromination efficiency of the truncated substrate (residues 38–C-term, Fig. 4d). Interestingly, while preserving the proline rich region, the entire N-terminal structured half of the proteusin leader could be eliminated (residues 61–C-term, Fig. 4e); these data are in agreement with and support the spectroscopic observations described above. When the proline rich region is also eliminated, the truncated SrpE-TH1 substrate can still be brominated by SrpI, albeit with a slight loss in efficiency (residues 67–C-term, Fig. 4f). Note that while we could not determine CSP values for the proline rich region of the MprE7 leader, the biochemical data demonstrate that this region could potentially be important in interacting with SrpI, either through direct interactions with SrpI or by modulating the conformation of the C-terminal core.

In contrast to the region of MprE7 and SrpE proteusin leaders that we have mapped for interaction with brominase SrpI, scanning mutagenesis of the OspA proteusin leader had identified residues that are placed on the α1 and α2 helices as important for interaction with the epimerase OspD (Fig. 2a)^54^. In a follow up study, however, despite the presence of an embedded RRE domain, OspD was shown to efficiently process substrates in a leader-independent fashion akin to the model illustrated in Fig. 1d^55^. We speculate that alterations in the rigidly structured N-terminal region could alter the overall structure of the proteusin peptide and thus compromise catalytic transformation efficiency independent of the intermolecular peptide-protein interactions.

### Functional relevance of the LXXXXL motif

The region of the proteusin leaders that interact with SrpI span the LXXXXL motif; the ^1^H-^15^N HSQC titration spectra for MprE7-TH1 demonstrate large perturbations for all these residues upon titration with SrpI (Fig. 4a, b). The SrpE-TH1 truncation constructs, which dispense with nearly all of the proteusin leader peptide but retain this LXXXXL motif, are competent substrates for SrpI (Fig. 4d–f). In light of these observations, we sought to generate a residue-level resolved understanding of the contribution of the LXXXXL motif for proteusin peptide binding to SrpI. Specifically, we focused on the SrpE-TH1 -NLTEEQL- sequence which spans residues 66–72 and includes the LXXXXL motif as the corresponding residues in MprE7-TH1 demonstrated higher than mean CSP values upon SrpI titration (Fig. 4b).

**Fig. 5:**
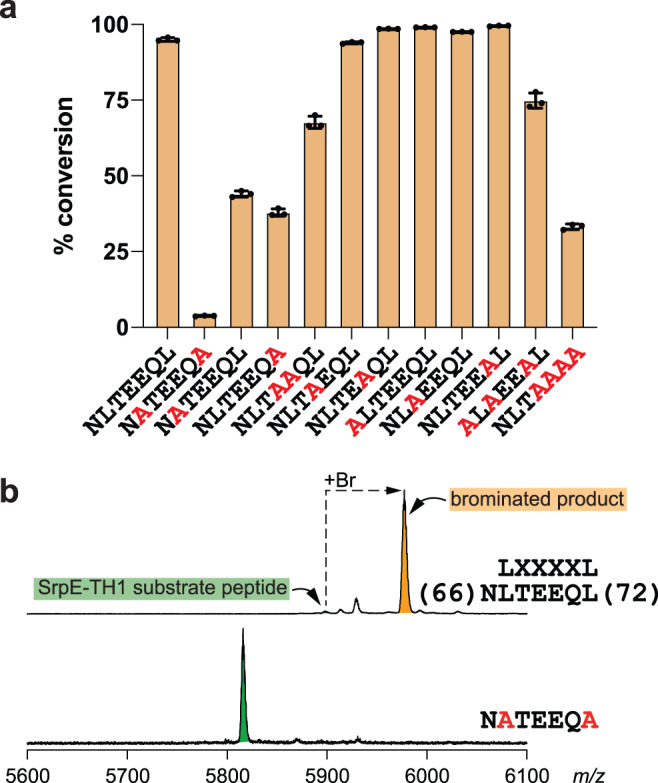
Importance of the LXXXXL motif in SrpI engagement. **a** Comparative extent of bromination of wild type SrpE-TH1 (-NLTEEQL-) and mutant proteusin substrates by SrpI. Error bars are derived from three independent experiments. Data are presented as mean values ± standard deviation. Source data are provided as a Source Data file. **b** MALDI-ToF MS spectra demonstrating bromination of wild type SrpE-TH1 (top) and the -NATEEQA- mutant (bottom) substrates by SrpI. Peaks corresponding to the substrate peptide are shaded green, and product peaks are shaded orange. The sites for mutagenesis are highlighted in red.

As monitored by matrix-assisted laser desorption/ionization time of flight mass spectrometry (MALDI-ToF MS), relative to wild type SrpE-TH1, mutating the -NLTEEQL- heptapeptide to contiguous Pro, Ala, or Gly residues led to complete abolishment of in vitro bromination by SrpI (Supplementary Fig. 28). Using LC/MS, as we had done for the SrpE truncation mutants, we determined that the concomitant mutation of the two Leu residues of the NLTEEQL sequence (Leu67 and Leu72) to Ala also led to near complete loss of in vitro bromination of the TH1 core by SrpI (Fig. 5a; NATEEQA, Supplementary Figs. 29, 30). This observation was corroborated using MALDI-ToF MS wherein, as compared to wild type SrpE-TH1, bromination of the NATEEQA substrate variant was not observed (Fig. 5b). Individual mutations, Leu67Ala and Leu72Ala leading to SrpE-TH1 -NATEEQL- and SrpE-TH1 -NLTEEQA- substrate variants, respectively, likewise demonstrated a reduction in bromination efficiency (Fig. 5a). These data suggest that the side chains of both Leu residues of the LXXXXL motif are involved in proteusin leader peptide engagement with SrpI.

By analogy to the MprE7-TH1 structure, the SrpE-TH1 69–72 residues, -EEQL-, should be placed on the α4 helix (Fig. 2a). Mutating these residues to Ala, leading to the SrpE-TH1 -NLTAAAA- variant, also reduced in vitro bromination by SrpI (Fig. 5a). Mutation of the two Glu residues, Glu69 and Glu70 to Ala (leading to the -NLTAAQL- substrate variant), also had a deleterious impact on bromination by SrpI (Fig. 5a). Individual mutations of the two Glu residues to Ala did not impact the bromination efficiency. Mutation of the polar residues, Asn66, Thr68, and Gln71 together to Ala, individually, also did not impact the bromination efficiency by SrpI; however, the concomitant mutation of Asn66, Thr68, and Gln71 to Ala (leading to the -ALAEEAL- substrate variant) had a deleterious impact on in vitro bromination by SrpI. Altogether, these data demonstrate the primacy of the LXXXXL motif and the α4 helix in directing proteusin substrate engagement with SrpI. Among others, the SrpE residues Leu67 and Leu72 (Leu70 and Leu75 for MprE7) are likely to be intimately involved in engagement with SrpI.

### Conservation of the LXXXXL motif in RiPP biosynthesis

**Fig. 6:**
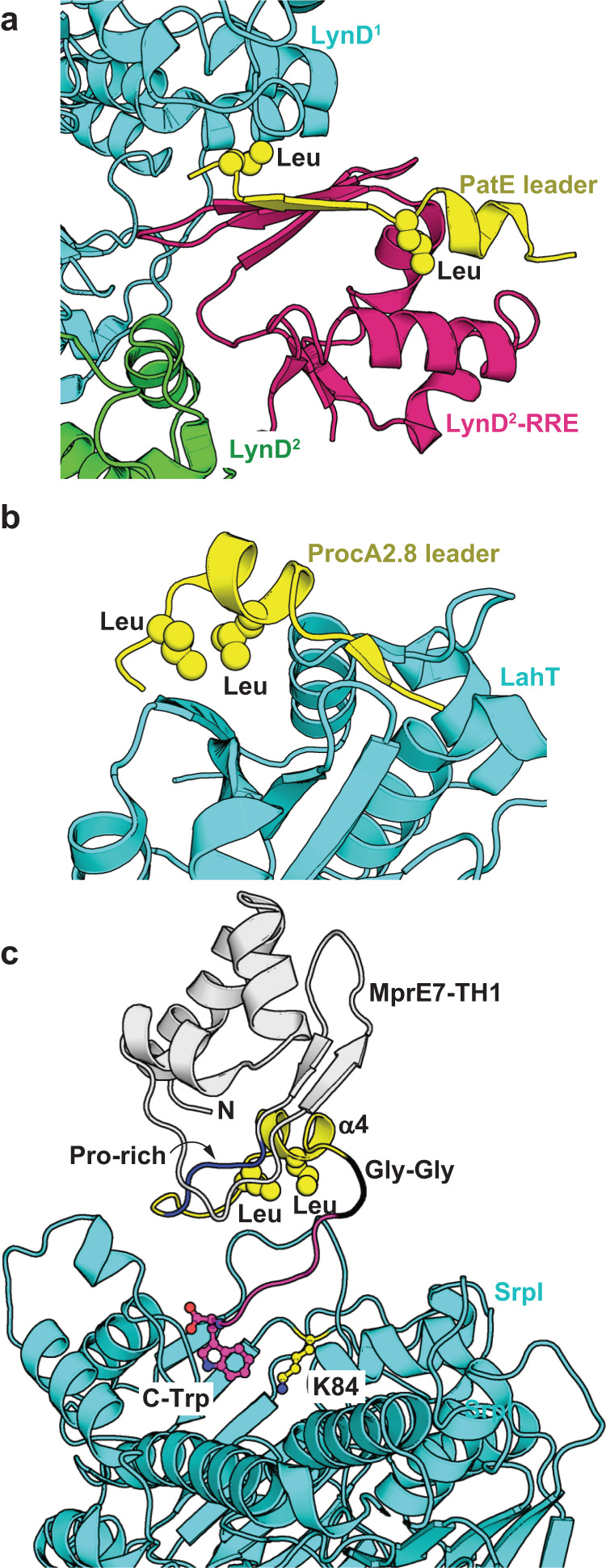
Disparate models for leader peptide binding in RiPP biosynthesis. Crystallographic snapshots of (**a**) RRE-dependent and (**b**) RRE-independent binding of the LXXXXL motif of RiPP leaders to the YcaO cyclodehydratase LynD (PDB: 4V1T) and the protease domain of LahT (PDB: 6MPZ), respectively. For the Leu residues that constitute the LXXXXL motif, the side chain atoms are shown as spheres. In binding to the LynD RRE domain (**a**), the PatE leader domain binds at the interface of two LynD monomers denoted as LynD^1^ and LynD^2^. Here, the LXXXXL motif forms a β-strand to extend the RRE antiparallel β-sheet. For ProcA2.8 leader binding to LahT, the LXXXXL motif is placed on a short α-helix. LahT does not possess an RRE domain. **c** NMR/HADDOCK/MD derived model for MprE7-TH1 binding to SrpI. The N-terminal region of MprE7 is shown in gray cartoon; SrpI is shown in cyan. As before, the side chain atoms for the two Leu residues that comprise the LXXXXL motif in the MprE7 leader are shown as yellow spheres. The MprE7-TH1 C-terminal Trp residue that is positioned close to the SrpI catalytic Lys residue, K84, is shown in stick-ball representation with carbon atoms colored magenta. The proline rich region of the MprE7 leader is shown in blue, and the Gly-Gly motif that marks the leader/core boundary is shown in black.

The LXXXXL motif is not unique to proteusin leaders, it is present in numerous other RiPP precursor peptides. In cyanobactin precursors, this motif lies at the C-terminus of a transiently stable amphipathic helix, and forms an additional antiparallel β-strand with the central β-sheet of the embedded RRE domain within YcaO cyclodehydratases (Fig. 6a, PDB:4V1T)^4,25,56^. While SrpI does not possess an embedded RRE domain and no *trans*-acting RRE domain is encoded in the *srp* BGC or is needed for SrpI activity, the same LXXXXL motif is involved in mediating interactions of proteusin peptides with the SrpI brominase. The RRE-independent interaction of the MprE7 and SrpE LXXXXL motifs with SrpI thus likely mimics the interaction between the Nif11 precursor peptide ProcA2.8 with the LahT protease that cleaves at the leader/core boundary^9^. Here, the LXXXXL motif is present on a short α-helix, akin to the proteusin α4 helix, and interacts with the protease in an RRE-independent fashion (Fig. 6b, PDB:6MPZ)^9^. In both instances, the Leu side chains of the LXXXXL motif insert themselves into hydrophobic pockets at the surface of the corresponding modifying enzymes.

Within the spectroscopically and biochemically defined constraints of the proteusin peptide binding to SrpI, a model for the MprE7-TH1/SrpI complex was developed and refined using docking and MD simulations (Fig. 6c, Supplementary Movie). This hypothetical model, which places the MprE7-TH1 C-terminal Trp residue in the SrpI active site proximal to the catalytic Lys residue^10^ (SrpI Lys84), demonstrates the primacy of the LXXXXL motif-bearing α4 helix of the MprE7 leader in binding to a SrpI surface loop (SrpI residues 104–110). The first 60 residues of the MprE7 leader that comprise the α1–α3 and β1, β2 structural elements make minimal contacts with SrpI, which validate the proteusin leader truncation mutants supporting the bromination activity of SrpI (Fig. 4d–f). The putative role of this SrpI surface loop in mediating interaction with the MprE7 LXXXXL motif was probed with MD simulations of MprE7-TH1 in complex with mutant SrpI (P105G/Q106A/Q107G/V108A/S109G) where distance measurements along the interface revealed destabilization relative to MprE7-TH1 in complex with wild-type SrpI (Supplementary Fig. 31).

Superimposition of the MprE7-TH1/SrpI in silico model with that of the crystal structure of the RiPP chlorinase MibH identifies that the region of SrpI to which MprE7-TH1 binds to is conspicuously different the analogous surface of MibH (Supplementary Fig. 32)^18^. While SrpI requires the presence of the proteusin leader, MibH follows the model illustrated in Fig. 1d wherein halogenation proceeds only after the leader has been removed from the corresponding core peptide. These structural differences perhaps explain the disparate leader requirements of the flavin-dependent halogenases SrpI and MibH.

It is instructive to observe that the same LXXXXL motif is used by different RiPP families (proteusins, cyanobactins, lanthipeptides) in RRE-dependent and RRE-independent manners to mediate intermolecular peptide-protein interactions. The generality of this interaction motif can thus be used to engineer modifications upon RiPP precursors by enabling heterologous precursor peptide-modifying enzyme interactions, as has been demonstrated for cyanobactin precursors and azoline-forming YcaO cyclodehydratases^57^. Steric complementarity, driven primarily by burial of hydrophobic side chains of RiPP leaders into hydrophobic surface patches on RiPP modifying enzymes, is an often repeated peptide-protein interaction model, independent of the LXXXXL motif-mediated binding^7^. Our findings now extend this paradigm to proteusin peptides. It is worth noting that the entire N-terminal structured half of the proteusin peptides is dispensable for binding to SrpI. Curiously, it is easy to imagine that the proline-rich region marks the interface at which a rigidly structured NHLP has been fused to an unstructured canonical RiPP leader-like sequence. RiPP-like posttranslational tailoring is no longer restricted to small peptidic substrates; large proteins and macromolecular assemblies, such as the ribosome, are modified by dedicated tailoring enzymes^58–60^.

Our findings now allow for rationalized separation of these two regions for proteusin peptides and opens the door for testing whether other proteusin peptide modifying enzymes recognize either, or both features of the proteusin leaders. RiPPs derived from proteusin peptide substrates are endowed with unique reactions that are often not represented in other RiPP chemical classes, such as Cα epimerizations and Trp side chain bromination. Understanding the rules governing proteusin peptide engagement with these modification enzymes will facilitate mix-and-match combinatorial biosynthesis with other RiPP chemical classes. Furthermore, as we demonstrated previously, leader-guided enzymatic bromination by SrpI enables chemical diversification of ribosomally-derived peptides and proteins. Improving the atom economy for bromination by a structure-guided leader peptide truncation will aid in future biocatalytic applications of SrpI.

## Methods

### Preparation of constructs for expression of SrpI, PTDH, RebF, and chimeric peptides

Genes optimized for expression in *Escherichia coli* for SrpI, PTDH, RebF, and the proteusin peptides were used as templates for amplifying and subcloning PCR amplicons in plasmid vectors using standard procedures. The chimeric peptides were designed by incorporating the leader peptide SrpE or the leader peptide MprE7 as described in Supplementary Table 1.

PCR reactions (25 µL) contained 20 ng template DNA, 0.4 µM each of reverse and forward primers, 0.2 mM dNTPs, Phusion reaction buffer, and 0.25 U Phusion-high fidelity DNA polymerase (Thermo). PCR amplicons were subcloned into plasmid vectors using Gibson Assembly HiFi master mix (NEB). All constructs were verified by Sanger sequencing.

### Protein expression and purification

For purification of SrpI, PTDH, and RebF, chaperones (from chaperone plasmid pGro7, Takara Biosciences) were co-expressed to assist the folding of SrpI. 1 g/L of L-arabinose was used to induce the expression of chaperones. PTDH and RebF were purified without assistance from chaperones. Here, PTDH is used for in situ regeneration of NADH and RebF is used for in situ reduction of FAD to FADH_2_ which is required for activity of the flavin-dependent halogenase SrpI^48,52,53^. After the addition of inducers (isopropyl-β-d-thiogalactopyranoside (IPTG) and/or L-arabinose) followed by 48 h of incubation at 18 °C, cultures were harvested by centrifugation (7000 × g, 25 min, 4 °C), and cell pellets were resuspended in 50 mL lysis buffer B (20 mM Na-phosphate (pH 7.5), 100 mM NaCl). Cells were lysed by sonication for 30 min at 40% amplitude. The lysates were clarified by centrifugation at 35,000 × g for 45 min. The supernatants were loaded onto 5 mL His-Trap Ni-NTA columns. The columns were washed extensively with wash buffer B (20 mM Na-phosphate (pH 7.5), 100 mM NaCl, 30 mM imidazole), and proteins were eluted using a linear gradient from 0% to 100% elution buffer B (20 mM Na-phosphate (pH 7.5), 100 mM NaCl, 250 mM imidazole) over 8 column volumes. The purity of eluent fractions was checked by SDS-PAGE, and fractions containing proteins of interest were pooled and concentrated by Amicon Ultra centrifugal filter units. The proteins were further purified by size exclusion chromatography on a Superdex 75 16/200 column with 20 mM Na-phosphate (pH 7.5) and 100 mM NaCl buffer with flow rate of 2 mL/min. The purity of eluent fractions was checked by SDS-PAGE, and pure fractions were pooled. The concentrations were measured by Bradford assay. Aliquots were frozen and stored at −80 °C.

For purification of ^15^N/^13^C-labeled MprE7-TH1 peptide, plasmid DNA containing gene encoding N-His_6_-MprE7-TH1 (20 ng) was transformed in *E. coli* BL21(DE3). Colonies were grown under kanamycin selection on LB agar media for 16 h. Colonies on the plate were resuspended in 4 mL minimal media (Supplementary Table 4). This inoculum was used to initiate a 1 L 1X minimal media culture supplemented with kanamycin. Cultures were incubated with shaking at 30 °C until the OD_600_ reached 0.9. Cultures were cooled at 18 °C for 1 h before induction of protein expression by adding 0.5 mM IPTG. Cultures were incubated at 18 °C, 180 rpm for 48 h.

Cultures were harvested by centrifugation (7000 × g, 25 min, 4 °C), and cell pellets were resuspended in lysis buffer A (20 mM Na-phosphate (pH 7.5), 300 mM NaCl). Unless noted, all steps of protein purification were conducted at 4 °C. Cells were lysed by sonication. The lysate was clarified by centrifugation at 35,000 × g for 45 min. The supernatant was loaded onto a 5 mL His-Trap Ni-NTA column. The column was washed extensively with wash buffer B (20 mM Na-phosphate (pH 7.5), 300 mM NaCl, 30 mM imidazole), and protein was eluted using a linear gradient from 0% to 100% elution buffer B (20 mM Na-phosphate (pH 7.5), 100 mM NaCl, 250 mM imidazole). The purity of eluent fractions was checked by SDS-PAGE. Fractions containing protein with desired purity were pooled, incubated with 1 U/µL thrombin to remove N-His_6_ tag, and desalted by dialysis in IEX buffer A (20 mM Na-phosphate (pH 7.5), 100 mM NaCl) overnight. The dialyzed protein sample was then loaded to a 5 mL Hi-Trap Q column, washed with two column volumes of IEX buffer A and protein eluted with an increasing linear gradient of IEX buffer B (20 mM Na-phosphate (pH 7.5), 500 mM NaCl). Eluted protein fractions were further purified by size exclusion chromatography on a Superdex 75 16/200 column with IEX buffer A. The purity of eluent fractions was checked by SDS-PAGE. The concentrations were measured by Bradford assay. The pure fraction with the highest concentration was used to obtain NMR data. Aliquots were frozen and stored at −80 °C for future use.

For the ^15^N-labeled sample, ^15^NH_4_Cl [Cambridge Isotope Labs #NLM-467] was used as the sole nitrogen source. For the double-labeled ^15^N/^13^C sample, ^15^NH_4_Cl was used as the sole nitrogen source, and U-^13^C_6_-labeled glucose was used as the sole carbon source [Cambridge Isotope Labs #CLM-1396].

### Enzymatic assays

For time-course experiments to monitor the bromination of different substrate peptides by SrpI, all experiments were performed in triplicate. Assays were conducted in a total volume of 300 µL, comprising of 50 mM HEPES-Na (pH 7.5), 20 mM KBr, 25 µM FAD, 625 µM NAD^+^, 6.25 mM Na_2_HPO_3_, 5 µM flavin reductase (RebF), 3 µM phosphite dehydrogenase (PTDH), 25 µM substrate peptide (MBP-SrpE-TH1 and mutants thereof), 5 µM SrpI, and 10 ng catalase. All components were added and incubated at 30 °C for 5 min, and the reactions were initiated by the addition of the flavin reductase RebF. The reactions were allowed to proceed at 30 °C. At 0, 1, 2, 4, and 6 h, 50 mL reaction aliquots were withdrawn and quenched by the addition of 1 mL 3 N HCl. After a brief centrifugation to remove precipitates, the quenched reaction was neutralized by the addition of 1 mL 3 N NaOH. The reaction aliquots in microcentrifuge tubes were centrifuged for 10 min at 16,000 × g to remove precipitates, and the supernatant transferred to a fresh microcentrifuge tube. To this was added 1 mL of 0.1 mg/mL Glu-C protease followed by incubation at 30 °C for 16 h. To the reaction tube was then added 50 mL MeOH and the precipitates were removed by centrifugation at 16,000 × g for 30 min. The supernatants were withdrawn to high-performance liquid chromatography (HPLC) vials.

For end-point experiments to monitor the bromination of different substrate peptides by SrpI, all experiments were performed in triplicate. Assays were conducted in a total volume of 200 µL, comprising of 50 mM HEPES-Na (pH 7.5), 20 mM KBr, 25 µM FAD, 625 µM NAD^+^, 6.25 mM Na_2_HPO_3_, 5 µM flavin reductase (RebF), 3 µM phosphite dehydrogenase (PTDH), 25 µM substrate peptide (MBP-SrpE-TH1 and mutants of the NLTEEQL sequence thereof), 5 µM SrpI, and 10 ng catalase. All components were added and incubated at 30 °C for 5 min, and the reactions were initiated by the addition of the flavin reductase RebF. The reactions were allowed to proceed at 30 °C. After 6 h, reactions were quenched by the addition of 2 mL 6 N HCl. After a brief centrifugation to remove precipitates, the quenched reaction was neutralized by the addition of 2 mL 6 N NaOH. The reaction aliquots in microcentrifuge tubes were centrifuged for 10 min at 16,000 × g to remove precipitates, and the supernatant transferred to a fresh microcentrifuge tube. To this was added 2 mL of 0.1 mg/mL Glu-C protease followed by incubation at 30 °C for 16 h. To the reaction tube was then added 200 mL MeOH and the precipitates were removed by centrifugation at 16,000 × g for 30 min. The supernatants were withdrawn to HPLC vials.

To generate the calibration curve for the SrpE-TH1 substrate peptide, 2.5 µM, 6.25 µM, 12.5 µM, 25.0 µM, and 50.0 µM MBP-SrpE-TH1 protein was incubated at 30 °C for 6 h in 50 mM HEPES-Na (pH 7.5) buffer in 200 µL assay volume followed by treatment with HCl, NaOH, and Glu-C as described above. All treatments were performed in triplicate.

For liquid chromatography/mass spectrometry (LC/MS) experiments, the abundances of the halogenated product peptide and the left-over substrate peptide after the digestion with Glu-C were analyzed by Agilent 6530 C time of flight (ToF) mass spectrometer equipped with an electrospray ionization (ESI) source coupled to an Agilent 1260 HPLC. Mass spectrometry data were collected in the positive ionization mode in the MS^1^ mass range *m/z* 400–2000 Da and MS^2^ mass range *m/z* 50–2000. Chromatography was performed using Agilent Poroshell 120 2.7 μm C18 reversed-phase column (100 × 4.6 mm) at a flow rate of 0.3 mL/min using the followed solvents for the mobile phase: solvent A– H_2_O + 0.1% v/v formic acid; solvent B– MeCN + 0.1% v/v formic acid. The chromatography elution profile was as follows: 5% solvent B from 0 to 5 min, linear gradient to 100% solvent B from 5 to 18 min, 100% solvent B from 18 to 22 min, linear gradient to 5% solvent B from 22 to 24 min, and 5% solvent B from 24 to 30 min. MS data were acquired from between 5 min and 24 min. Area under the extracted ion chromatograms for the [M + 2H]^2+^ ions for the Glu-C digested fragments for the substrate and product peptides were used to determine the extent of product formation in the enzymatic assays.

For MALDI-ToF MS experiments, reactions samples were digested with Lys-C protease at 30 °C for 2 h. The reaction mixture was then desalted using C_18_ ZipTips and spotted on a MALDI target using 2 µL saturated sinapinic acid in 7:3:0.1 MeCN:H_2_O:TFA solvent for analysis by a Bruker Daltonics rapifleX MALDI-ToF mass spectrometer in reflectron positive ionization mode. The data were analyzed using flexAnalysis software.

### NMR data acquisition, titration, and chemical shift assignments

All proteins were exhaustively dialyzed into NMR buffer (100 mM NaCl, 20 mM sodium phosphate pH 7.5, 10% D_2_O). Unless stated otherwise, all NMR experiments were recorded at 25 °C in 3 mm NMR tubes in a cryoprobe equipped Bruker Avance III HD 800 MHz spectrometer at the Georgia Tech NMR Center. All data were processed in NMRPipe v11.5^61^ and analyzed in NMRFAM-SPARKY v3.19^62^.

For titration experiments, five different samples each containing 42.1 μM MprE7-TH1 with increasing MprE7-TH1:SrpI molar ratios from 1:0 to 1:1 were prepared (Supplementary Table 5). NMR samples for titration contained FAD at 1:1 molar ratio with SrpI. 2D ^1^H-^15^N amide SOFAST-HMQC spectra (pulse sequence IBS_SOFAST.x) were acquired with 50% non-uniform sampling (NUS) using the Poisson Gap sampling method^63^ with 256 scans and 0.45 s recycle delay with 65 ms and 44 ms acquisition times in the direct ^1^H and indirect ^15^N dimension, respectively^64^. NUS schedules were prepared using the nus@HMS generator v3. Chemical shift assignments of the SrpI bound states of MprE7-TH1 were determined by comparing free and bound state peaks in 3D HNCACB, CBCACONH, and HNCO experiments. NMR titration spectra were processed with 4 Hz and 10 Hz Lorentzian line broadening in the direct and indirect dimensions, respectively, and analyzed using the TITAN v1.6-12-g9041 2D lineshape analysis program with a two-state binding model^35^. A total of seven residues (A66, L70, S71, D72, D74, L75, and G76) (Supplementary Figs. 20–26) exhibiting slow exchange behavior between free MprE7-TH1 and bound MprE7-TH1/SrpI states were fit, which resulted in similar fitted apparent dissociation constant (K_D_) and off rate (k_off_) values, suggesting the residues experienced the same SrpI binding event (Table S7). Final interaction parameters were determined from 200 steps of bootstrap analysis of all eight MprE7-TH1 residues: apparent K_D_ = 24.9 ± 0.6 μM and k_off_ = 3.7 ± 0.5 s^−^^1^. Chemical shift perturbations (CSPs, p.p.m.) were determined from weighted average of amide ^1^H and ^15^N chemical shift changes for free MprE7-TH1 versus SrpI bound states of MprE7-TH1 using the equation Δ*δ*^NH^ = [1/2 (Δ*δ*_H_^2^ + Δ*δ*_N_^2^/25)]^½^^65^. For CSP analysis, chemical shifts of “state A” NMR peaks in the unbound MprE7-TH1 were compared to the chemical shifts of “state B” NMR peaks in the SrpI bound states of MprE7-TH1. Peak intensity ratio analysis (I_bound_/I_free_) of SrpI bound MprE7-TH1 relative to unbound MprE7-TH1 was performed at the 1:1 molar ratio of MprE7-TH1/SrpI titration point. At this titration point, MprE7-TH1 and SrpI are both at 42.1 μM. For peaks showing two state behavior, a comparison of different states was performed: “state A” in SrpI bound MprE7 vs “state A” of free MprE7, “state B” of SrpI bound MprE7 vs “state B” of free MprE7, and “state B” of SrpI bound MprE7 vs “state A” of free MprE7 (here, peak heights for the two states were compared between different HSQC spectra). Peak intensities ratios were determined by comparison of corresponding “peak heights” in Sparky. No normalization was performed. For determination of % “state A” peak of MprE7-TH1 in the absence and presence of SrpI enzyme, the following formula was used (here, peak heights were compared within the same HSQC spectra):

1 $$\%\, {state} \, A=\frac{{peak} \, {height} \, {state} \, {A}}{{peak} \, {height} \, {state} \, {A}+{peak} \, {height} \, {state} \, {B}} \times 100$$

For chemical shift assignments, uniformly double-labeled ^15^N/^13^C MprE7-TH1 was prepared in NMR buffer at concentrations of 200 to 400 μM. Backbone resonances were assigned using sequential assignment strategies using standard triple-resonance experiments with about 20% Poisson Gap sampling schedule and reconstructing with istHMS^66^: 3D HNCO (Bruker pulse sequence hncogpwg3d), 3D HNCACB (Bruker pulse sequence hncacbgpwg3d), 3D CBCACONH (Bruker pulse sequence cbcaconhgpwg3d), 3D NOESY (Bruker pulse sequence noesyhsqcf3gpwg3d) and 3D TOCSY (Bruker pulse sequence dipsihsqcf3gpsi3d). Acquisition times were 92 ms in ^1^H, 11 ms in ^15^N, 20 ms in ^13^CO and 10/5 ms in ^13^C_α,β_. Recycle delay was set to 1 s in all experiments. Chemical shift assignments of the SrpI bound states of MprE7-TH1 were determined by comparing free and bound state peaks in 3D HNCACB, CBCACONH, and HNCO experiments.

Backbone amide ^1^D_NH_ RDCs were obtained through 2D ^1^H-^15^N TROSY/anti-TROSY based inphase-antiphase (IPAP) experiments (Bruker pulse sequence trosyetf3gpiasi) acquired with 8 scans, 1 s recycle delay, and 30 ms in the indirect ^15^N dimension. Experiments were acquired on both isotropic (unaligned) and anisotropic (aligned) samples. ^1^D_NH_ RDC values were extracted from the difference between peak positions in the unaligned/aligned samples. The aligned samples contained a final concentration of 10 mg/mL Pf1 phage (ASLA Biotech #P-50-P), resulting in ^2^H quadrupolar splitting of 11.4 Hz. Back-calculated ^1^D_NH_ RDC values and Q-factors were determined using the lowest energy MprE7-TH1 NMR structure with the Bax DC server (https://spin.niddk.nih.gov/bax/nmrserver/dc/).

For structure calculations, to obtain through-space restraints for MprE7-TH1 structure calculation, 3D amide-amide NOESY experiments (Bruker pulse sequence noesyhsqcf3gpwg3d) were collected with 48 scans, 1 s recycle delay, and 350 ms mixing time. NOE cross-peaks were assigned manually in NMRFAM-SPARKY v3.19. “State A” NMR peaks in the unbound MprE7-TH1 were used by TALOS-N to determine SSI and RCI-S^2^ predictions, and towards structure calculation. Structure calculations were set up with automated Python scripts using CS-Rosetta toolkit v3.3^35^. We first used TALOS-N to determine SSI and RCI-S^2^ predictions and used the MrpE7-TH1 sequence and chemical shift values to pick structural fragments of amino acid lengths 3 and 9. We then used the protein sequence, 3-mer/9-mer fragments, backbone chemical shifts, NOEs, and ^1^D_NH_ RDC measurements as input for the abrelax CS-Rosetta protocol. A total of 293 chemical shift values were used to derive 122 dihedral angle restraints via TALOS-N. A total of 75 non-redundant amide-amide NOEs were used. A total of 59 ^1^D_NH_ RDC measurements ranging from residues 15 to 87 were used in the structure calculation. From the 20,000 models calculated, the 10 lowest energy models were selected that fit the experimental NOE and RDC data. Final structures were validated with MolProbity.

### Molecular dynamics (MD) simulations

All-atom MD simulations in explicit solvent were carried out in GROMACS version 2022.4 using the CHARMM36 force field and TIP3P water model^45^. Simulations were set up using the CHARMM-GUI solution builder. An integration time step of 2 fs was used with coordinates output every 10 ps. The LINCS algorithm was used to constrain H-bonds. The thermodynamic ensemble was nPT, where the temperature was kept constant at 298.15 K. Temperature was maintained using the Nosé–Hoover coupling method with a tau-t of 1 ps. For pressure coupling, an isotropic Parrinello–Rahman method with a tau-p of 5 ps and a compressibility of 4.5 × 10^−^^5^ bar^−^^1^ was used. Short range interactions were treated with a Verlet cutoff scheme with 10 Å electrostatic and van der Waals cutoffs and long-range electrostatics were treated using the particle mesh Ewald method. Periodic rectangular boundaries were used. For MD simulations of MrpE7-TH1, the lowest energy NMR structure (PDB ID 8TB1) was used. Four independent trajectories (differential initial velocities) for each molecule were acquired for 500 ns. Commands used for MD simulation analysis are available in the Supplementary Methods.

### Homology modeling of SrpI, docking of MprE7-TH1/SrpI, and MD simulations of the MprE7-TH1/SrpI complexes

For AlphaFold homology modeling, the FASTA sequence of SrpI was used as input to generate an AlphaFold2 model within ChimeraX. The final model of SrpI was chosen based on the best pLDDT score^67^.

For HADDOCK docking to generate a working MprE7-TH1/SrpI working model, the lowest energy NMR structure of MprE7-TH1 was docked onto the AlphaFold model of SrpI using default parameters in HADDOCK 2.4^68^, except that the C-terminal region of MprE7-TH1 (residues 60–87) were treated as a flexible entity. MprE7-TH1 regions demonstrating CSPs greater than the mean CSP (residues 65–87; Fig. 4) upon titration with SrpI were used as constraints to guide docking near the active site residues of SrpI (K84). The best docked structure was considered the model with the lowest HADDOCK score. The lowest scoring model showed two important features: W87 of MprE7-TH1 was oriented towards the active site residue K84 of SrpI, and MprE7-TH1 residues L70 and L75 were oriented towards the SrpI loop comprising of residues 105–110.

For MD simulations of MprE7-TH1/SrpI complexes, all-atom MD simulations performed in explicit solvent were carried in GROMACS version 2022.4 using the CHARMM36 force field and TIP3P water model^45^. Simulations were set up using the CHARMM-GUI solution builder as mentioned above. The FAD co-factor was docked into SrpI based on homology with MibH (PDB ID 5UAO)^18^ and was parameterized using CHARMM-GUI. Trajectories were acquired for 100 ns and analyzed in GROMACS. For simulations with mutant SrpI (P105G/Q106A/Q107G/V108A/S109G), mutations were introduced CHARMM-GUI prior to building the simulation.

### Circular dichroism (CD) spectropolarimetry

CD was performed on a Jasco J-815 spectropolarimeter with purified MprE7 leader peptide (MprE7-LP; 50.5 µM protein concentration) prepared in 1X PBS (10 mM Na_2_HPO_4_, 1.8 mM KH_2_PO_4_, 137 mM NaCl, 2.7 mM KCl, pH 7.5). Thermal denaturation experiments were performed by increasing the temperature from 10 to 90 °C in 1 °C/min increments using a Neslab RTE-111 (Thermo Scientific) circulating water bath and monitoring the profiles between 200 and 300 nm in a 0.1 cm path length cuvette. Three replicate spectra, scanned from 300 to 200 nm at rate of 500 nm/min, were averaged at the designated temperature. Our attempts to acquire reliable data below 200 nm were not successful due to voltage limits of the instrument and available nitrogen flow rate. Each averaged spectrum was background-corrected and converted to molar circular dichroism (Δ*ε*):

2 $$\left[\Delta \varepsilon \right]=\frac{{M}_{{res}}\times {\theta }_{{obs}}}{10\times d\times C\times 3298}$$

where the mean residue mass ${M}_{{res}}={Molecular\; weight}/(n-1)$ with $n$ is the number of residues in the peptide; ${\theta }_{{obs}}$ is the observed ellipticity (degrees) at wavelength λ, $d$ is the pathlength (cm); and $C$ is the protein concentration (g/l). CD data at 25 °C from 200 to 300 nm were deconvoluted into component spectra using the BeStSel v2023 server (Supplementary Fig. 6)^24^. Melting temperature was calculated using Boltzmann sigmoid model (Supplementary Fig. 6).

### Small-angle X-ray scattering (SAXS) data acquisition and processing

SAXS datasets for MprE7-TH1 samples in buffer containing 20 mM sodium phosphate (pH7.5), 100 mM NaCl and 48 μM FAD, at three different protein concentrations, 523 μM, 486 μM, and 448 μM, were acquired using an in-house Rigaku BioSAXS2000^nano^ Kratky camera system with X-rays generated by a Rigaku MM007 rotating anode at a wavelength of 1.54 Å. The system includes OptiSAXS confocal max-flux optics that are designed specifically for SAXS and a HyPix-3000 Hybrid Photon Counting detector. The Kratky block attenuation was 22% for a beam diameter of ~100 μm. The sample capillary-to-detector distance was 495.5 mm and was calibrated using silver behenate powder (The Gem Dugout, State College, PA). The useful *q*-space range (4πsinθ/λ, where 2θ is the scattering angle) was generally from *q*_min_ = 0.008 Å^−^^1^ to *q*_max_ = 0.6 Å^−^^1^.

Protein samples were loaded using the autosampler onto a quartz capillary flow cell, mounted on a stage maintained at 22 °C and aligned in the X-ray beam. The sample cell and full X-ray flight path, including beam stop, were kept in vacuo (< 1 × 10^−^^3^ torr) to eliminate air scatter. The Rigaku SAXSLab software was programmed for automated data collection of each protein sample and matched buffers, with cleaning with 1 M NaOH prior to the start of the run and with water and ethanol between samples. Data reduction, including image integration and normalization, and background buffer data subtraction were carried out using the SAXSLab software. Six 10 min images from protein and buffer samples were collected and averaged after ensuring that no X-ray radiation damage had occurred. The sample SAXS were buffer subtracted to get the final data used for further analysis (Supplementary Figs. 7–10). In the three concentrations tested, the Rg values agree with a monomer of MprE7-TH1 ( ~ 18.4 Å) and show no concentration dependent size changes. The experimental Rg value agrees with the calculated size of 17.3 Å derived from the NMR models. The data files were analyzed for Guinier *R*_g_, maximum particle dimension (*D*_max_), Guinier fits, Kratky plots, and pair distance distribution function using the ATSAS software. The top-ten NMR-derived models were assessed using the ATSAS-CRYSOL and OLIGOMER programs and further refined through explicit-solvent all-atom molecular dynamics simulations with the WAXSiS server^37,38^. The theoretical scattering profiles of the constructed models were calculated and fitted to experimental scattering data using CRYSOL. Finally, the best model was manually superimposed using PyMOL v2.5.4 onto the electron density envelope computed using DENSS.

### Reporting summary

Further information on research design is available in the Nature Portfolio Reporting Summary linked to this article.

### Supplementary information

Supplementary Information

Peer Review File

Description of Additional Supplementary Files

Supplementary Movie 1

Reporting Summary

### Source data

Source Data

## Data Availability

Coordinates for the MprE7-TH1 structure have been deposited to the Protein Data Bank (PDB) with the accession code 8TB1. Other PDBs used for comparisons in Fig. 6 include 4V1T and 6MPZ. The backbone chemical shift assignments of MprE7-TH1 been deposited to the Biological Magnetic Resonance Bank (BMRB) with the accession code 31097. The SAXS data has been deposited to the SASBDB with the accession code SASDTM5. All raw data associated with the figures are provided in the provided source data file. Source data are provided with this paper.

## Code availability

Trajectories and scripts for MD simulations have been deposited to Zenodo at 10.5281/zenodo.10257781 Commands for performing MD simulations are provided in the Supplementary Information.
