# Supplementary material for: Disordered regions in proteusin peptides guide post-translational modification by a flavin-dependent RiPP brominase
Source: Nat Commun. 2024 Feb 10;15:1265. doi: 10.1038/s41467-024-45593-5 (PMC10858898; doi:10.1038/s41467-024-45593-5)
Supplement: Supplementary file 1 — Supplementary Information [file 41467_2024_45593_MOESM1_ESM.pdf]

Supplementary Information for

Disordered regions in proteusin peptides guide post-translational modification by a flavin-dependent RiPP brominase

Nguyet A. Nguyen,[1,§] FNU Vidya,[1,§] Neela H. Yennawar,[2] Hongwei Wu,[1] Andrew C. McShan,[1,*] and Vinayak Agarwal[1,3,*]

[1]School of Chemistry and Biochemistry, Georgia Institute of Technology, Atlanta, GA 30332, USA

[2]The Huck Institutes of the Life Sciences, Pennsylvania State University, University Park, PA 16802, USA

[3]School of Biological Sciences, Georgia Institute of Technology, Atlanta, GA 30332, USA

[§]Equal contribution authors

*Correspondence: andrew.mcshan@chemistry.gatech.edu; vagarwal@gatech.edu

Supplementary Figures

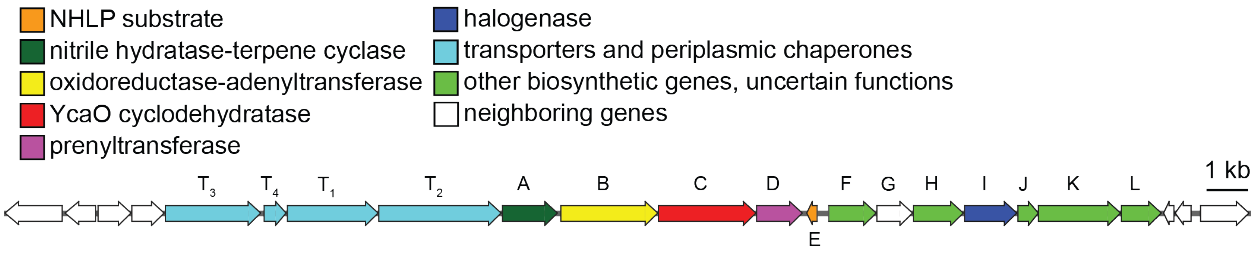

Supplementary Figure 1: The *srp* BGC detected in the Floridian marine sponge *Smenospongia aurea* metagenome contains genes *srpE* and *srpI* encoding for the proteusin substrate peptide and the brominase, respectively.

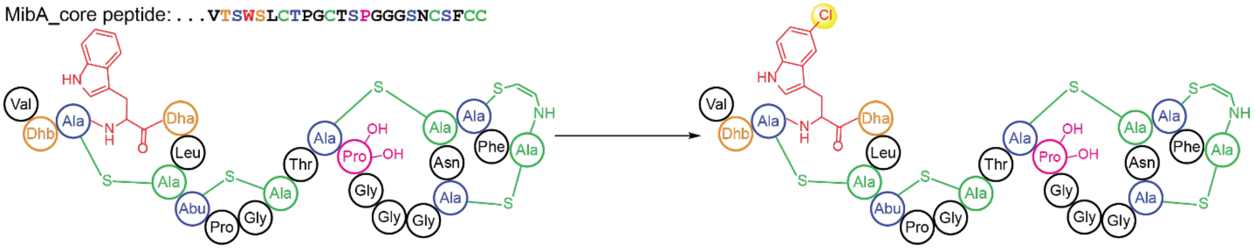

Supplementary Figure 2: Chlorination reaction catalyzed by MibH. The substrate for MibH is a highly modified RiPP core without the leader peptide. The Trp side chain of the precursor peptide MibA is chlorinated by MibH in a leader-independent manner. Dhb: Dehydrobutyrine, Dha: Dehydroalanine, Abu: Aminobutyric acid.

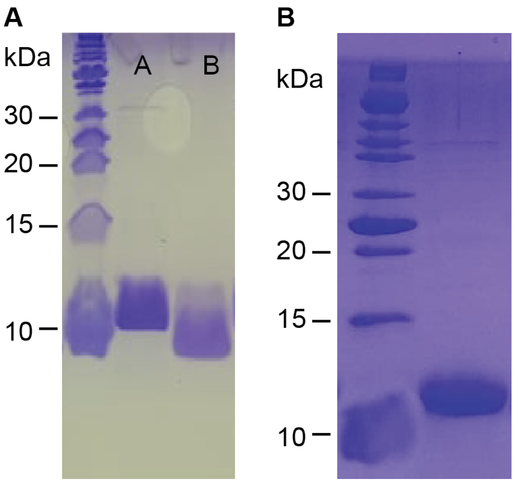

Supplementary Figure 3: **(A)** SDS-PAGE for purified MprE7-leader peptides. Lane A: His_6-MprE7-leader peptide (10.7 kDa). Lane B: MprE7-leader peptide without His_6-tag (8.8 kDa). Replicates n=1. **(B)** SDS-PAGE for purified MprE7-TH1 (His_6-MprE7-TH1: 11.6 kDa). Replicates n=1.

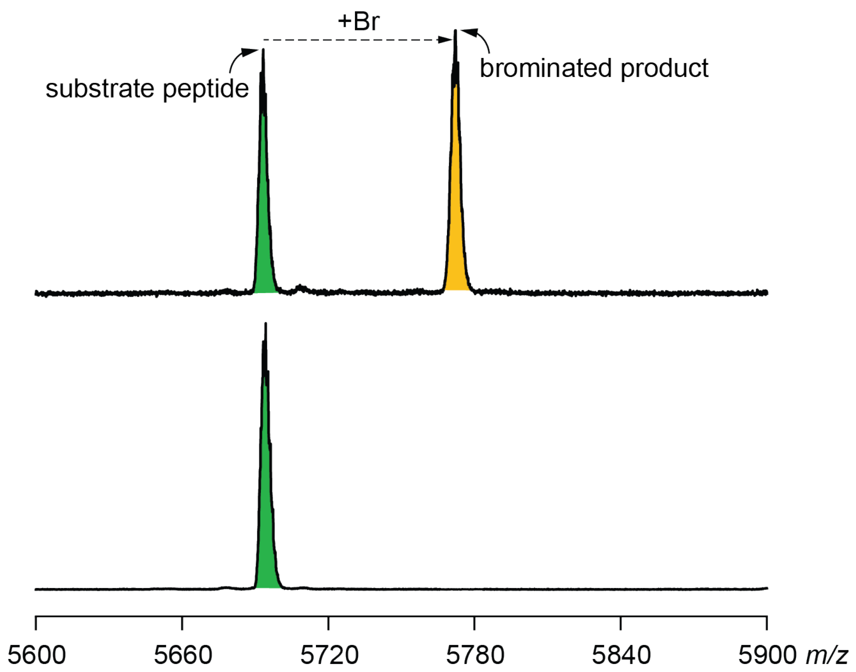

Supplementary Figure 4: MALDI-ToF MS spectra demonstrating *in vitro* bromination of MprE7-TH1 by SrpI. The bottom spectrum represents a negative control reaction in which bromide was omitted. Peaks corresponding to the unmodified substrate and the brominated product are colored in green and yellow, respectively.

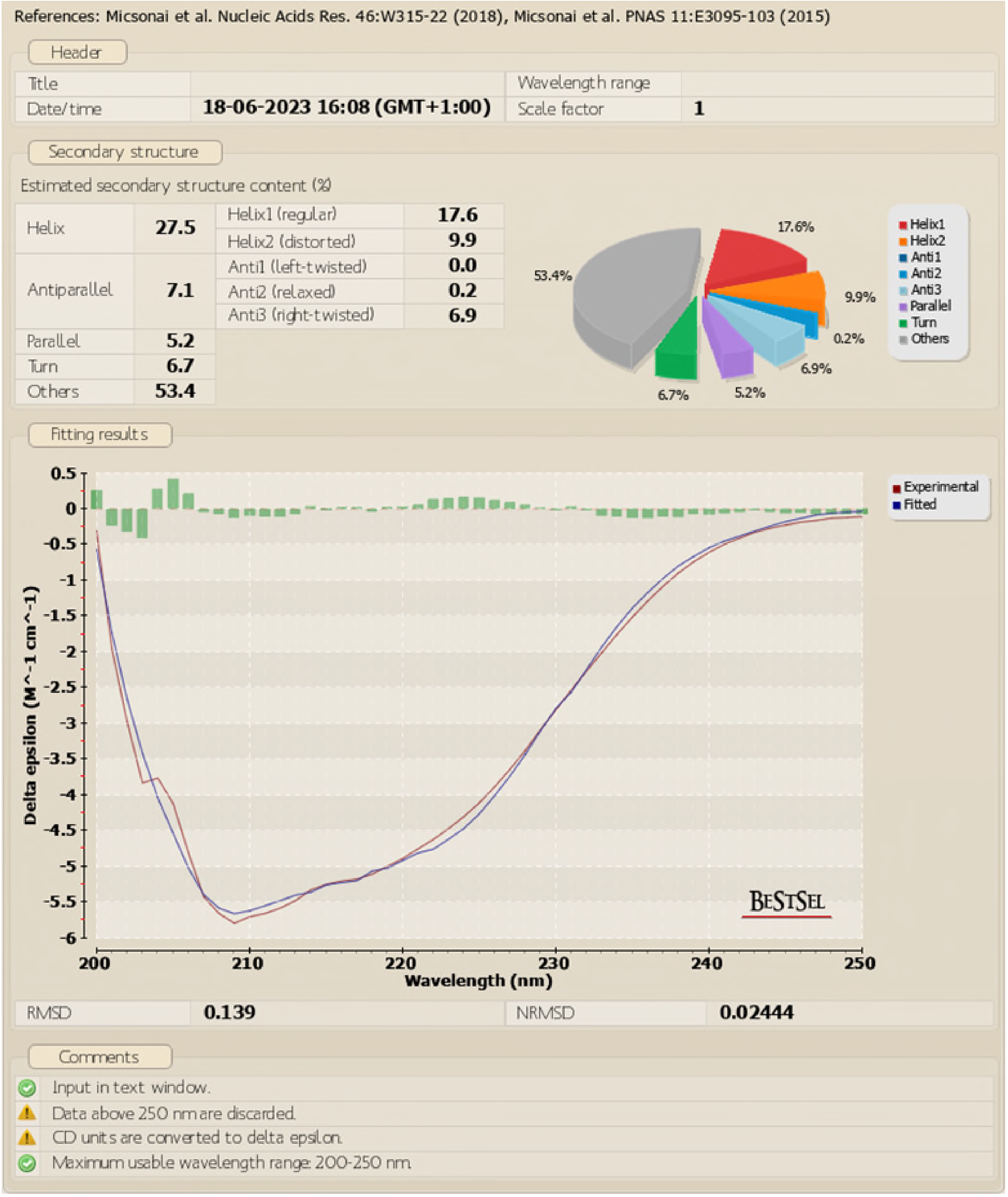

Supplementary Figure 5: Deconvolution of CD spectra at 25 ºC from 200–300 nm into component secondary structural elements using the BeStSel server (https://bestsel.elte.hu/index.php).[1] The fit is from a "single spectrum analysis", used to distinguish different types of secondary structure elements from CD spectroscopy curves. From the BeStSel website: "The secondary structure basis components of BeStSel are

derived from DSSP. Parallel and antiparallel beta-sheets are distinguished, and antiparallel beta-sheets are divided into three subgroups: left-hand twisted, relaxed, and right-hand twisted (anti1, anti2, anti3, respectively). The regular part of helices (helix1) and the distorted ends (helix2) are separated, similarly to SELCON3, however, only α-helices are counted. BeStSel sorts 310-helix to "others". The definition of turn is identical to that in DSSP.[2] The figure shows the eight basic components of BeStSel in relation to DSSP. The pie chart in the top right of the figure shows the % of different secondary structure elements of MprE7-TH1 as determined by BeStSel from the MprE7-TH1 CD spectroscopy curve.

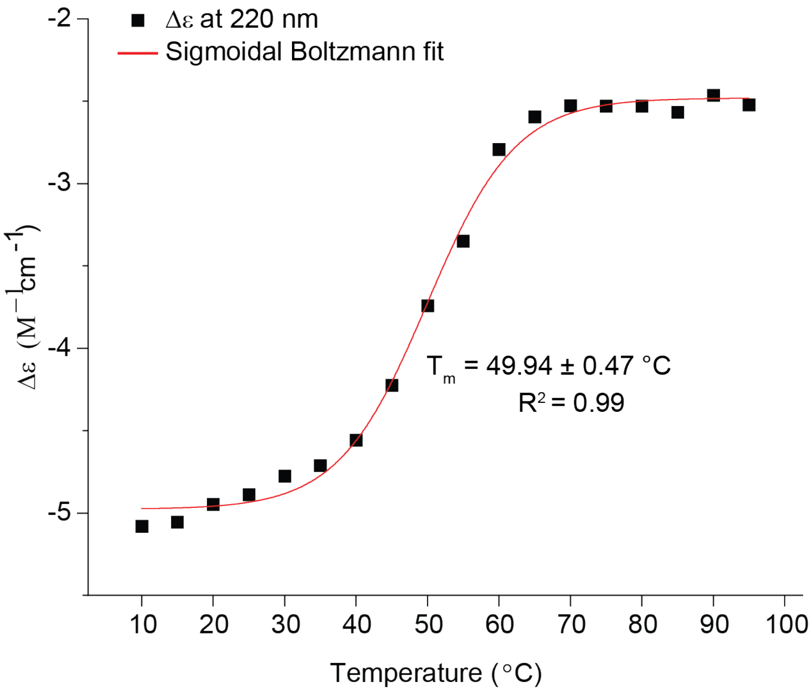

Supplementary Figure 6: Temperature-dependent denaturation curve of MprE7 leader peptide as monitored by CD spectroscopy (at 220 nm). Replicates n=1.

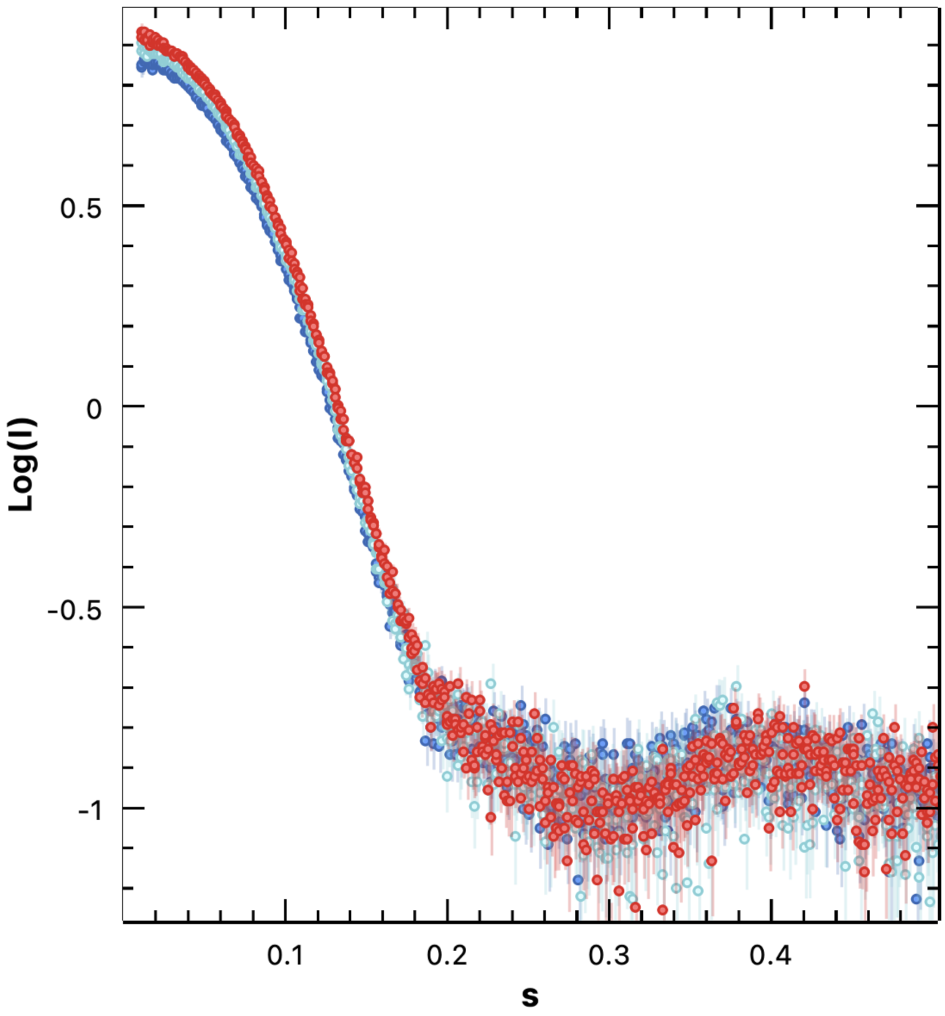

Supplementary Figure 7: Three raw SAXS data sets for MprE7-TH1, with concentrations of 523 μM in red, 486 μM in cyan, and 448 μM in blue. The measurements were performed in a buffer containing 20 mM sodium phosphate (pH 7.5), 100 mM NaCl and 48 μM FAD. Data were collected using an in-house Rigaku BioSAXS2000nano X-ray instrument. Data at each of the three concentrations were acquired over a 60-minute period, comprising six ten-minute images using an autosampler quartz flow cell. Images were subsequently averaged after confirming the absence of radiation damage. To obtain the final SAXS profiles, buffer SAXS data collected over 60 min using the same flow cell were used for reference subtraction.

(a)

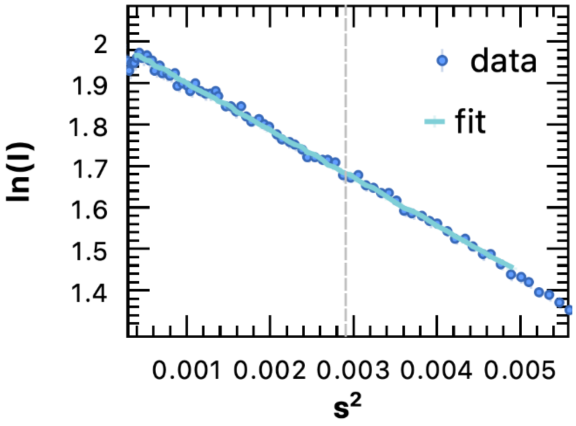

(b)

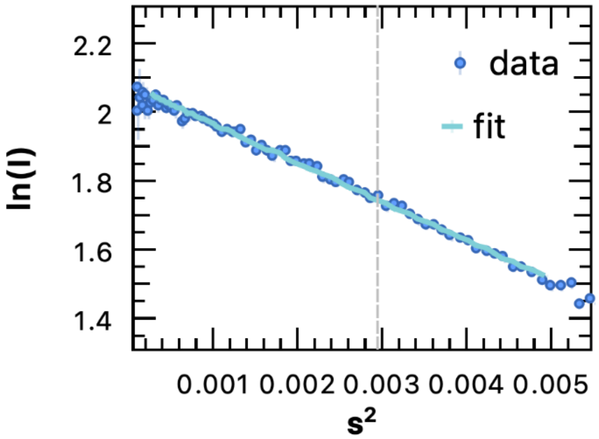

(c)

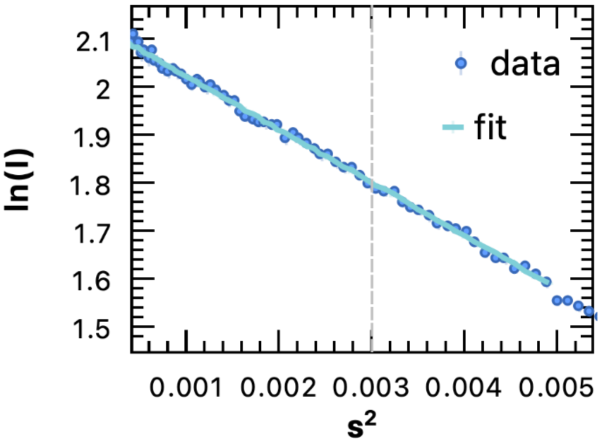

Supplementary Figure 8: An overlay of the SAXS-derived Guinier plots is presented for the three-concentration series of MprE7-TH1. SAXS raw data at concentrations of (a) 448 μM, (b) 486 μM, and (c) 523 μM are represented by blue circles, accompanied by a Guinier fit depicted as a cyan line.

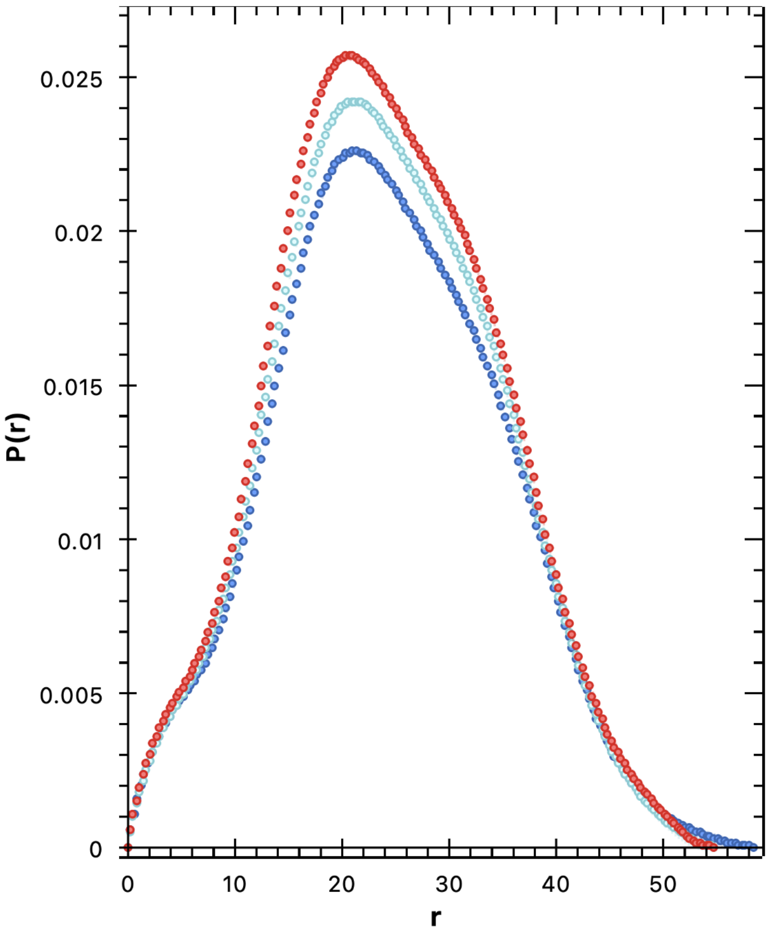

Supplementary Figure 9: The SAXS-derived pair distance distribution function P(r), plotted as a function of r. Shown here is the overlay of P(r) for the three-concentration series of MprE7-TH1 studied by SAXS. All three peak around 18.6 Å, which agrees with the Rg values obtained from the Guinier analysis. Dmax, representing the maximum diameter of the particle and observed at the point where the P(r) curve intersects the X-axis, is around 53 Å.

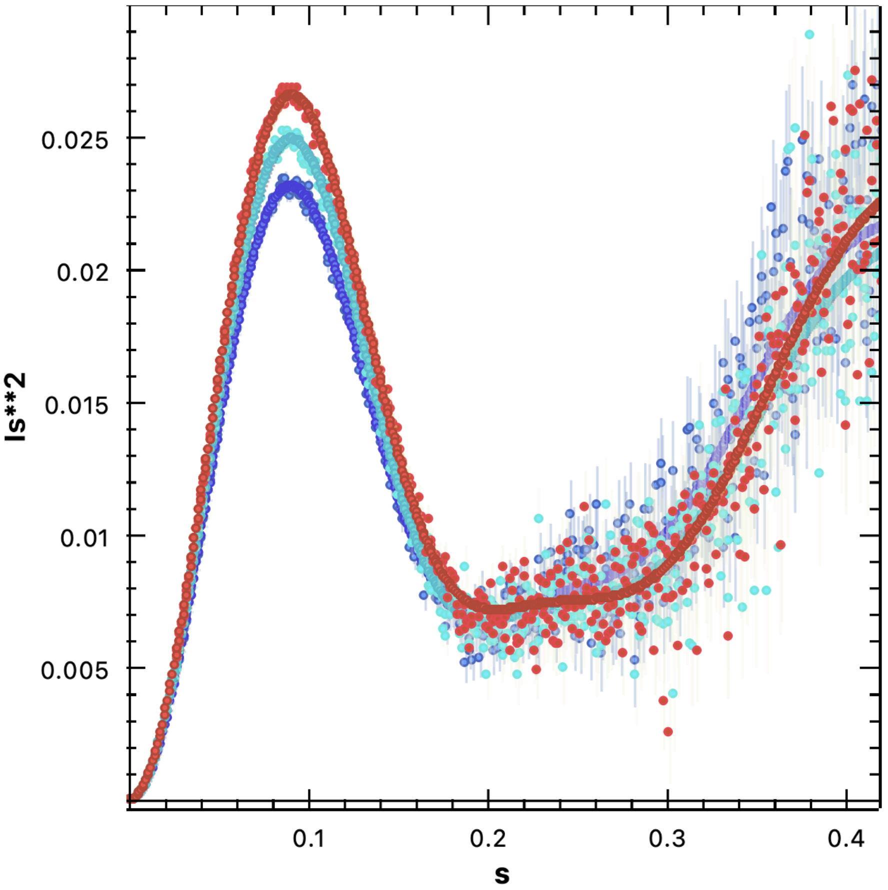

Supplementary Figure 10: The Kratky plots derived from the SAXS data of MprE7-TH1 exhibit semi-Gaussian shapes, reflecting on a mostly well folded protein with perhaps some movement in the orientation of the unstructured, flexible 12-residue C-terminal tail. Notably, the plots for the three concentrations (523 µM in red, 486 µM in cyan, and 448 µM in blue) display comparable semi-Gaussian patterns.

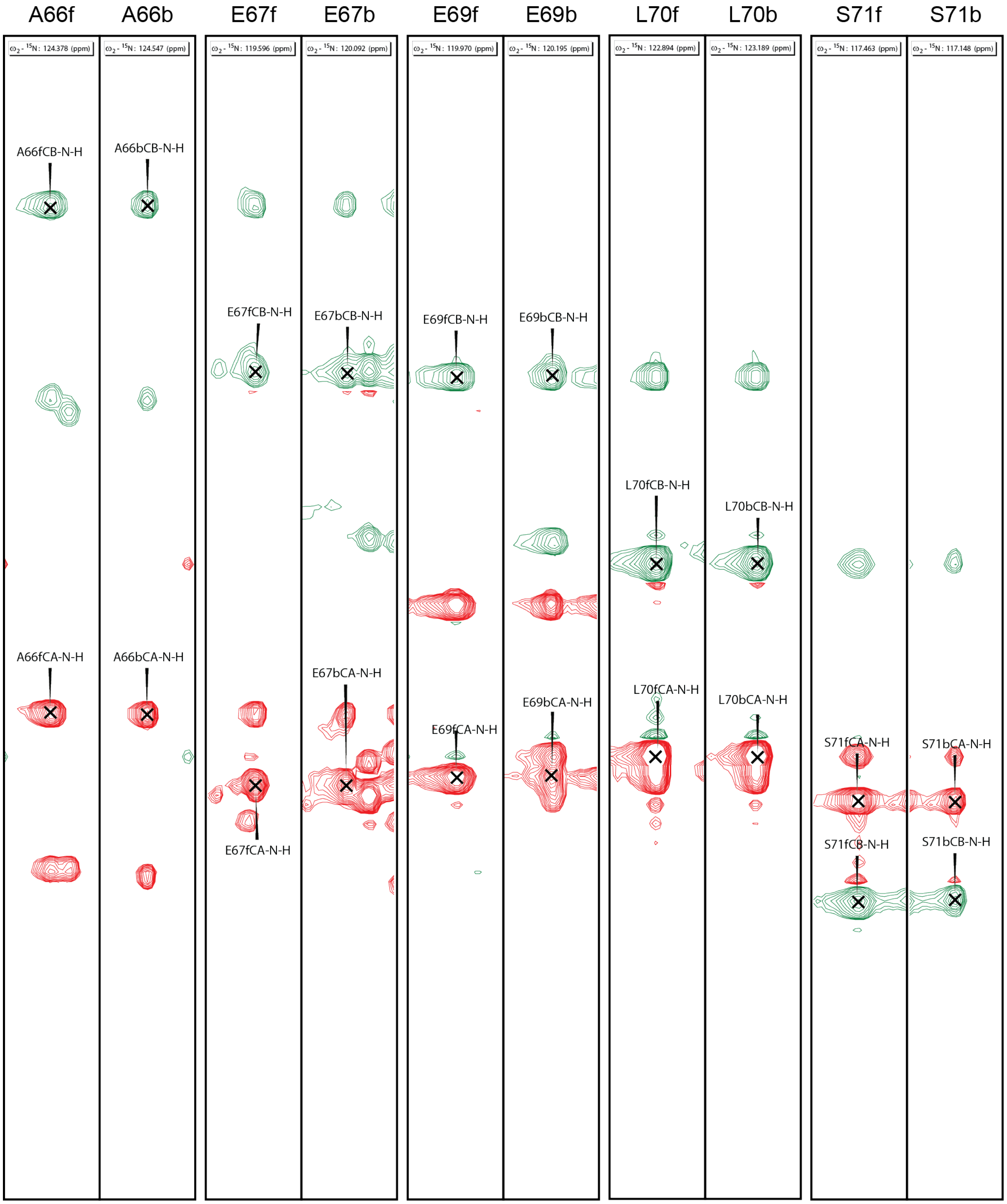

Supplementary Figure 11: HNCACB strip plots showing the assignments for the for “state A” (f) and “state B” (b) states of residues A66, E67, E69, L70, S71.

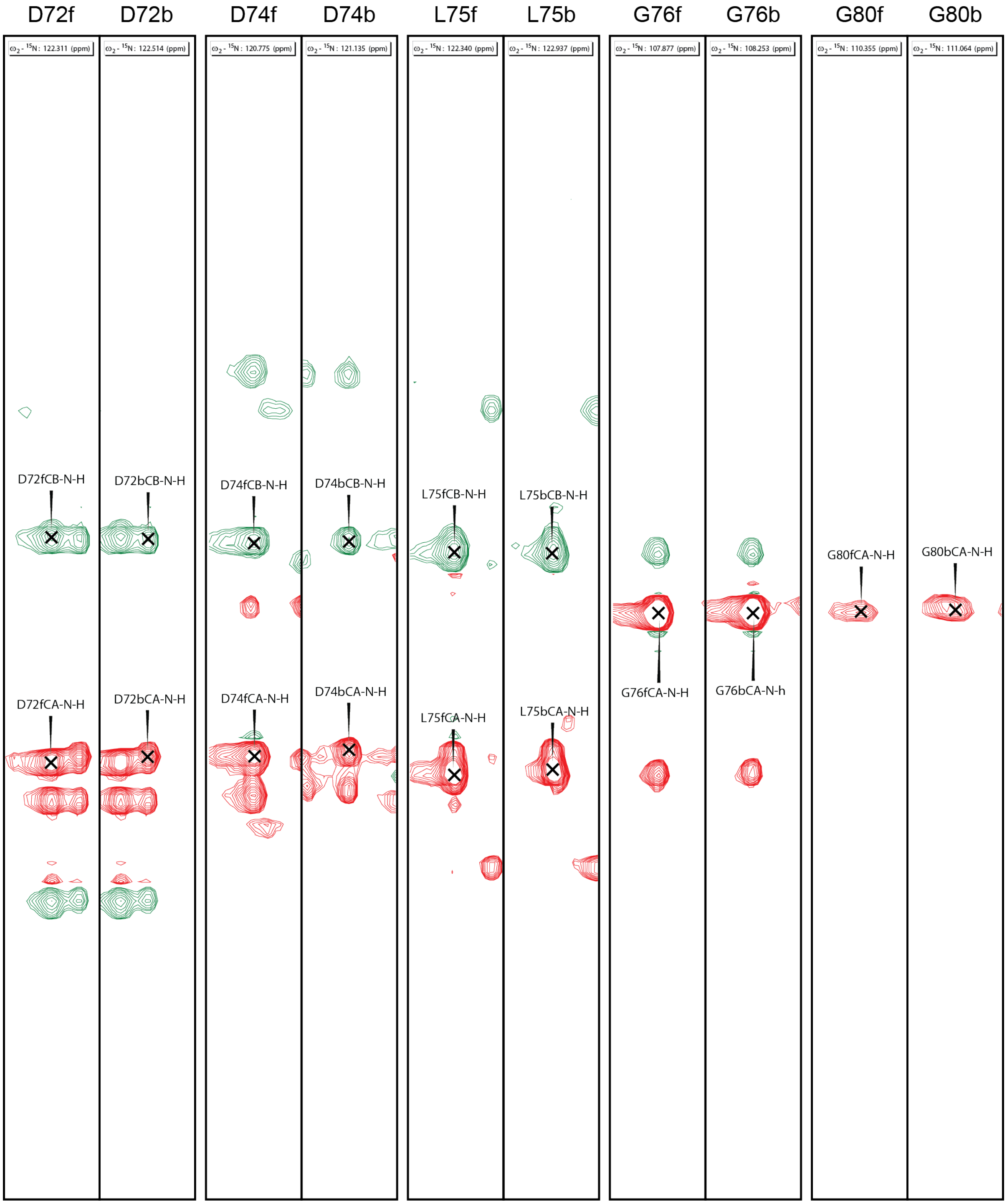

Supplementary Figure 12: HNCACB strip plots showing the assignments for “state A” (f) and “state B” (b) of residues D72, D74, L75, G76, G80.

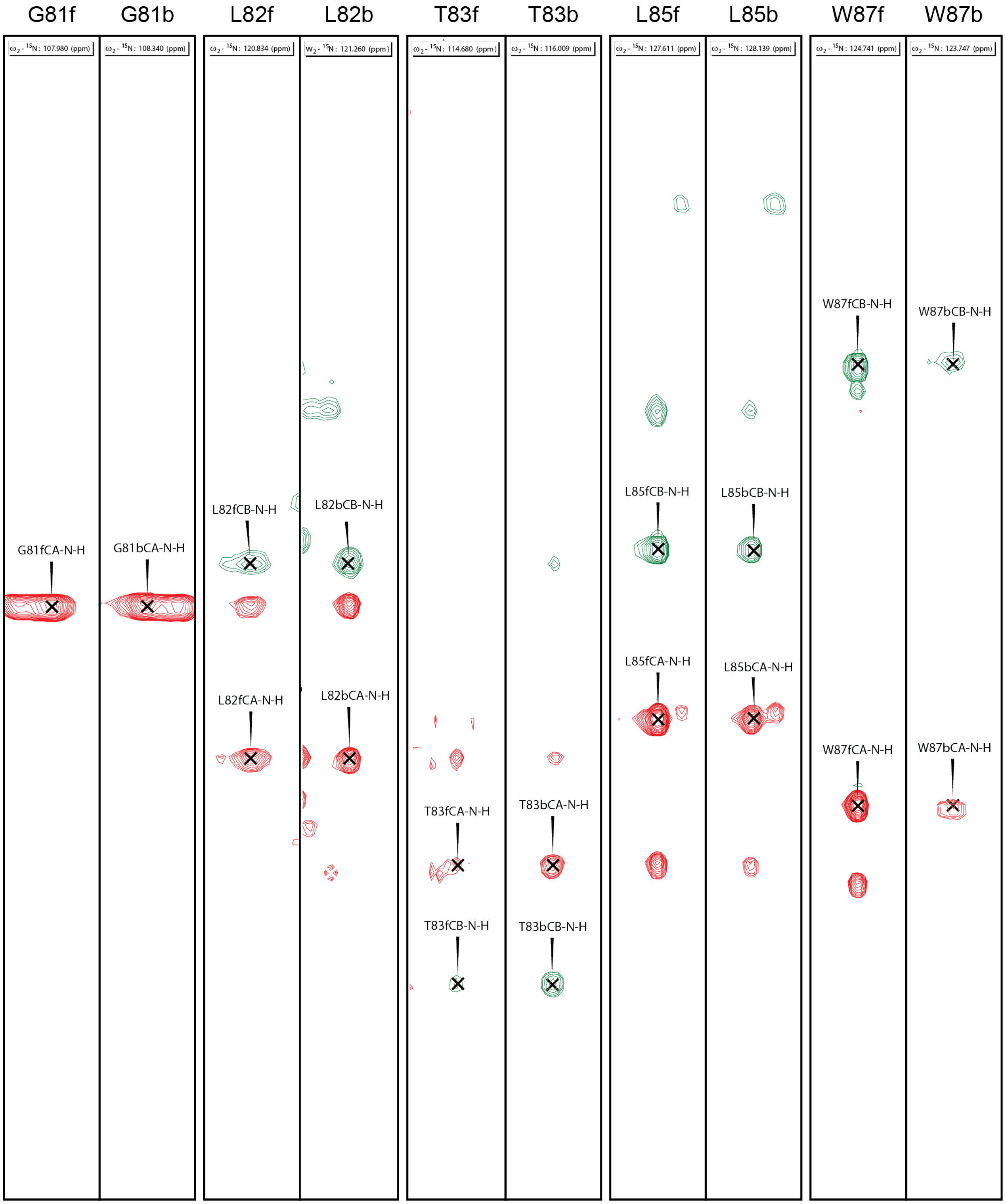

Supplementary Figure 13: HNCACB strip plots showing the assignments for the for "state A" (f) and "state B" (b) states of residues G81, L82, T83, L85, W87.

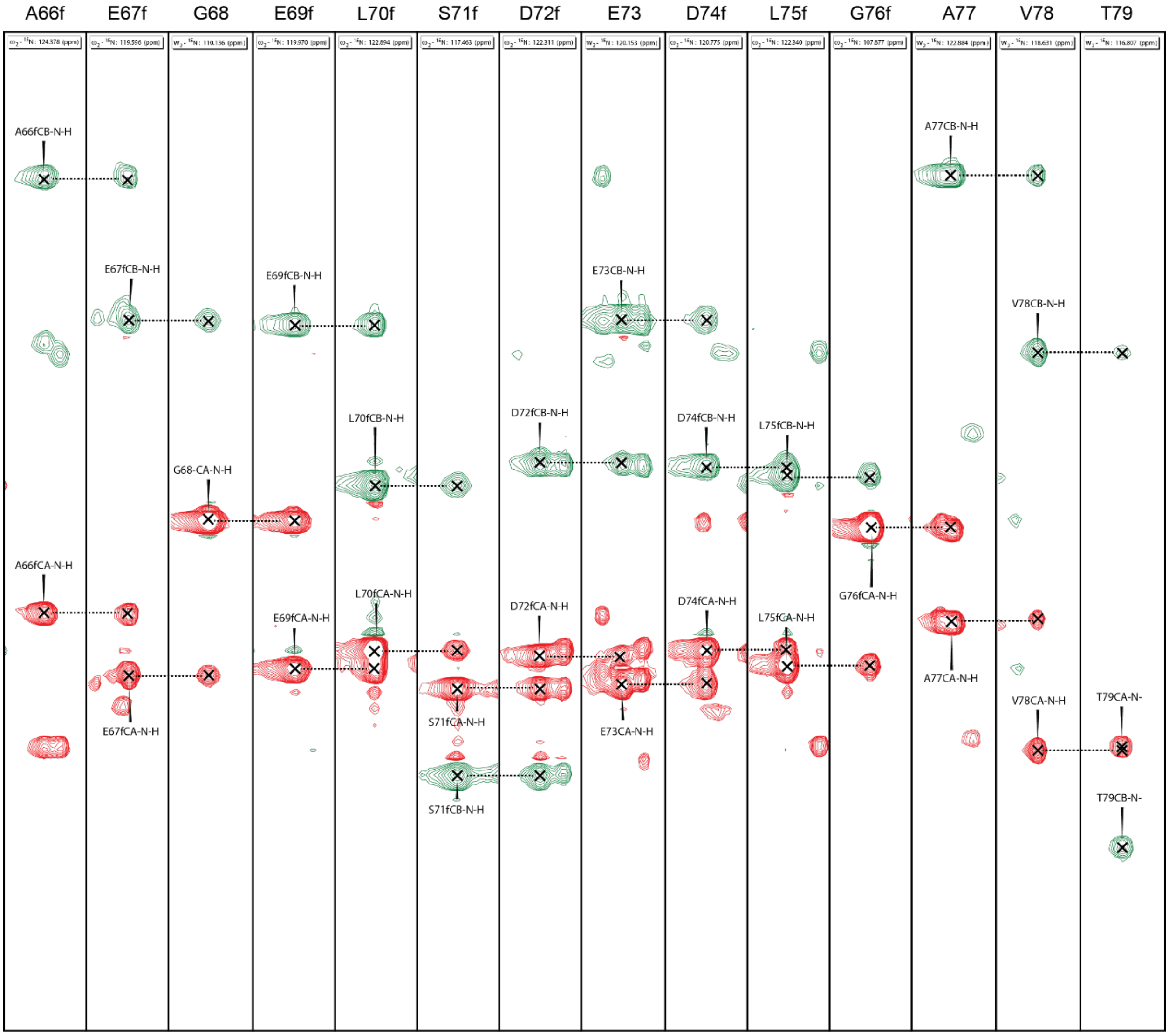

Supplementary Figure 14: HNCACB strip plots showing sequential assignments from A66 to T79.

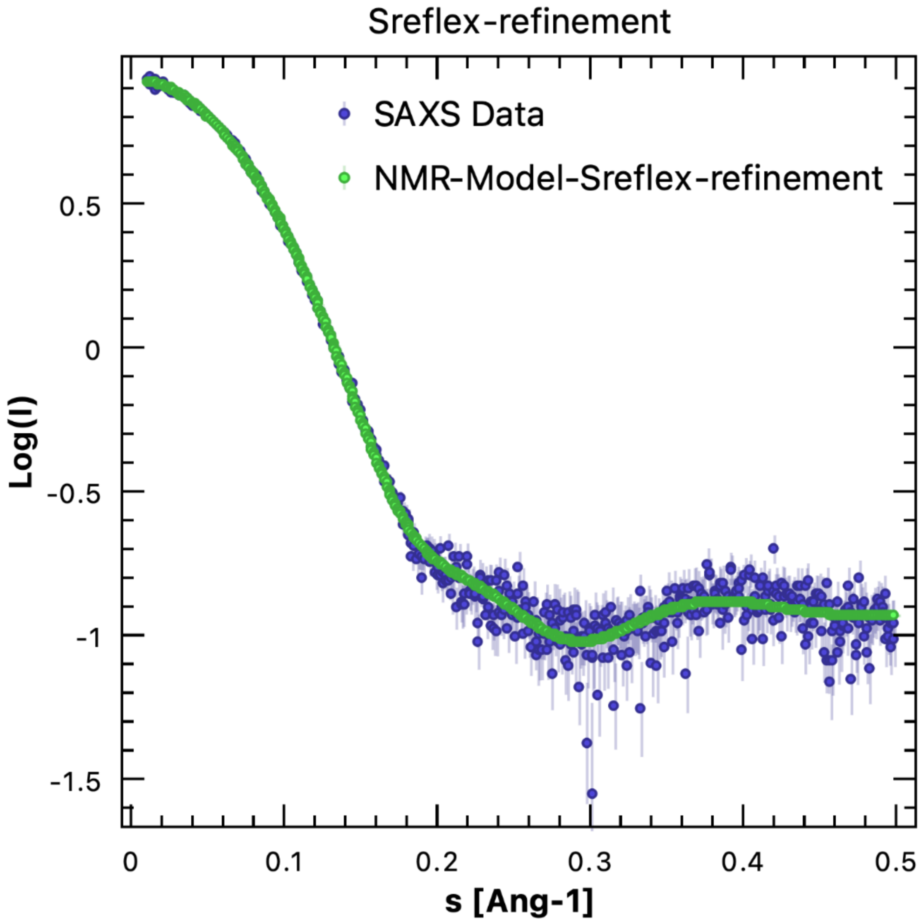

Supplementary Figure 15: Crysol program overlay of the experimental SAXS profile of MprE7-TH1 (concentration 423 μM data in purple), with the calculated SAXS profile from the best NMR model refined by the ATSAS Sreflex software (in green) has a Chi-square fit of 2.1. The calculated Rg of the model is 17.9 Å in the range calculated from the P(r) and Guinear analysis.

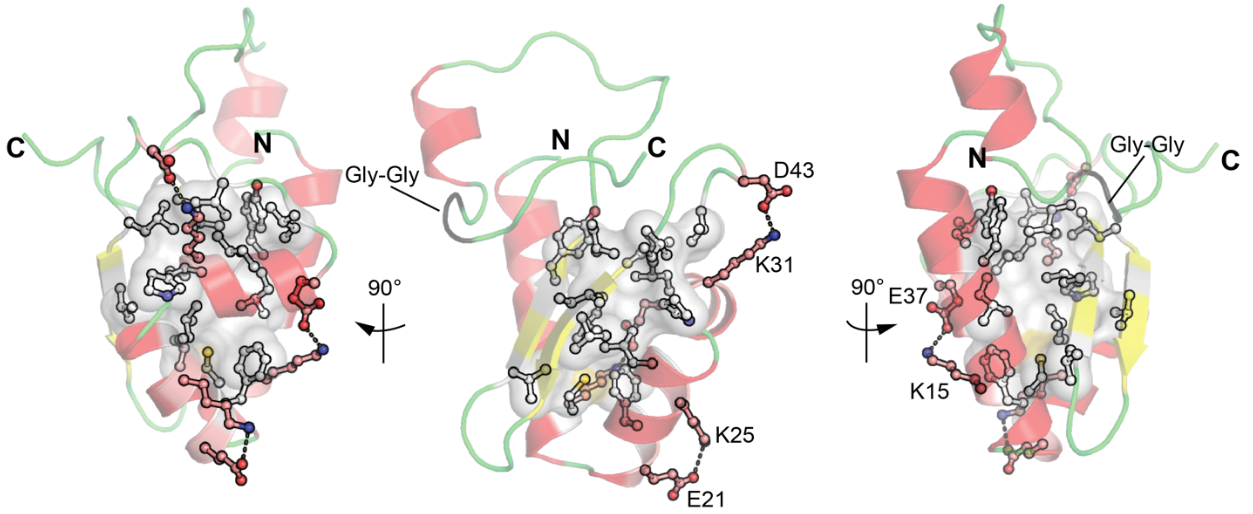

Supplementary Figure 16: Three views of the MprE7-TH1 proteusin peptide structure. Side chains of residues that constitute the hydrophobic interior core are shown in stick ball representation with carbon atoms colored grey, and the hydrophobic core visualized in surface representation. The N- and the C-termini are labeled. Salt bridges are shown as dashed lines. Residues that contribute to the formation of these salt bridges are labeled. The Gly-Gly motif that marks the leader/core boundary is labeled.

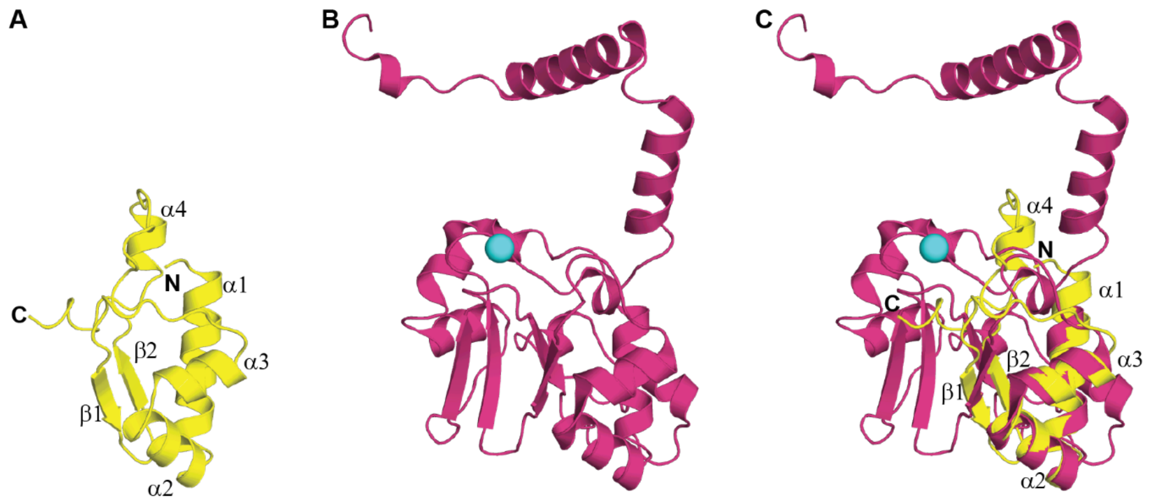

Supplementary Figure 17: **(A)** NMR structure of MprE7–TH1 (this work - PDB: 8TB1). **(B)** Crystal structure of the *Bacillus smithii* nitrile hydratase (PDB: 1V29). The cobalt metal ion is colored in cyan. **(C)** Overlay of the structures of MprE7-TH1 and the *B. smithii* nitrile hydratase showing the nearly perfect alignment between two structures at the α1–α3 and β1 of MprE7-TH1.

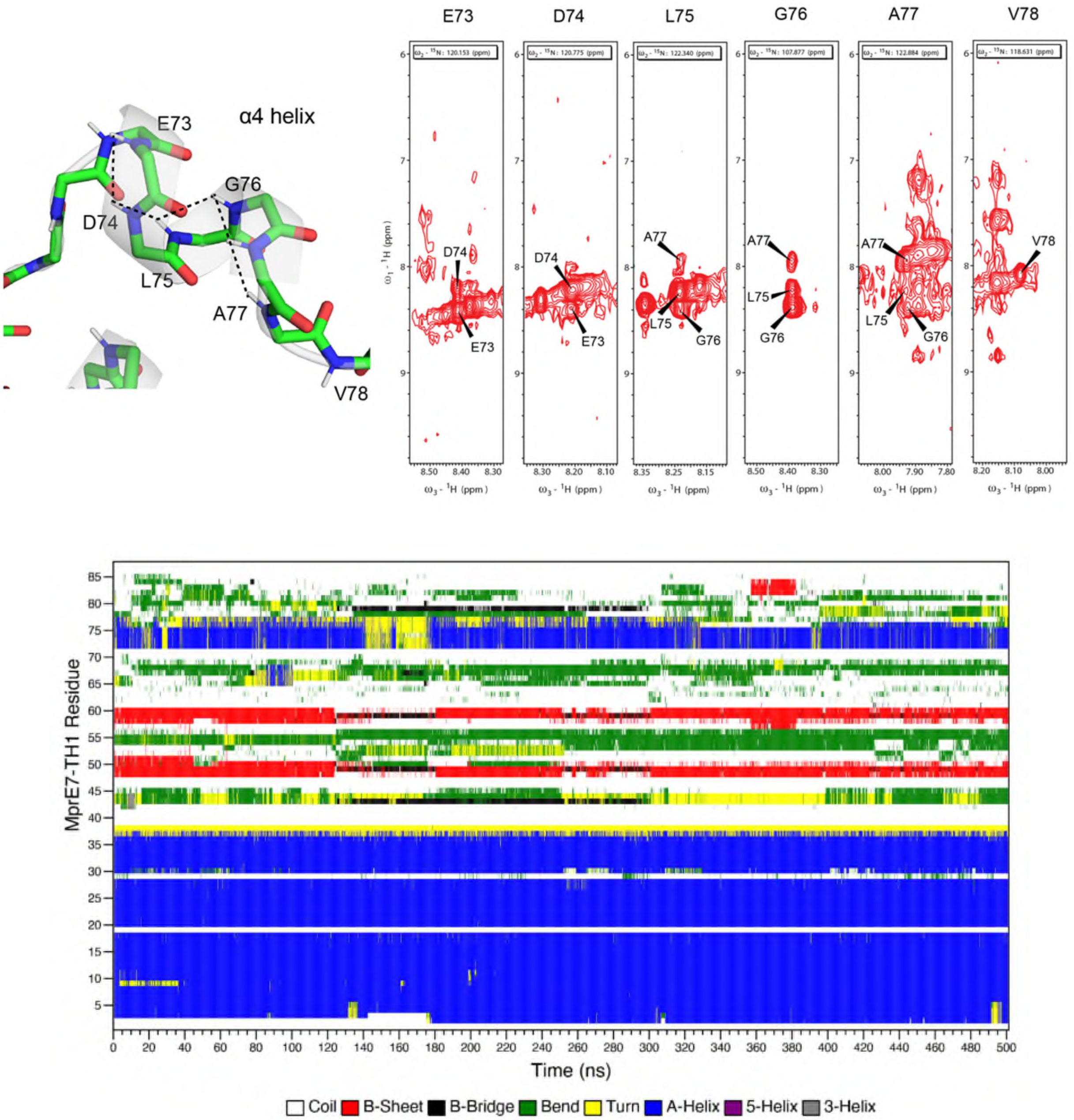

Supplementary Figure 18: Characterization of secondary structure of the MprE7-TH1 α4 helix (residues 73-77). Top: The left panel shows networks of interactions in the α4 helix (PDB ID 8TB1 – this work). The right panel shows 2D ^{1}H-^{1}H strips from the 3D amide-amide NOESY experiment with annotated NOE cross peaks, in agreement with structural elements of residues 73-77. D74 and L75 peaks overlap in ^{1}H chemical shift so NOEs cannot be discerned. Bottom: Per residue secondary structure analyses of MprE7-TH1 as a function of MD trajectory time performed using DSSP tool of GROMACS.

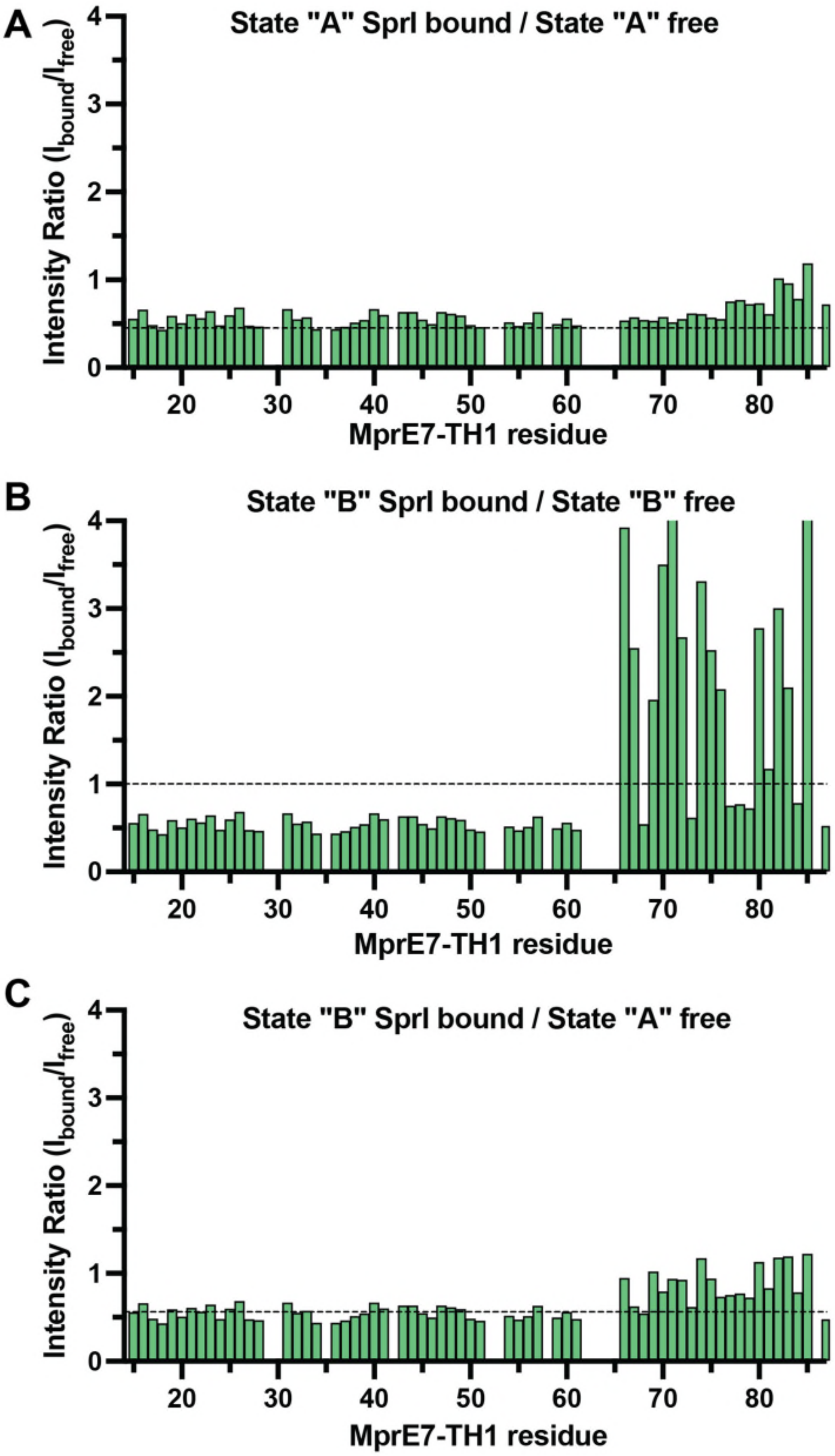

Supplementary Figure 19: Peak intensity ratio analysis of ^{15}N amide probes of the MprE7-TH1/SrpI complex. Peak intensity ratio analysis (I_{bound}/I_{free}) of SrpI bound MprE7-TH1 relative to unbound MprE7-

TH1 was performed at the 1:1 molar ratio of MprE7-TH1/SrpI titration point. At this titration point, MprE7-TH1 and SrpI are both at 42.1 μM. (**A**) Intensity ratios of "state A" peaks in the bound state vs "state A" peaks in the free state for MprE7-TH1. (**B**) Intensity ratio of "state B" peaks in the bound state vs "state B" peaks in the free state for MprE7-TH1. (**C**) Intensity ratio of "state B" peaks in the bound state vs "state A" peaks in the free state for MprE7-TH1. Peaks intensities were not normalized. Black dotted lines in each graph represents one standard deviation below the average I_{bound}/I_{free}.

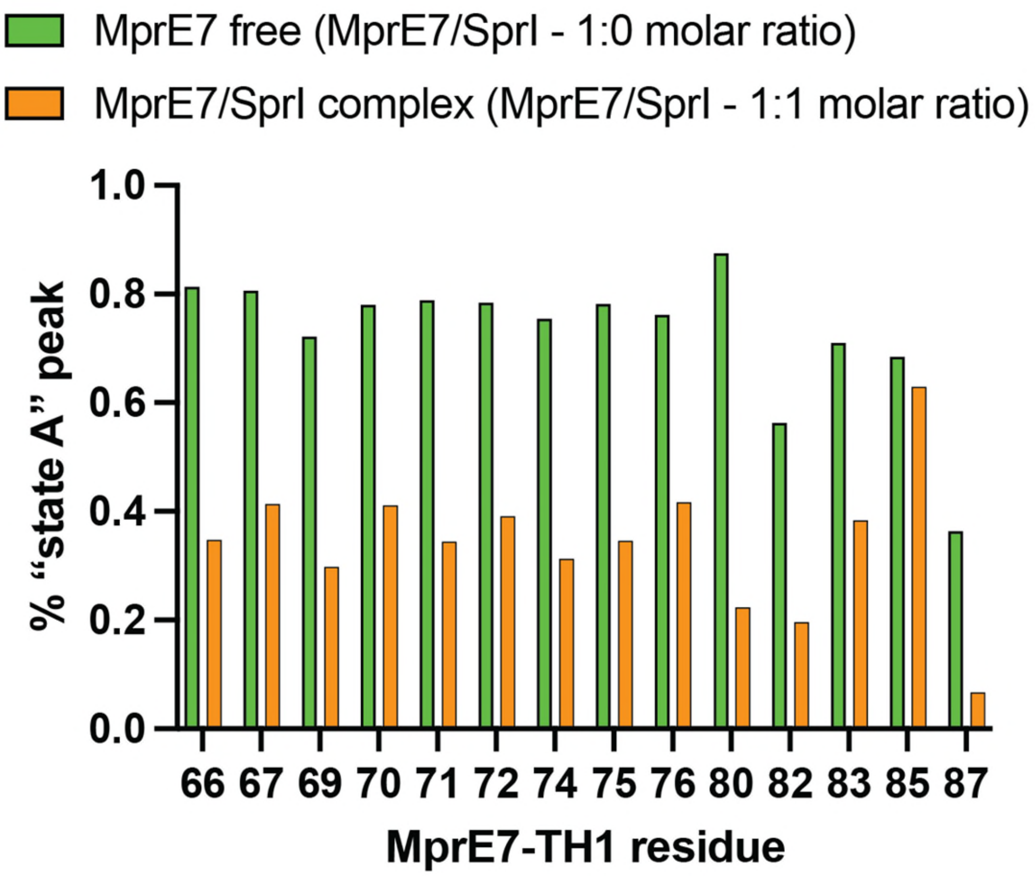

Supplementary Figure 20: Peak intensity analysis of the “state A” conformation of the C-terminal residues of MprE7 leader peptide in the presence and absence of SrpI. In the absence of SrpI (MprE7-TH1 / SrpI molar ratio of 1:0), “state A” NMR peaks corresponding to residues at the C-terminus of MprE7 leader peptide were found to be populated at around 80% relative to “state B” peaks. When SrpI was introduced at equal molar concentration to form the MprE7/SrpI complex, the percentage of “state A” conformation decreased to 20–40% relative to “state B” peaks. Thus, the “state B” conformation becomes more intense in the MprE7/SrpI complex.

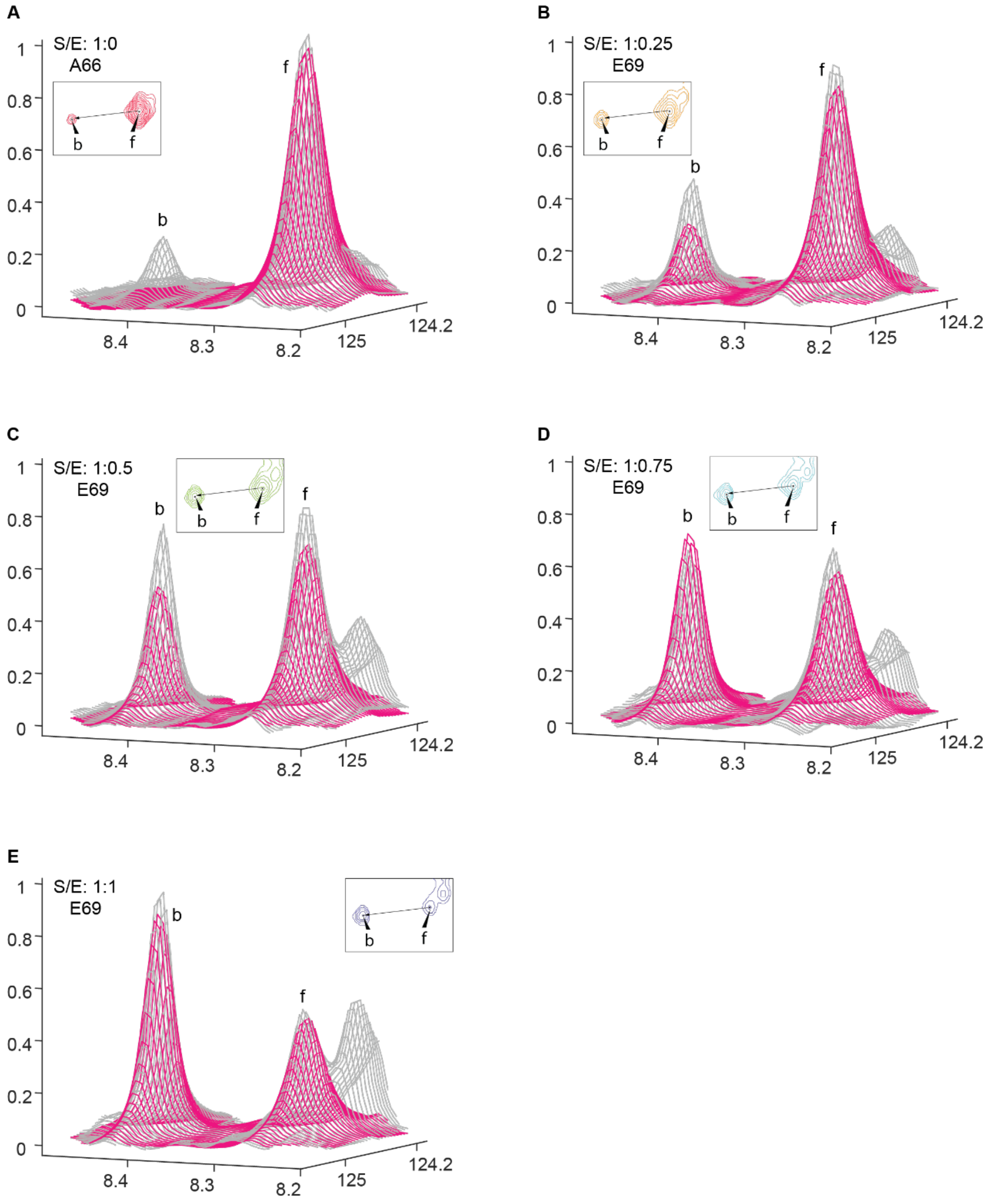

Supplementary Figure 21: Representative titrations of MprE7-TH1 with SrpI. NMR line shape analysis, performed in TITAN, of the cross peak corresponding to residue A66 at different molar ratios of MprE7 to SrpI. The experimental NMR line shapes are colored grey, and the TITAN fits are colored pink (further details in the Materials & Methods section). The concentration of the MprE7-TH1 was kept constant at 42 μM and experiments were recorded at 25ºC at 800 MHz. The NMR peaks of the free (MprE7-TH1) and bound (MprE7-TH1/SrpI) states are noted.

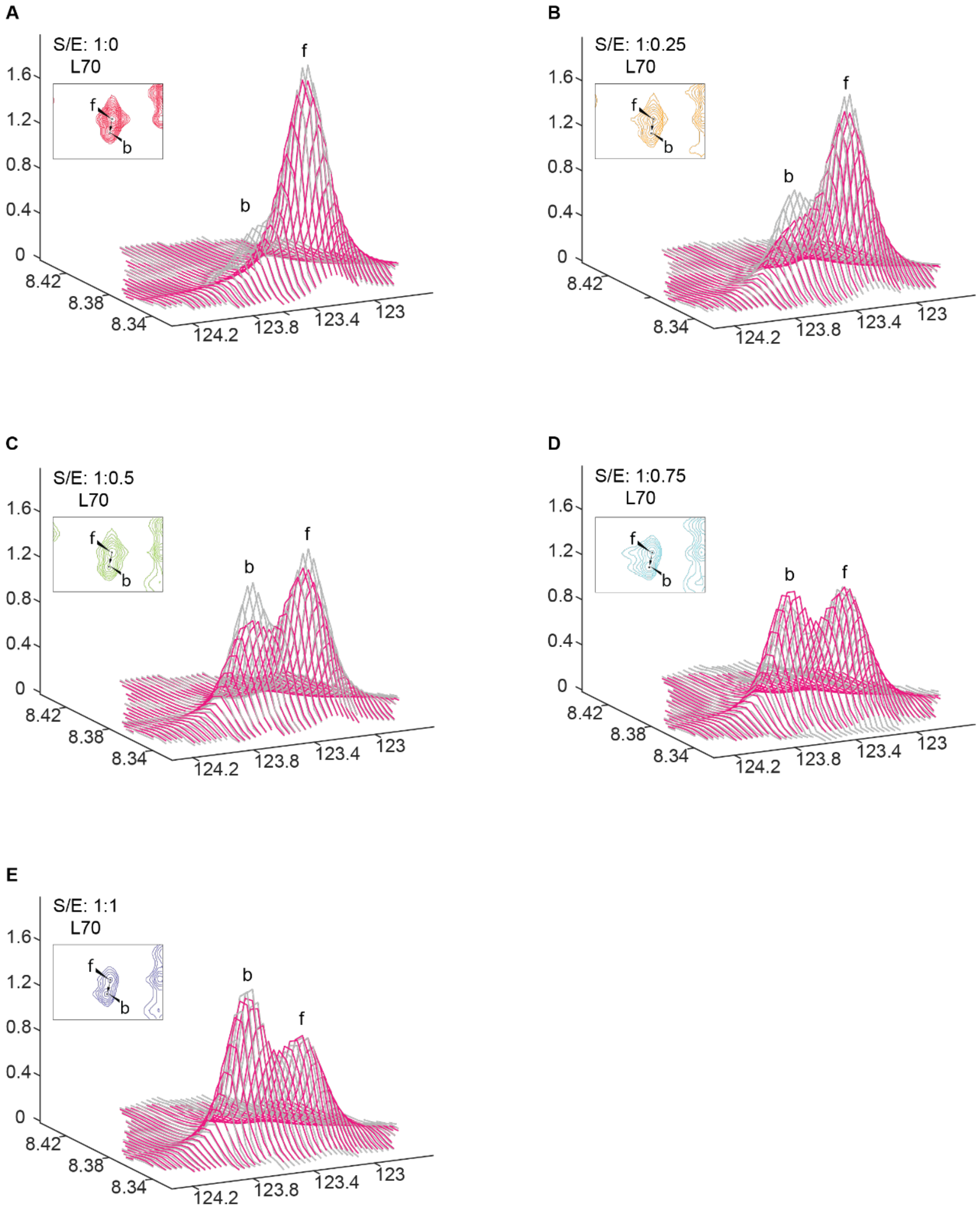

Supplementary Figure 22: Representative titrations of MprE7-TH1 with SrpI. NMR line shape analysis, performed in TITAN, of the cross peak corresponding to residue L70 at different molar ratios of MprE7 to SrpI. The experimental NMR line shapes are colored grey, and the TITAN fits are colored pink (further details in the Materials & Methods section). The concentration of the MprE7-TH1 was kept constant at 42 μM and experiments were recorded at 25ºC at 800 MHz. The NMR peaks of the free (MprE7-TH1) and bound (MprE7-TH1/SrpI) states are noted.

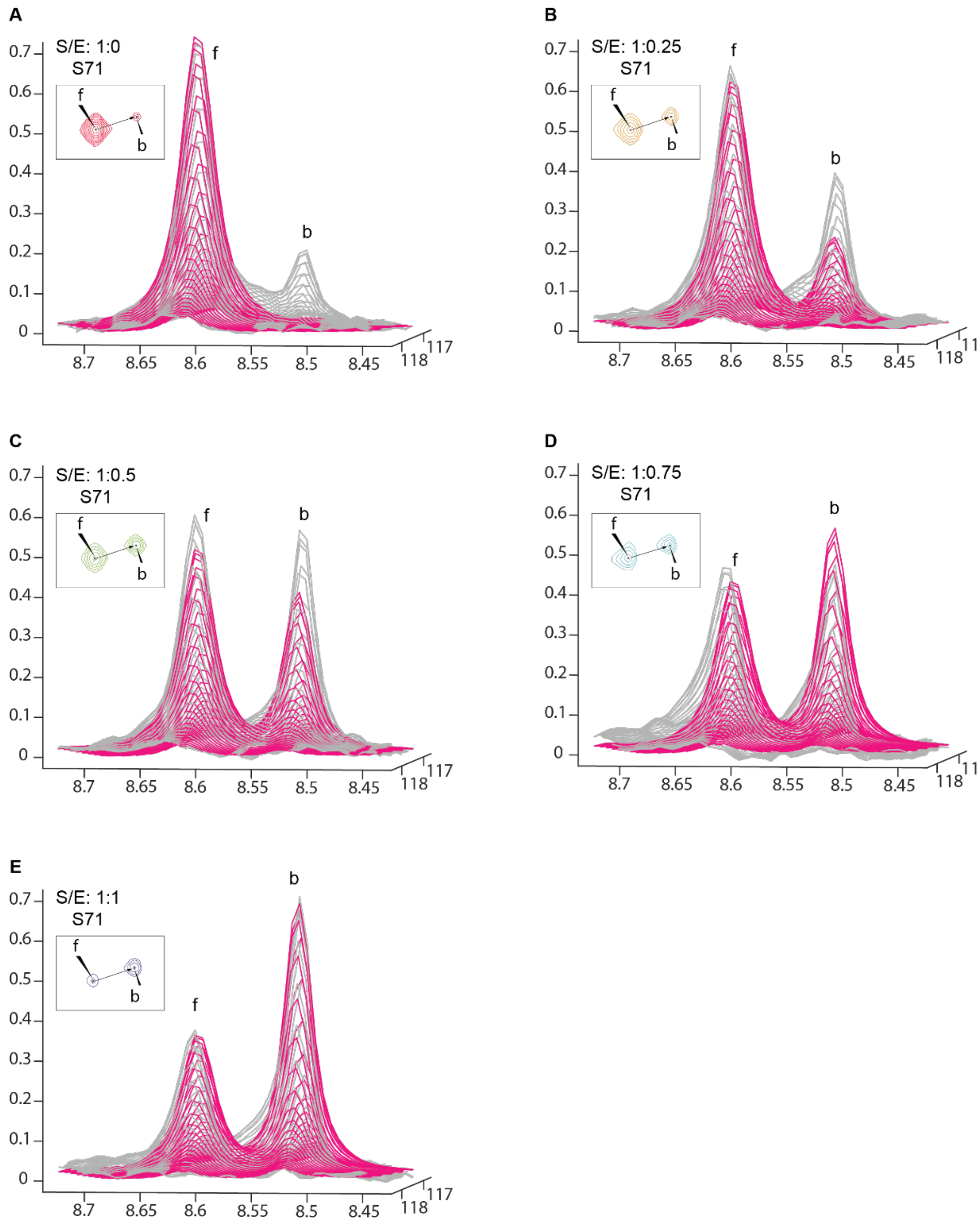

Supplementary Figure 23: Representative titrations of MprE7-TH1 with SrpI. NMR line shape analysis, performed in TITAN, of the cross peak corresponding to residue S71 at different molar ratios of MprE7 to SrpI. The experimental NMR line shapes are colored grey, and the TITAN fits are colored pink (further details in the Materials & Methods section). The concentration of the MprE7-TH1 was kept constant at 42 μM and experiments were recorded at 25ºC at 800 MHz. The NMR peaks of the free (MprE7-TH1) and bound (MprE7-TH1/SrpI) states are noted.

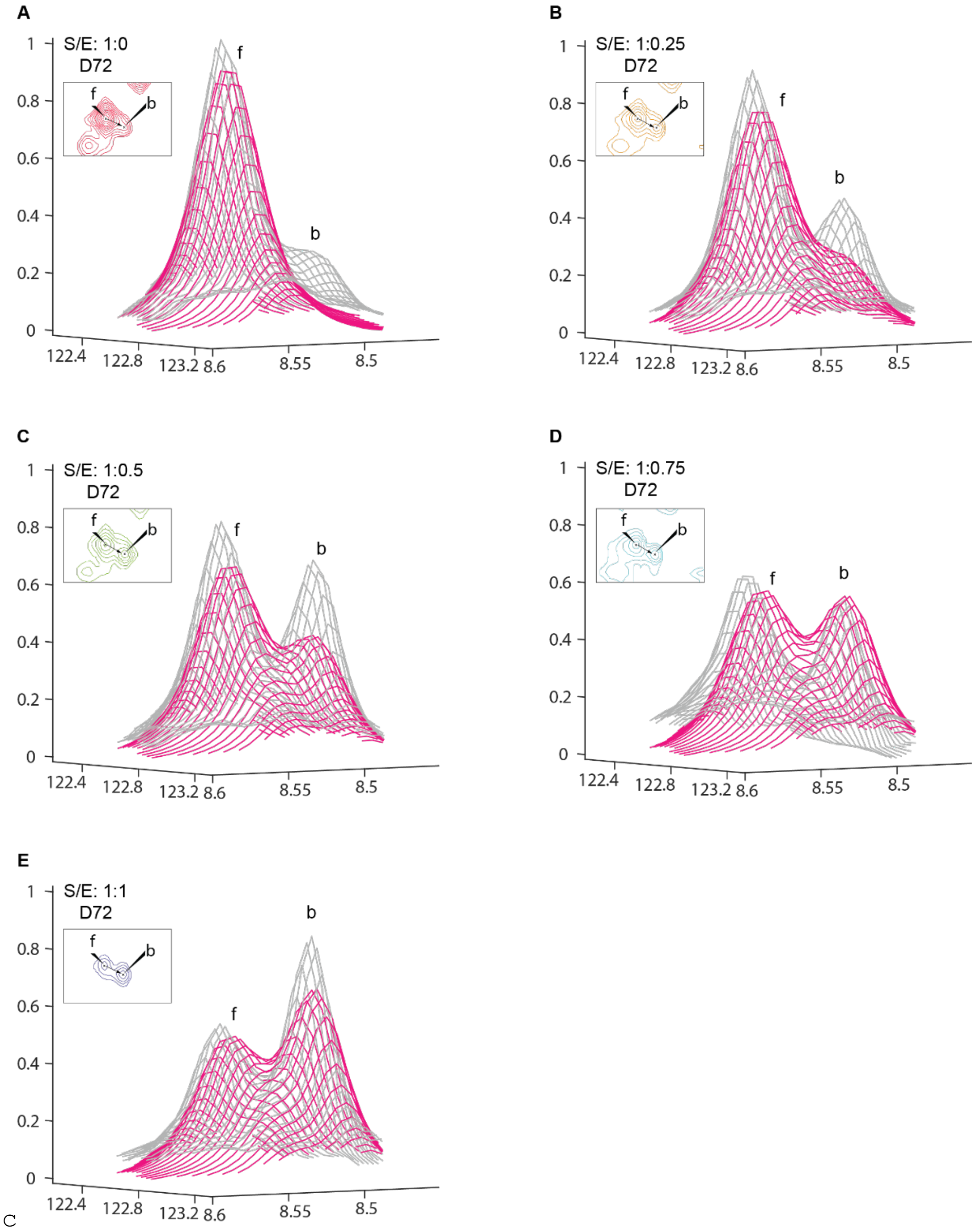

Supplementary Figure 24: Representative titrations of MprE7-TH1 with SrpI. NMR line shape analysis, performed in TITAN, of the cross peak corresponding to residue D72 at different molar ratios of MprE7 to SrpI. The experimental NMR line shapes are colored grey, and the TITAN fits are colored pink (further details in the Materials & Methods section). The concentration of the MprE7-TH1 was kept constant at 42 μM and experiments were recorded at 25ºC at 800 MHz. The NMR peaks of the free (MprE7-TH1) and bound (MprE7-TH1/SrpI) states are noted.

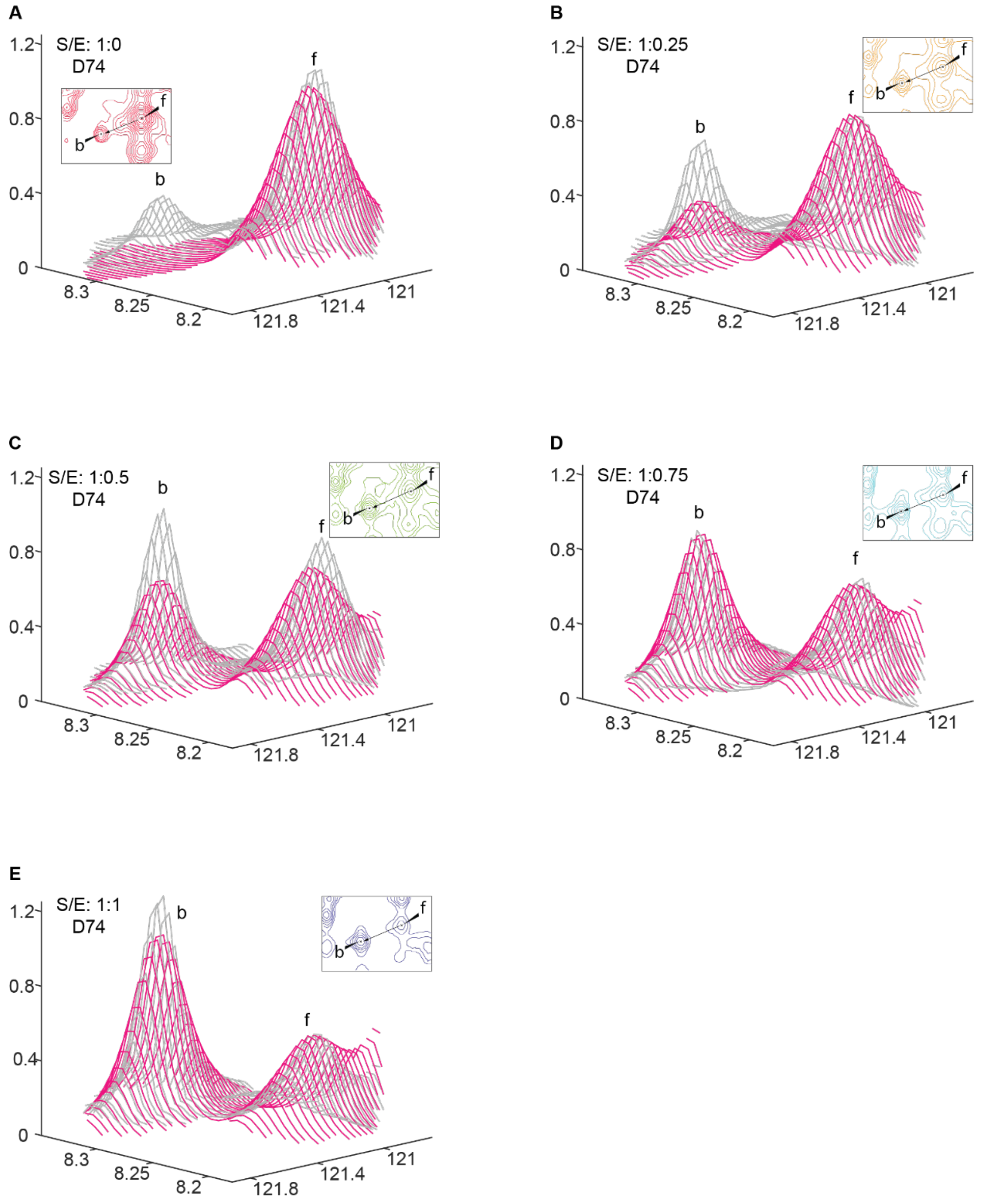

Supplementary Figure 25: Representative titrations of MprE7-TH1 with SrpI. NMR line shape analysis, performed in TITAN, of the cross peak corresponding to residue D74 at different molar ratios of MprE7 to SrpI. The experimental NMR line shapes are colored grey, and the TITAN fits are colored pink (further details in the Materials & Methods section). The concentration of the MprE7-TH1 was kept constant at 42 μM and experiments were recorded at 25ºC at 800 MHz. The NMR peaks of the free (MprE7-TH1) and bound (MprE7-TH1/SrpI) states are noted.

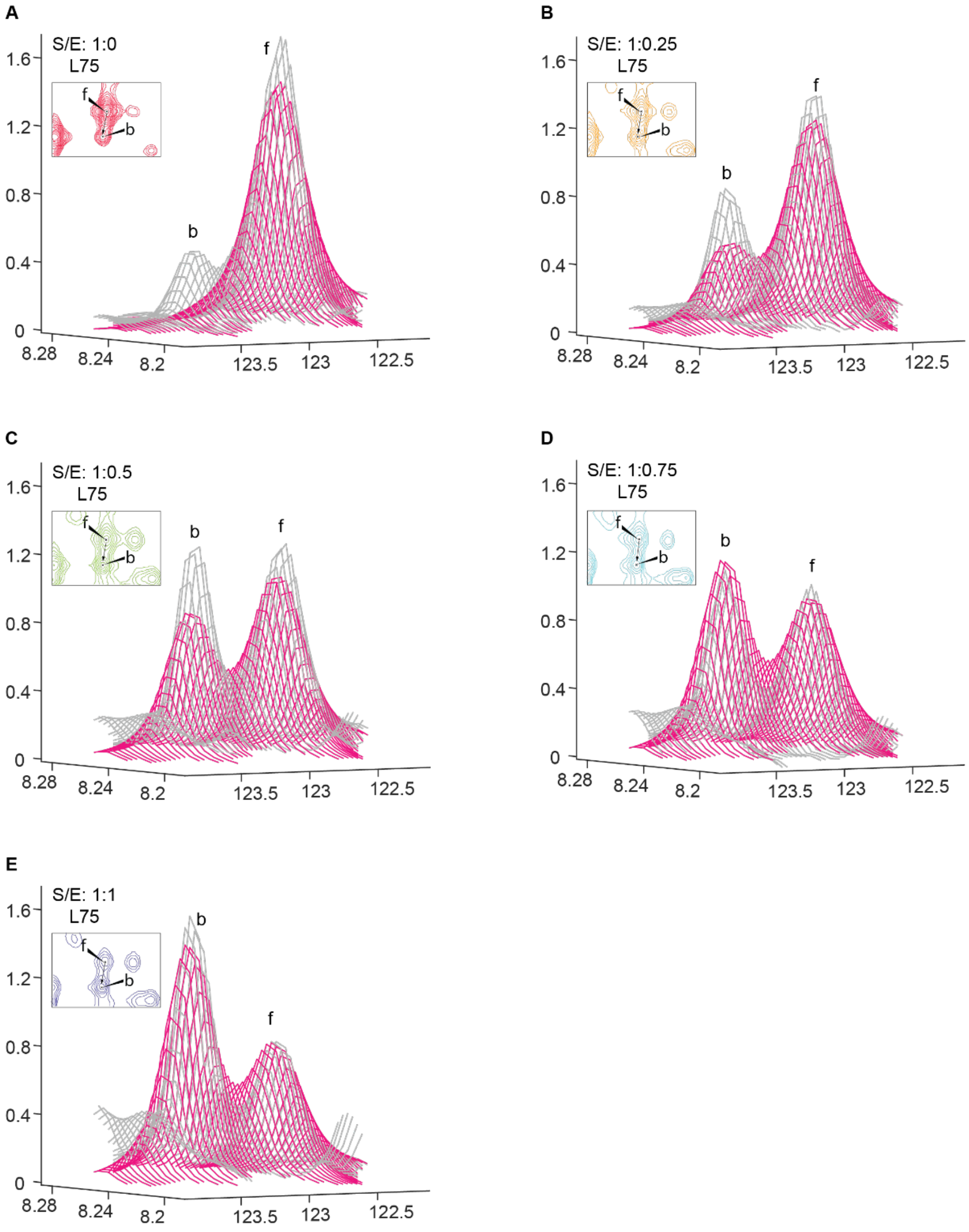

Supplementary Figure 26: Representative titrations of MprE7-TH1 with SrpI. NMR line shape analysis, performed in TITAN, of the cross peak corresponding to residue L75 at different molar ratios of MprE7 to SrpI. The experimental NMR line shapes are colored grey, and the TITAN fits are colored pink (further details in the Materials & Methods section). The concentration of the MprE7-TH1 was kept constant at 42 μM and experiments were recorded at 25°C at 800 MHz. The NMR peaks of the free (MprE7-TH1) and bound (MprE7-TH1/SrpI) states are noted.

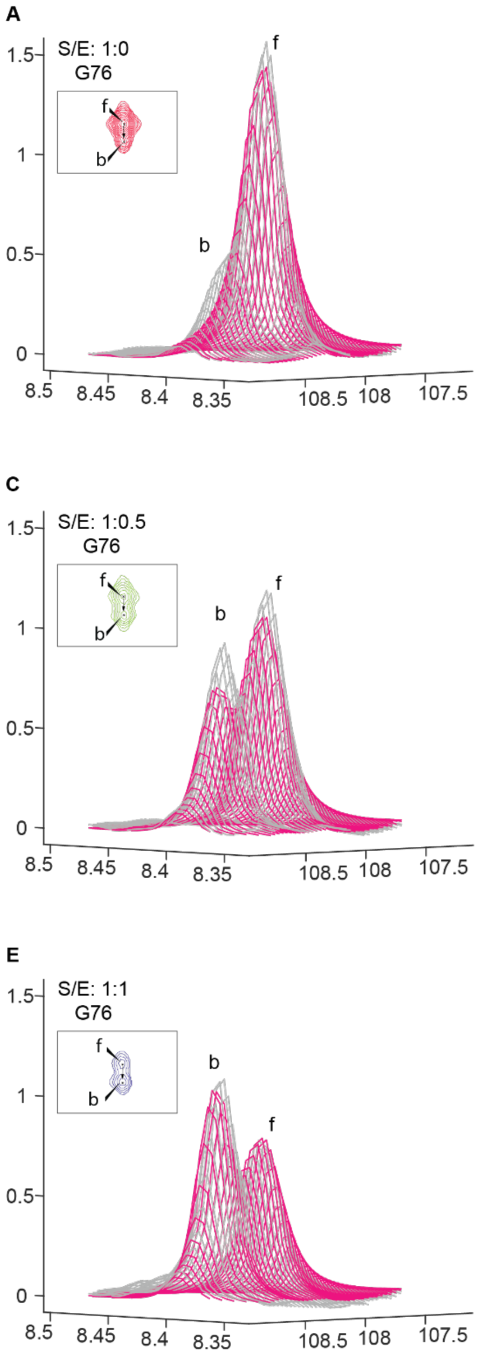

Supplementary Figure 27: Representative titrations of MprE7-TH1 with SrpI. NMR line shape analysis, performed in TITAN, of the cross peak corresponding to residue G76 at different molar ratios of MprE7 to SrpI. The experimental NMR line shapes are colored grey, and the TITAN fits are colored pink (further details in the Materials & Methods section). The concentration of the MprE7-TH1 was kept constant at 42 μM and experiments were recorded at 25ºC at 800 MHz. The NMR peaks of the free (MprE7-TH1) and bound (MprE7-TH1/SrpI) states are noted.

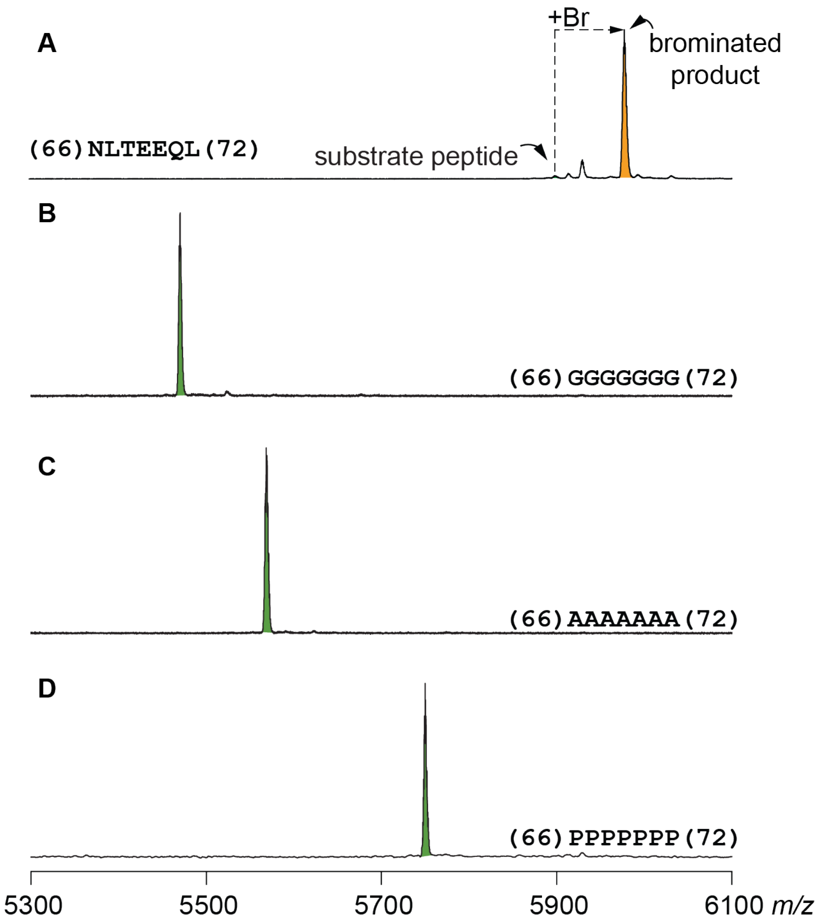

Supplementary Figure 28: MALDI-ToF spectra demonstrate the bromination of (**A**) wild type SrpE-TH1 and the (**B–D**) SrpE-TH1 mutant substrates by SrpI. Peaks corresponding to the substrate peptide are colored in green, and product peaks are colored in orange. Refer to Table S2 for calculated mass and observed mass. Peaks corresponding to the unmodified and brominated substrate are colored in green and yellow, respectively.

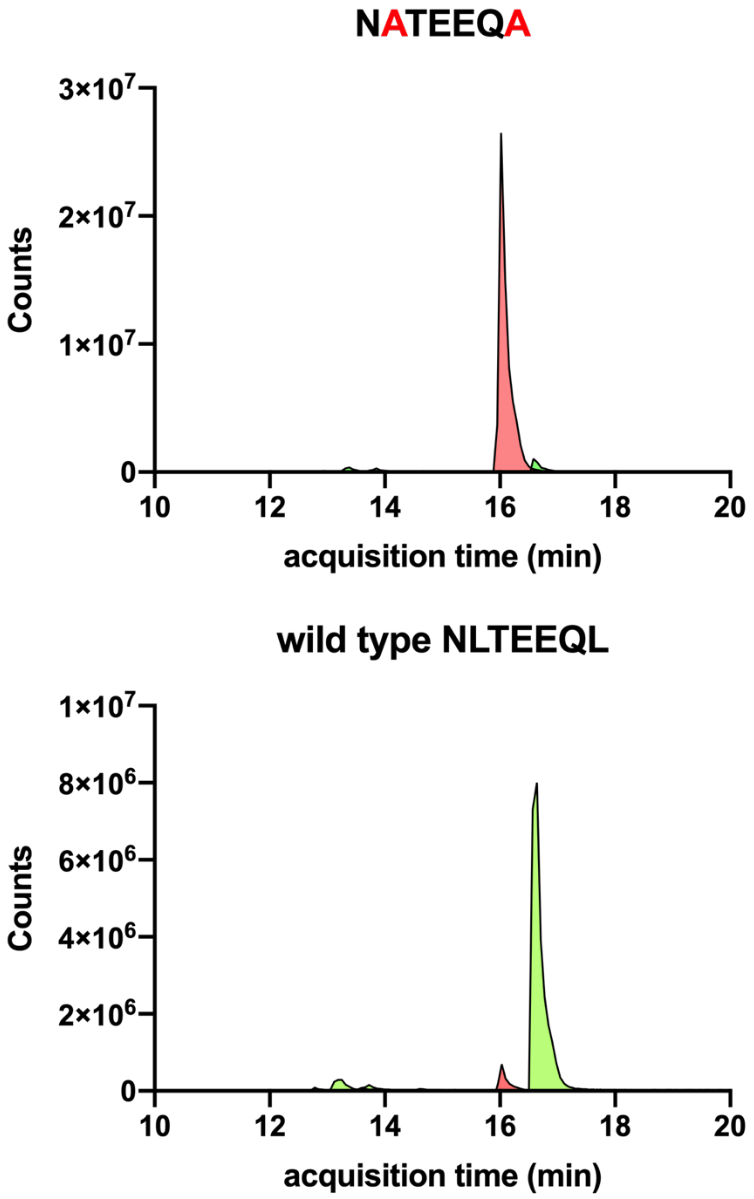

Supplementary Figure 29: LC-MS EICs of the $[M+2H]^{2+}$ ions for the Glu-C digested peptide fragments demonstrating the extent of bromination of **(A)** SrpE-TH1 -NATEEQA- mutant and **(B)** wild type SrpE-TH1 by SrpI. Peaks corresponding to the substrate peptide are colored in red, and product peaks are colored in green. The sites for mutagenesis are highlighted in red. Refer to Table S2 for calculated mass and observed mass.

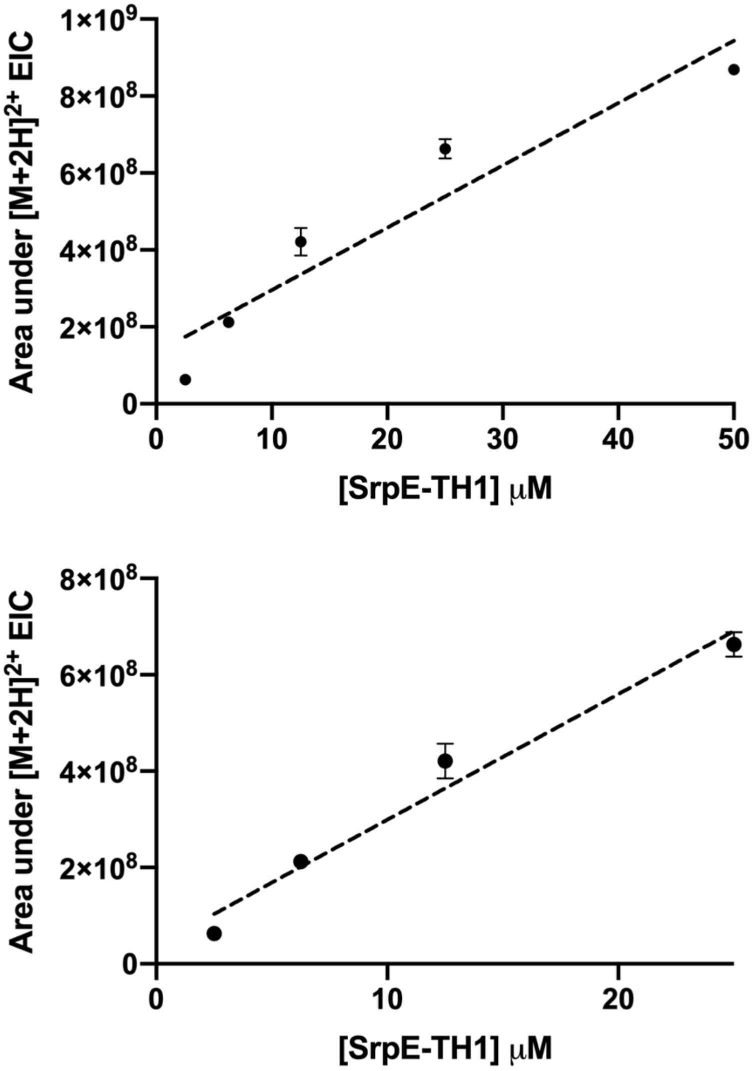

Supplementary Figure 30: Calibration curve for the SrpE-TH1 substrate peptide abundance as detected using LC/MS. The area under the extracted ion chromatograms (EICs) for the $[M+2H]^{2+}$ ion for the Glu-C digested substrate peptide fragment -AISAGLTVLPW- was plotted against protein concentration. Data points represent means from triplicate experiments (n=3), and error bars represent the standard deviations in the measurements. Saturation of the MS detector was observed at 50 μM substrate which compromises the linearity of the fit (top plot). If the 50 μM calibration point is omitted, a better linear fit for the EIC area under the curve vs the peptide concentration is observed. Note that in this study, all enzymatic studies employ 25 μM substrate peptide, which implies that the abundance of the substrate and product peptides will always be less than or equal to 25 μM which falls within the linear detection range for the mass spectrometer (bottom plot).

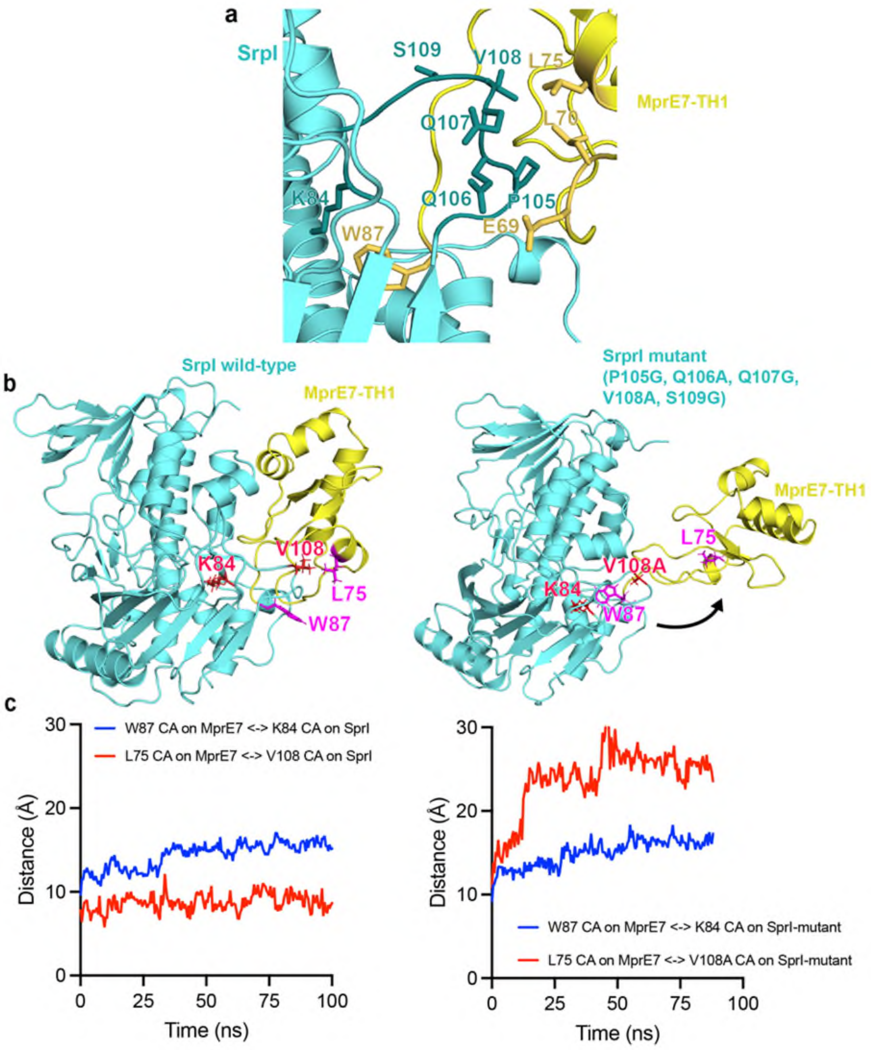

Supplementary Figure 31: MD evaluation of HADDOCK derived SrpI/MprE7-TH1 model **(A)** Zoomed in view of the HADDOCK derived SrpI/MprE7-TH1 interaction interface. Note that W87 of MprE7-TH1 is oriented towards K84 of SrpI. SrpI and MprE7-TH1 are shown as cyan and yellow cartoons, respectively. Amino acid residues at the interface with the SrpI 104-110 loop and the MprE7-TH1 leader are shown as sticks. **(B)** Comparison of the last frame of a 100 nsec MD trajectory of complexes between SrpI wild-type/MprE7-TH1 (left) and SrpI mutant/MprE7-TH1 (right). The MprE7-TH1 leader begins to move away from SrpI upon mutation of the SrpI 104-110 loop. The SrpI mutant contains the following mutations: P105G, Q106A, Q107G, V108A, S109G. Residues used for distance measurements in panel (C) are highlighted. **(C)** Comparison of distance measurements throughout 100 nsec MD trajectories between Cα atoms of SrpI wild-type (K85, V108) and MprE7-TH1 (L75, W87) (left) or SrpI mutant (K85, V108A) and MprE7-TH1 (L75, W87).

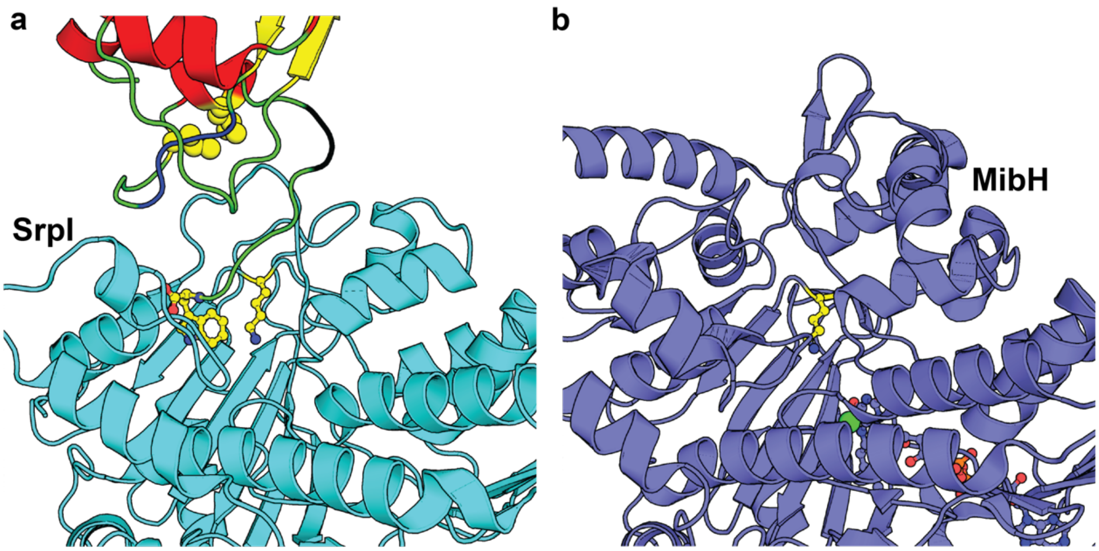

Supplementary Figure 32: Comparison of (**a**) model of the MprE7-TH1/SrpI (this work – NMR/HADDOCK/MD derived model) and (**b**) crystal structure of MibH (PDB: 5UAO). Panel a is illustrated as in Figure 6c. Note that the position and side chain conformation of the catalytic Lys residues of SrpI and MibH (SrpI K84; MibH K102) is superimposable. However, the SrpI surface region where the MprE7-TH1 proteusin peptide binds is conspicuously different than that of MibH. Secondary structural elements present in MibH in this region are absent in SrpI.

Supplementary Tables

Supplementary Table 1: Amino acid sequences of peptides and enzymes used in this study

MBP	MGSSHHHHHH[†]SSGLVPRGS[§]HMKIEEGKLVIWINGDKGYNGLA<br>EVGKKFEKDTGIKVTVEHPDKLEEKFPQVAATGDGPDIIFWAH<br>DRFGGYAQSGLLAEITPDKAFQDKLYPFTWDAVRYNGKLIAYP<br>IAVEALSLIYNKDLLPNPPKTWEEIPALDKELKAKGKSALMFN<br>LQEPYFTWPLIAADGGYAFKYENGKYDIKDVGVDNAGAKAGLT<br>FLVDLIKNKHMNADTDYSIAEAAFNKGETAMTINGPWAWSNID<br>TSKVNYGVTVLPTFKGQPSKPFVGVLSAGINAASPNKELAKEF<br>LENYLLTDEGLEAVNKDKPLGAVALKSYEEELAKDPRIAATME<br>NAQKGEIMPNIPQMSAFWYAVRTAVINAASGRQTVDEALKDAQ<br>TNSSSHHHHHH[†]ANSVPLVPRGS[§]ENLYFQS[‡]GS
MprE7 (leader peptide)	GSHMMNEEQTQQYSQIVAKCWADAEFKAKLIADPKATLAAESI<br>AVPDGIELRVLENTATVVNLVLPPPPAEGELSDEDLGAVT
MprE7-TH1	GSHMMNEEQTQQYSQIVAKCWADAEFKAKLIADPKATLAAESI<br>AVPDGIELRVLENTATVVNLVLPPPPAEGELSDEDLGAVTGG<br>LTVLPW[*]
MBP-SrpE-TH1	*MBP*-MRSGDDMLQHLVEKSALDADFRQQLLADPKSTISQELGISI<br>PESMTIRVHESDMETVHLALPPDPNLTEEQLEAISAGLTVLPW[*]
MBP-SrpE-TH1-G38	*MBP*-GISIPESMTIRVHESDMETVHLALPPDPNLTEEQLEAISAG<br>LTVLPW[*]
MBP-SrpE-TH1-L61	*MBP*-LPPDPNLTEEQLEAISAGLTVLPW[*]
MBP-SrpE-TH1-L67	*MBP*-LTEEQLEAISAGLTVLPW[*]
MBP-SrpE-TH1-NTQ	*MBP*-MRSGDDMLQHLVEKSALDADFRQQLLADPKSTISQELGISI<br>PESMTIRVHESDMETVHLALPPDPALAEEALEAISAGLTVLPW[*]
MBP-SrpE-TH1-LL	*MBP*-MRSGDDMLQHLVEKSALDADFRQQLLADPKSTISQELGISI<br>PESMTIRVHESDMETVHLALPPDPNATEEQAEAISAGLTVLPW[*]
MBP-SrpE-TH1-EE	*MBP*-MRSGDDMLQHLVEKSALDADFRQQLLADPKSTISQELGISI<br>PESMTIRVHESDMETVHLALPPDPNLTAAQLEAISAGLTVLPW[*]
MBP-SrpE-TH1-EEQL	*MBP*-MRSGDDMLQHLVEKSALDADFRQQLLADPKSTISQELGISI<br>PESMTIRVHESDMETVHLALPPDPNLTAAAAEAISAGLTVLPW[*]
MBP-SrpE-TH1-N66A	*MBP*-MRSGDDMLQHLVEKSALDADFRQQLLADPKSTISQELGISI<br>PESMTIRVHESDMETVHLALPPDPALTEEQLEAISAGLTVLPW[*]
MBP-SrpE-TH1-L67A	*MBP*-MRSGDDMLQHLVEKSALDADFRQQLLADPKSTISQELGISI<br>PESMTIRVHESDMETVHLALPPDPNATEEQLEAISAGLTVLPW[*]
MBP-SrpE-TH1-T68A	*MBP*-MRSGDDMLQHLVEKSALDADFRQQLLADPKSTISQELGISI<br>PESMTIRVHESDMETVHLALPPDPNLAEEQLEAISAGLTVLPW[*]
MBP-SrpE-TH1-E69A	*MBP*-MRSGDDMLQHLVEKSALDADFRQQLLADPKSTISQELGISI<br>PESMTIRVHESDMETVHLALPPDPNLTAEQLEAISAGLTVLPW[*]
MBP-SrpE-TH1-E70A	*MBP*-MRSGDDMLQHLVEKSALDADFRQQLLADPKSTISQELGISI<br>PESMTIRVHESDMETVHLALPPDPNLTEAQLEAISAGLTVLPW[*]
MBP-SrpE-TH1-Q71A	*MBP*-MRSGDDMLQHLVEKSALDADFRQQLLADPKSTISQELGISI<br>PESMTIRVHESDMETVHLALPPDPNLTEEALEAISAGLTVLPW[*]
MBP-SrpE-TH1-L72A	*MBP*-MRSGDDMLQHLVEKSALDADFRQQLLADPKSTISQELGISI<br>PESMTIRVHESDMETVHLALPPDPNLTEEQAEAISAGLTVLPW[*]

[†]His_6 tag, [§]thrombin cleavage site, [‡]TEV cleavage site, [*]TH1 core peptide

Supplementary Table 2: Calculated and observed masses of substrate and product peptides in this study.

Compound	Calculated mass	Observed mass
MBP-SrpE-TH1 + LysC	5973.868	5896.287
MBP-SrpE-TH1-**Br** + LysC	5852.925	5975.869
MprE7-TH1 + LysC	5690.010	5690.197
MprE7-TH1-**Br** + LysC	5768.907	5768.986
^{15}N- MprE7-TH1 + LysC	5752.010	5751.958
^{15}N, ^{13}C-MprE7-TH1 + LysC	6007.010	6000.166
MBP-SrpE-TH1-G38 + TEV protease	5367.676	5368.582
MBP-SrpE-TH1-L61 + TEV protease	2834.446	2834.433
MBP-SrpE-TH1-L67 + TEV protease	2201.134	2201.1336
MBP-SrpE-TH1-NTQ + LysC	5764.934	5766.069
MBP-SrpE-TH1-NTQ-**Br** + LysC	5843.83	5844.798
MBP-SrpE-TH1-LL	5810.878	5812.912
MBP-SrpE-TH1-EE + LysC	5778.961	5781.129
MBP-SrpE-TH1-EE-**Br** + LysC	5857.857	5860.004
MBP-SrpE-TH1-EEQL	5679.893	5681.869
MBP-SrpE-TH1-N66A + LysC	5851.966	5852.237
MBP-SrpE-TH1-N66A-**Br** + LysC	5930.862	5930.203
MBP-SrpE-TH1-L67A + LysC	5852.925	5854.567
MBP-SrpE-TH1-L67A-**Br** + LysC	5931.821	5932.939
MBP-SrpE-TH1-T68A + LysC	5864.961	5864.823
MBP-SrpE-TH1-T68A-**Br** + LysC	5943.858	5943.640
MBP-SrpE-TH1-E69A + LysC	5836.966	5836.450
MBP-SrpE-TH1-E69A-**Br** + LysC	5915.863	5915.330
MBP-SrpE-TH1-E70A + LysC	5836.966	5836.314
MBP-SrpE-TH1-E70A-**Br** + LysC	5915.863	5915.129
MBP-SrpE-TH1-Q71A + LysC	5837.95	5837.442
MBP-SrpE-TH1-Q71A-**Br** + LysC	5916.847	5916.275
MBP-SrpE-TH1-L72A + LysC	5852.925	5852.504
MBP-SrpE-TH1-L72A-**Br** + LysC	5931.821	5932.014
AISAGLTVLPW (TH1)	1127.65 $[M+H]^+$<br>564.33 $[M+2H]^{2+}$	1127.58 $[M+H]^+$<br>564.37 $[M+2H]^{2+}$
AISAGLTVLPW-**Br** (TH1-Br)	1205.55 $[M+H]^+$<br>1207.55 $[M+H]^+$<br>603.28 $[M+2H]^{2+}$<br>604.29 $[M+2H]^{2+}$	1205.45 $[M+H]^+$<br>1207.46 $[M+H]^+$<br>604.25 $[M+2H]^{2+}$<br>603.23 $[M+2H]^{2+}$

Supplementary Table 3: SAXS data acquisition, sample details, data analysis, modelling fitting and software used; the shell radii of gyration (Rg) of 17.7Å calculated for the best fitting NMR model agreed with the SAXS derived Rg values tabulated below.

(*a*) Sample details			
Organism: MprE7-TH1 proteusin peptide is a non-natural, artificially designed peptide			
Source: MprE7-TH1 recombinant proteusin peptide expressed in *Escherichia coli*			
Description - sequence (including tags) + bound ligands/modifications, etc.:			
GSHMNEEQTQQYSQIVAKCWADAEFKAKLIADPKATLAAESIAVPDGIELRVLENTATVVNLVLPPPP AEGELSDEDLGAVTGGLTVLPW			
M from chemical composition: 9,541 Da			
Concentration (range/values) measured and method: 523–448 μM			
Solvent details; 20 mM sodium phosphate (pH7.5), 100 mM NaCl and 48 μM FAD			
(*b*) SAXS data collection parameters			
Source, instrument and description or reference	Rotating anode X-ray generator, Rigaku BioSAXS^{nano}		
Wavelength (Å)	1.5418		
Beam geometry (size, sample – detector distance)	100micron, 495.6mm		
q-measurement range ($Å^{-1}$)	0.01-0.07		
Method for monitoring radiation damage	Six ten-minute images overlaid		
Exposure time, number of exposures	10 minutes, 6		
Sample configuration including path length and flow rate where relevant.	100micron quartz capillary		
Sample temperature	Room temperature		
(*c*) Software employed for SAXS data reduction, analysis and interpretation			
SAXS data reduction to (sample – solvent)	Rigaku SAXSlab		
Basic analyses: Guinier, *P*(*r*), Porod volume, volume of correlation.	ATSAS		
Shape/bead modelling	DENSS		
Atomic structure modelling (homology, rigid body, ensemble).	WAXSiS		
3D graphic model representations	PYMOL		
(*d*) Structural parameters			
	523 μM	486 μM	448 μM
Guinier Analysis			
I(0)	8.44	7.98	7.49
R_g (Å)	18.2	18.4	18.5

qR_g range		0.8-1.3	
$P(r)$ analysis			
I(0)	8.55	8.05	7.6
R_g (Å)	18.6	18.6	18.8
d_{max} (Å)	54.7	54.8	58.4
q range	.01–.43	.01–.43	0.01–.42

(*e*) Shape modelling results (DENSS)	
	523 µM
q range for fitting	0.01-0.43
Symmetry/anisotropy assumptions	None
χ^2 range value/range, other quality of fit parameters	1.338
Model volume and/or M_r estimate	40016
Model resolution (Å)	33.1

(*f*) Atomistic modelling (WAXSiS)	
	523 µM
Number of q points for spherical averaging	500
Symmetry assumptions	None
Non-weighted Fit Quality	0.419247
Simulation time, ps	176
Fit method	MD

(*g*) Data and model deposition IDs	SASDTM5

Supplementary Table 4: Minimal media recipe for ^{15}N and ^{13}C labeled peptide production.

Component	Concentration
HCl	1.92 mM
$FeCl_2•4H_2O$	0.1 mg/mL
$CaCl_2•2H_2O$	3.68 µg/mL
H_3BO_3	1.28 µg/mL
$MnCl_2•4H_2O$	0.8 µg/mL
$CoCl_2•6H_2O$	0.36 µg/mL
$CuCl_2•2H_2O$	0.08 µg/mL
$ZnCl_2$	6.8 µg/mL
$NaMoO_4•2H_2O$	12.1 µg/mL
biotin	0.011 µg/mL
folic acid	0.011 µg/mL
p-aminobenzoic	1.1 µg/mL
riboflavin	1.1 µg/mL
pantothenic acid	2.2 µg/mL
pyridoxine	2.2 µg/mL
thiamine	2.2 µg/mL
niacinamide	2.2 µg/mL
KH_2PO4	1.32 mg/mL
K_2HPO4	7 mg/mL
NaCl	1.46 mg/mL
$MgCl_2•6H_2O$	5.6 mg/mL
K_2SO_4	0.048 mg/mL
Thiamine	1 µg/mL
$^{15}NH_4Cl$	1 g/L
$U^{1-}C$ - glucose	3 g/L

Supplementary Table 5: NMR and refinement statistics for MprE7-TH1.

	MrpE7-TH1 (PDB 8TB1)
**NMR distance and dihedral constraints**	
Distance constraints	
Total NOEs	75
Intra-residue	0
Inter-residue	75
Sequential ($\|i-j\| = 1$)	39
Medium-range ($\|i-j\| < 4$)	33
Long-range ($\|i-j\| > 5$)	3
Intermolecular	0
Hydrogen bonds	0
Total dihedral angle restraints	122
Total RDC restraints	59
Total chemical shift restraints	293
**Structure statistics**	
Violations	
Distance constraints (Å)	1.3 ± 0.5
Dihedral angle constraints (º)	0
Max. dihedral angle violation (º)	0
Max. distance constraint violation (Å)	4
Deviations from idealized geometry	
Bond lengths (Å)	0
Bond angles (º)	0
Impropers (º)	0
Average pairwise backbone r.m.s. deviation (Å)	1.5
**Ramachandran analysis**	
Favored	98 ± 2%
Allowed	2 ± 2%
Disallowed	0 ± 0%

Supplementary Table 6: Stoichiometries of NMR titration experiments.

Ratio	MprE7-TH1 concentration (µM)	SrpI concentration (µM)
1:1	42.1	42.1
1:0.75	42.1	31.6
1:0.5	42.1	21.0
1:0.25	42.1	10.5
1:0	42.1	0.0

Supplementary Table 7: Independent residue fits compared to global residue fits when fitting dissociation constant (K_D) and off-rate (k_{off}) using TITAN (residues used for fitting are marked by 'x').

A66	x		x	x
L70	x	x	x	
S71	x	x		x
D72	x	x	x	
D74	x	x	x	
L75	x	x		x
G76	x		x	x
K_D (μM)	24.85 ± 0.60	21.93 ± 0.66	36.92 ± 1.08	21.98 ± 0.62
k_{off} (s^{-1})	3.71 ± 0.49	2.33 ± 0.54	8.92 ± 0.80	2.36 ± 0.56

Supplementary Methods

MD simulation analysis was performed in GROMACS version 2022.4 using the following commands:

```
# center the protein and output the system
echo "1 0" | gmx trjconv -s step5_1_production.tpr -f step5_1_production.xtc -o centered.xtc -pbc mol -ur compact -center

# backbone rmsd analysis
echo "4 4" | gmx rms -s step5_1_production.tpr -f centered.xtc -o rmsd_backbone.xvg -tu ns
# example rmsd analysis for a specific region (i.e., N terminal domain)
# gmx make_ndx -f step5_1_production.tpr -o core.ndx
# 4 & r 1-59
# q
echo "16 16" | gmx rms -s step5_1_production.tpr -f centered.xtc -o rmsd_bb_all.xvg -tu ns -n core.ndx

# backbone rmsf analysis; skip first 5000 psec
echo "4" | gmx rmsf -s step5_1_production.tpr -f centered.xtc -o rmsf_backbone.xvg -res -b 5000

# secondary structure analysis on mainchain
echo "5" | gmx do_dssp -f centered.xtc -s step5_1_production.tpr -o secondary-structure.xpm -sc secondary-structure.xvg -tu ns

gmx xpm2ps -f secondary-structure.xpm -di ssgraph_parameters.m2p -o secondary-structure.eps

# distance measurement example for L75 CA (atom number 1117) to T6 CA (atom number 83)
echo "1 q" | gmx make_ndx -f step5_1_production.tpr -o step5_1_production.ndx

echo "atomnr 83 1117" > distance_selections
```

```
cat distance_selections | gmx distance -n step5_1_production.ndx -f centered.xtc -s step5_1_production.tpr -oall distances_ca_to_ca.xvg -tu ns -dt 0.5 -len 1.4 -tol 1 -binw 0.01
```

Supplementary References

1. Sreerama, N.; Venyaminov, S. Y.; Woody, R. W., Estimation of protein secondary structure from circular dichroism spectra: inclusion of denatured proteins with native proteins in the analysis. *Analytical Biochemistry* **2000,** *287* (2), 243-251.

2. Kabsch, W.; Sander, C., Dictionary of protein secondary structure: pattern recognition of hydrogen-bonded and geometrical features. *Biopolymers* **1983,** *22* (12), 2577-2637.
