# Supplementary material for: Disordered regions in proteusin peptides guide post-translational modification by a flavin-dependent RiPP brominase
Source: Nat Commun. 2024 Feb 10;15:1265. doi: 10.1038/s41467-024-45593-5 (PMC10858898; doi:10.1038/s41467-024-45593-5)
Supplement: Supplementary file 3 — Description of Additional Supplementary Files [file 41467_2024_45593_MOESM3_ESM.pdf]

Description of Additional Supplementary Files

File Name: Supplementary Movie 1
Description: MD evaluation of HADDOCK derived SrpI/MprE7-TH1 model.
Representative movie taken from 100 nsec trajectory all-atom MD simulation of the NMR/HADDOCK derived complex for MprE7-TH1/SrpI (see also Supplementary Figure 31). MprE7-TH1 is shown in yellow; SrpI is shown in green. The catalytic K84 of SrpI is shown as a blue stick; L75, V108, and W87 of MprE7-TH1 are shown as a yellow stick. The movie was generated by the VMD software.
