# Supplementary material for: Disordered regions in proteusin peptides guide post-translational modification by a flavin-dependent RiPP brominase
Source: Nat Commun. 2024 Feb 10;15:1265. doi: 10.1038/s41467-024-45593-5 (PMC10858898; doi:10.1038/s41467-024-45593-5)
Supplement: Supplementary file 2 — Peer Review File [file 41467_2024_45593_MOESM2_ESM.pdf]

nature portfolio

Peer Review File

Disordered regions in proteusin peptides guide post-translational modification by a flavin-dependent RiPP brominase

Open Access This file is licensed under a Creative Commons Attribution 4.0 International License, which permits use, sharing, adaptation, distribution and reproduction in any medium or format, as long as you give appropriate credit to the original author(s) and the source, provide a link to the Creative Commons license, and indicate if changes were made. In the cases where the authors are anonymous, such as is the case for the reports of anonymous peer reviewers, author attribution should be to 'Anonymous Referee' followed by a clear attribution to the source work. The images or other third party material in this file are included in the article's Creative Commons license, unless indicated otherwise in a credit line to the material. If material is not included in the article's Creative Commons license and your intended use is not permitted by statutory regulation or exceeds the permitted use, you will need to obtain permission directly from the copyright holder. To view a copy of this license, visit http://creativecommons.org/licenses/by/4.0/.

REVIEWER COMMENTS

Reviewer #1 (Remarks to the Author):

The manuscript "Disordered regions in proteusin peptides guide post-translational modification by a flavin-dependent RiPP brominase" by Nguyen et al. describes the first structural characterisation of a larger leader peptide involved in the biosynthesis of ribosomal natural products (RiPPs). In RiPP biosynthesis, the starting material is usually a short, ribosomally produced peptide that contains a leader (enzyme recognition) and a core peptide. The core peptide is ultimately transformed into the natural product. How RiPP enzymes recognise the leader peptides of their respective substrates has thus far only been poorly understood. Yet this area holds a lot of promise to make RiPP enzymes suitable for industrial applications. Since the leader is essentially a tag that leads to processing, it may be considered a waste product and reducing the size of leader peptides is essential to improve the atom economy of RiPP production. Nguyen et al. provide an important piece of the puzzle by elucidating the 3D structure of an unusually large group of leader peptides, those from proteusin pathways, and demonstrate how this knowledge can be used to dramatically shorten the leader peptide without sacrificing too much enzymatic efficiency: Removing 60 amino acids from an 81 amino acid leader peptide only decreased the yield by 13%.

Overall, this manuscript provides very important information and I recommend publication in NatComms once the manuscript has undergone major revisions. I would like to add that I am not an NMR or MD expert and therefore can only say that these sections appear to be done very well, but refer to an expert for final judgement.

My revision requests are largely related to how the data is presented. NatComms requires articles to be written for a general audience, and that is where this manuscript falls short.In my opinion a general audience would be most attracted to the point made by the authors on p3 L74, and the conclusion. Combined with the additional support the authors provide for the importance of the L(X)4L-motif in RiPP biosynthesis the authors provide important reference points for future RiPP engineering efforts and the exploitation of RiPP enzymes for Trp bromination. I would center the story on those areas.
I know that these comments are, from an authors' perspective, more annoying to deal with than purely scientific comments and the authors are free to ignore them. I apologise if I overstepped, I am only trying to help.

Specific:

Figure 1a: Not heterocycles in OspA?

Relating to Figure 2e: The Trp appears to sample different orientations and positions, but have a preferred volume it occupies. Is there an way for the authors to relate these data to enzymatic turnover, as in, is one of those likely to be best for processing?

I would encourage the authors to run additional biochemical reactions for the data shown in Figures 4c and 5 to reduce the size of the errors. It would also be good to provide calibration curves for at least some of the peptides to ensure that responses in MS are linear in the concentration range of the assay.

Figure S16 and accompanying text on page 7, starting line 172: It would be very beneficial for a general audience to expand that section and create a panel or figure to illustrate that point for a general audience.

Could the authors provide NMR evidence that the severely truncated peptide still binds to SrpI with similar affinity and is in fact disordered in solution?

Reviewer #2 (Remarks to the Author):

In the manuscript under review, the authors investigate the structural characteristics of ribosomally synthesized and post-translationally modified peptides (RiPPs) with a specific focus on proteusin peptides. Employing an array of techniques, including solution NMR spectroscopy, SAXS, and molecular dynamics (MD), they elucidate the three-dimensional structure of proteusin peptides and their interaction with flavin-dependent brominase enzymes.
My expertise predominantly lies in SAXS methodology, so my review will chiefly address the data and conclusions emanating from this technique. Overall, the data are robust, and the analysis appears to be rigorous. However, I have several critical points for consideration:
Figure S7: While the figure successfully negates concerns about radiation damage and structure factor, the SAXS profiles exhibit minor shifts in I(0). This issue, also evident in Figures S8 and S9, likely arises from an instrument scaling error and warrants manual correction, possibly through Primus software.
Figure S10: Contrary to the authors' conclusions, the Kratky plots exhibit bell-shaped curves, indicative of structural rigidity rather than flexibility. Neither the P(r) functions nor the small-angle region data sets corroborate the claim of flexibility. I suggest that the authors employ Flory’s equation to explore the correlation between Rg and protein length as a more robust analytical method.
Line 153: The statement that NMR/WAXSiS and DENSS models offer independent verification is misleading. Both models derive from SAXS data, making their concordance expected rather than confirmatory. Furthermore, the utility of the DENSS model in this context appears questionable.
Missing Analyses: I could not locate the CRYSOL and OLIGOMER fits of the top-ten NMR models, crucial elements for a comprehensive SAXS analysis. The inclusion of statistical measures such as Chi^2 or CorMap p-values tests for these fits would enhance the study's robustness. Availability of the data in open databases e.g. in SASBDB would be beneficial and strengthen the rigor and trustworthiness of this study. Consider also employing EOM program from ATSAS designed specifically for analyzing flexible/disordered proteins.
Minor Notes:
Line 143: SAXS should not be classified as a spectroscopy technique.
Line 241 (SI): The X-ray beam energy should likely read 12 keV rather than 1.2 keV.
In summary, the manuscript is scientifically sound and its contributions are valuable. However, several issues need to be addressed before it is ready for publication.

Reviewer #3 (Remarks to the Author):

This work uses various spectroscopic methods (SS-NMR and SAXS) as well as MD simulation to investigate proteusin peptide-brominase interactions. The proteusin has ordered and disordered regions and it is found that the ordered part can be excised without catalytic function loss. The LXXXXL motif is found to be crucial for Bromination as 1) SrpI interaction features the LXXXXL region and 2) XXXX mutation inhibits Bromination. Modelling is done to suggest the nature of the proteusin peptide-Brominase interaction.
The experimental methodology seems sound. But there are issues of interpretation that should be addressed before publication, in my view.

Some issues to address:
1) The three MD trajectories are strange in that they are so different. This is ascribed to "these observations are suggestive of an equilibrium heterogenous mixture of MprE7-TH1 conformations in solution". An MD trajectory in principle would sample in one trajectory all these states. It seems that what is actually seen are three non-equilibrated trajectories.
It could be worthwhile to look at BB RMSD excluding residues ~60-90. Are those three trajectories similar? It might give confidence that it is the C-terminus that is responsible for trajectory differences. Note that it is RMSD (versus RMSF by residue) that would point to 'heterogenous mixture' (see following comment).

2) Continuing in this vein, much of the analysis depends on a conformational selection (versus induced fit) hypothesis: "these observations are suggestive of an equilibrium heterogenous mixture of MprE7-TH1 conformations in solution." That seems rather circumstantial. It means that there are large barriers in free energy separating conformers. While certainly possible, if the unstructured C-terminal region is genuinely unstructured it is hard to see that it samples distinct conformers. Instead of this hypothesis why isn't induced fit just as sensible?

3) It is worth citing some refs for conformational selection versus induced fit; e.g. "Induced fit, conformational selection and independent dynamic segments: an extended view of binding events. Csermely, P; Palotai, R and Nussinov, R", TRENDS IN BIOCHEMICAL SCIENCES 35 (10) , pp.539-546 (2010). (It has ~600 forward refs). And couching the discussion in these terms.

4) While the results of this study are intriguing, what is missing is insight into the reaction mechanism. What chemistry is being carried out in the Bromination? With the geometrical constraints of the wild-type and mutated-residue complexes available, correlated with the reaction kinetics, it should be possible to suggest a reaction mechanism. To me, this is the raison d'être of this kind of study.

Typo: "determined for Pro residues are as"

Reviewer #4 (Remarks to the Author):

In their manuscript Nguyen et al. characterize the structural properties of the unusually long bacterial proteusin RiPP leader peptide and its binding to the brominase SrpI. Since the physiological substrate of SrpI, SrpE, is difficult to assess recombinantly, the authors come up with an elegant strategy, by why they fuse the related MprE7 to the tumor homing peptide-1 (TH1), which can be C-terminally brominated in the absence of further post-translational modification. In a detailed NMR study, the authors discover, that a major part of the proteusin leader peptide is structured and they determine its solution structure using NMR spectroscopy and SAXS. They identify that the C-terminal part of the peptide is intrinsically disordered and that this part also presents the essential interaction site with SrpI and characterize SrpI activity as a function of binding site mutations using mass spectrometry. In their docking and MD-derived model of MprE7-TH1 binding to SrpI, the authors explain the importance of the identified LXXXXL peptide for the correct positioning of the C-terminus in the catalytic pocket and compare this model to binding of other known LXXXXL motifs involved in RiPPs.

The study is extensive, using many different techniques and providing new insights into the function of the long proteusin leader peptide. However, several points need to be addressed:

Firstly, this concerns the observation of two sets of NMR peaks at the C-terminal intrinsically disordered peptide of MprE7. One set is populated to 80% in the unbound state. The minor state (populated to 20% when unbound) resembles the bound state, and increases in population as the concentration of SrpI increases. This interesting observation appears in the manuscript far too late and then opens a couple of questions as to how previous data have been analyzed (e.g. secondary structure content, chemical shift perturbations, etc…). Additionally, mentioning the population of the minor state earlier would avoid confusion concerning figures that are cited earlier in the text, for instance the insets in Figure 4. The authors should revisit this critical point and restructure the results section of their manuscript accordingly.

Secondly, the authors seem to identify that, despite the presence of a folded domain at the N-terminus of the analyzed MprE7 leader, the C-terminal unstructured region is the central interacting region for SrpI and activity measurements convincingly demonstrate that the folded part is dispensable. This seems to partially conflict with the last phrase of the abstract that 'RiPP modification enzymes [...] not only [use] small unstructured precursor peptides, but also structured proteins as substrates'. Along these lines and maybe in context with their model of the MprE7-TH1/SrpI complex, it would be interesting if the authors could come up with an idea why the folded part exists and what role it might play (or not) in correctly positioning the LXXXXL peptide.

Additionally, the following specific points below will need to be addressed:

- The authors state that they assigned 72.5% of the chemical shifts. Can they explain why the remaining resonances were not/could not be assigned?
- Can the authors be more precise (e.g. in the methods section), which data were used in the structure calculation, e.g. a list of constraints that were used, how these were fulfilled etc.
- Which RDCs were used in the structure calculation? Could the authors plot the RDCs from Figure 2d

(experimental and predicted) along the protein sequence? Is the quality of agreement similar between the structured regions and the disordered region?
• The authors should explain briefly what the 'NMR/WAXSiS refined' model is (line 153) also in the main text (not only in the figure legend). Could the authors provide any means to judge the goodness of the fit into the SAXS-derived solvent envelope (Figure 2f)?
• The authors state that, C-terminal from beta2, the remainder of the leader peptide is disordered apart from helix alpha4. Can the authors detail, based on which data they draw this conclusion. In Figure 2c, alpha4 does not seem to be indicated in the SSI (while the RCI-S2 increases around this position). What is the reason for this? Can the authors also indicate (and show) based on which data they conclude that there are 'minimal contacts' (line 167) between alpha4 and the N-terminus of the leader peptide?
• Figure 3d: Could the authors provide two structures with two different (one long, one short) distances between L75 and T6 and annotate the actual distances onto the structures? This would help appreciate the movement.
• In lines 207-214, the authors discuss NMR line broadening of MprE7-TH1 spectra in complex with SrpI. They first suggest 'intermediate exchange NMR line broadening', then that the line broadening is 'likely due to rapid transverse spin relaxation in the ~60 kDa […] complex.' As no data on the exchange behavior of the complex exists, the authors should be more open in their interpretation, speak about line broadening in a more general sense and only then discuss the different possibilities why this line broadening may occur.
• Figure S18 shows an intensity ration between the spectra of MprE7-TH1 in complex with SrpI and alone, whereby no region seems to show specific line broadening, as the authors also mention in the main text. Can the authors state at which concentrations these intensity ratios were acquired and, whether they used the bound or free state peaks for calculating the intensity ratios? The label of the x-axis should be bigger.
• The authors observe a minor state in the C-terminal disordered part of the leader sequence, which gets more populated as the concentration of SrpI increases. In view of this observation: Which peaks were used to generate the CSPs and intensity ratios (see also previous point)? Which peaks were used to calculate the SSI and RCI-S2 and what would using the respective other peak yield? Judging from the assignment strip plot, the carbon chemical shifts of the bound and the unbound states are the same. However, it would be interesting to know if this is indeed true. The chemical shift of the carbon dimension is not annotated on the plots (Figures S11-S14)
• Based on previous data on OspA, the authors speculate that the N-terminal folded region might be important for the overall conformation of the proteusin peptide (and thus the disordered C-terminus). This could easily be tested for MprE7-TH1 using only the C-terminal disordered region. Would the authors be able to provide these data?

Minor points:
• Figure 1e: Why is the leader peptide brown and not blue as in the other sub-figures? This should be changed or explained in the legend.
Somehow, Figure 1e does not seem to fit to the other sub-figures, since it describes a reaction, not a mechanism and indeed, the authors state that SrpI follows the model from Figure 1c. Maybe the authors could find a way to set Figure 1e apart from the rest of the figure.
• Lines 85 and 86: 'in the SrpE leader than has been…' should be changed to '...that has been…' and

‘withs’ to ‘with’. The sentence also seems to be lacking a citation.

- Lines 94/95: ‘Gratifyingly, leader sequences from other proteusin peptides, MprE7 and OspA, supported SrpI activity (Fig. 2a).’ The reference to Figure 2a seems somewhat misplaced, since it shows sequence alignments rather than SrpI activity.
- Line 95: ‘Of these, gene…’, please insert ‘a’: ‘Of these, a gene…’
- Figure S5 would benefit from a more detailed legend. What are the different secondary structure elements that are considered in the fit? Which are beta-sheets, which are disordered for example, as indicated in the main text (lines 99/100)?
- Figure S8: There seem to be points in a different red/cyan/blue under the circles with a darker border. It does not become clear from the figure legend what this might be (raw data and Guinier fit?). The authors should clarify this and maybe choose a line or so for fitting (something that is distinct from the raw data).
- U-[15N/13C] should be explained.
- A citation for the random coil index seems to be missing (line 147).
- Figure 2e: It would be helpful to have a color legend for this figure.
- Figure 4: The authors should explain how they analyzed the time dependent SrpI activity in sub-figures c to f briefly in the figure legend (especially as the rest of the figure is focused on NMR experiments); a few language errors need to be corrected in the figure legend.
- A legend for the movie seems to be missing.

We thank the reviewers for their thoughtful comments and critique, addressing which has undoubtedly made the manuscript stronger and more accessible to a general audience. We reproduce the reviewers' comments verbatim below and provide a point-by-point response in *dedicated font*.

Reviewer #1 (Remarks to the Author):

The manuscript "Disordered regions in proteusin peptides guide post-translational modification by a flavin-dependent RiPP brominase" by Nguyen et al. describes the first structural characterisation of a larger leader peptide involved in the biosynthesis of ribosomal natural products (RiPPs). In RiPP biosynthesis, the starting material is usually a short, ribosomally produced peptide that contains a leader (enzyme recognition) and a core peptide. The core peptide is ultimately transformed into the natural product. How RiPP enzymes recognise the leader peptides of their respective substrates has thus far only been poorly understood. Yet this area holds a lot of promise to make RiPP enzymes suitable for industrial applications. Since the leader is essentially a tag that leads to processing, it may be considered a waste product and reducing the size of leader peptides is essential to improve the atom economy of RiPP production. Nguyen et al. provide an important piece of the puzzle by elucidating the 3D structure of an unusually large group of leader peptides, those from proteusin pathways, and demonstrate how this knowledge can be used to dramatically shorten the leader peptide without sacrificing too much enzymatic efficiency: Removing 60 amino acids from an 81 amino acid leader peptide only decreased the yield by 13%.

Overall, this manuscript provides very important information and I recommend publication in NatComms once the manuscript has undergone major revisions. I would like to add that I am not an NMR or MD expert and therefore can only say that these sections appear to be done very well, but refer to an expert for final judgement.

My revision requests are largely related to how the data is presented. NatComms requires articles to be written for a general audience, and that is where this manuscript falls short. In my opinion a general audience would be most attracted to the point made by the authors on p3 L74, and the conclusion. Combined with the additional support the authors provide for the importance of the L(X)4L-motif in RiPP biosynthesis the authors provide important reference points for future RiPP engineering efforts and the exploitation of RiPP enzymes for Trp bromination. I would center the story on those areas.

We thank the reviewer for these comments and directions. The manuscript introduction (lines 83-85) and discussion (lines 83-85) sections have been expanded to bring attention to the improvement in atom economy for production of modified peptides upon rational reduction in length of the vestigial leader peptides.

I know that these comments are, from an authors' perspective, more annoying to deal with than purely scientific comments and the authors are free to ignore them. I apologise if I overstepped, I am only trying to help.

As above, we are deeply appreciative of the reviewer's efforts in making the manuscript better. Thank you!

Specific:

Figure 1a: Not heterocycles in OspA?

*There are indeed no heterocycles installed in OspA, as delineated by Piel in coworkers in the study: Bösch et al, Angew Chem Int Ed **2020** 59, 11763–68 (this paper has been cited in our manuscript). We have noted this in the updated Fig 2a legend.*

Relating to Figure 2e: The Trp appears to sample different orientations and positions, but have a preferred volume it occupies. Is there an way for the authors to relate these data to enzymatic turnover, as in, is one of those likely to be best for processing?

While we appreciate the reviewer's comment and the motivation behind it, unfortunately, at the moment, we lack the experimental data to definitively answer this question. This is due to the fact that we do not have an experimental model of SrpE/MprE-TH1 binding to SrpI. Our in-silico model of the MprE7-TH1/SrpI complex (Fig. S31, Supplementary Movie) does suggest that conformational flexibility of the Trp likely contributes to activity. However, the exact conformation of the Trp residue that leads to a catalytically productive complex formation between the peptide substrate and SrpI is not yet discernable. We hope that the reviewer is sympathetic to this limitation.

I would encourage the authors to run additional biochemical reactions for the data shown in Figures 4c and 5 to reduce the size of the errors. It would also be good to provide calibration curves for at least some of the peptides to ensure that responses in MS are linear in the concentration range of the assay.

All biochemical experiments have now been repeated using fresh preparations of the enzyme and peptide substrates, and data acquired using a high-resolution Agilent 6530C qToF mass spectrometer. Previously, we had acquired data using a lower resolution and lower sensitivity Bruker amaZon ion trap mass spectrometer which conceivably led to higher error bars; the error bars are significantly reduced in the revised manuscript (revised Figures 4c–4f and Figure 5a). During these revision experiments, we now used a higher resolution 2.7 μm particle size HPLC column which better resolved the different peptides (as opposed to a 5 μm particle size column that we had previously used). A calibration curve was generated using the wild type SrpE-TH1 substrate peptide and these data have been added to the revised Supplementary Information file. The description of the methods in the Supplementary Information file has been correspondingly updated. The Excel file with these data is now provided as well.

Figure S16 and accompanying text on page 7, starting line 172: It would be very beneficial for a general audience to expand that section and create a panel or figure to illustrate that point for a general audience.

*We appreciate the reviewer's motivation better orient the audience, and we direct attention to reference 12 in the manuscript (Haft et al, **2010**), which extensively demonstrates the sequence similarity of proteusin leader peptides to nitrile hydratases. In the revised manuscript, in the section mentioned by the reviewer, we have brought forward this reference again, while preserving our structural alignment of the MprE7 leader with that of the nitrile hydratase alpha subunit.*

Could the authors provide NMR evidence that the severely truncated peptide still binds to SrpI with similar affinity and is in fact disordered in solution?

The truncated peptides, when expressed in E. coli, form insoluble inclusion bodies. This is a common finding for small RiPP precursor peptides that are often purified under denaturing conditions. Hence, doing NMR titration experiments with truncated peptides is not feasible. We hope that the reviewer will be sympathetic to this limitation.

For halogenation assays with truncated peptides, we had to append an MBP solubility tag at the N-terminus of the truncated peptides to keep them in solution. Doing NMR titration assays with MBP-tagged truncated peptides is quite onerous as i) signals arising from MBP would overlap with the peptide, and ii) the overall molecular weight of the peptide would increase limiting NMR signal. As before, we hope that the reviewer will be sympathetic to the limitations of the system that we are investigating here.

Reviewer #2 (Remarks to the Author):

In the manuscript under review, the authors investigate the structural characteristics of ribosomally synthesized and post-translationally modified peptides (RiPPs) with a specific focus on proteusin peptides. Employing an array of techniques, including solution NMR spectroscopy, SAXS, and molecular dynamics (MD), they elucidate the three-dimensional structure of proteusin peptides and their interaction with flavin-dependent brominase enzymes.

My expertise predominantly lies in SAXS methodology, so my review will chiefly address the data and conclusions emanating from this technique. Overall, the data are robust, and the analysis appears to be rigorous. However, I have several critical points for consideration:

Figure S7: While the figure successfully negates concerns about radiation damage and structure factor, the SAXS profiles exhibit minor shifts in I(0). This issue, also evident in Figures S8 and S9, likely arises from an instrument scaling error and warrants manual correction, possibly through Primus software.

We appreciate the reviewer for their valuable comments. Figure S7 illustrates SAXS data obtained at three distinct concentrations: 523 µM in red, 486 µM in cyan, and 448 µM in blue. Consequently, these datasets are not anticipated to overlay perfectly. Data at each of the three concentrations were acquired over a 60-minute period, comprising six ten-minute images using an autosampler quartz flow cell. It is important to note that these images were subsequently averaged after confirming the absence of radiation damage. The figure legend has been revised to provide clarity on this detail.

Figure S10: Contrary to the authors' conclusions, the Kratky plots exhibit bell-shaped curves, indicative of structural rigidity rather than flexibility. Neither the P(r) functions nor the small-angle region data sets corroborate the claim of flexibility. I suggest that the authors employ Flory's equation to explore the correlation between Rg and protein length as a more robust analytical method.

We value the reviewer's input and have incorporated these modifications accordingly. The Kratky plots derived from the SAXS data of MprE7-TH1 exhibit semi-Gaussian shapes and not a full Gaussian, reflecting on perhaps some movement in the protein structure. On further analysis, it could be the orientation of the unstructured, flexible 12-residue C-terminal tail. Notably, the plots for the three concentrations (523 µM in red, 486 µM in cyan, and 448 µM in blue) display comparable semi-Gaussian patterns. It's important to note that Flory's equation is more applicable to intrinsically disordered proteins rather than mostly a well-folded structure of MprE7-TH1. The caption for Figure S10 has been adjusted to reflect this clarification.

Line 153: The statement that NMR/WAXSiS and DENSS models offer independent verification is misleading. Both models derive from SAXS data, making their concordance expected rather than confirmatory. Furthermore, the utility of the DENSS model in this context appears questionable.

We thank the reviewer for pointing out this out and the words 'independent structural analyses' has been removed on lines 187-188 (revised line numbering).

Missing Analyses: I could not locate the CRYSOL and OLIGOMER fits of the top-ten NMR models, crucial elements for a comprehensive SAXS analysis. The inclusion of statistical measures such as Chi^2 or CorMap p-values tests for these fits would enhance the study's robustness. Availability of the data in open databases e.g. in SASBDB would be beneficial and strengthen the rigor and trustworthiness of this study. Consider also employing EOM program from ATSAS designed specifically for analyzing flexible/disordered proteins.

The SAXS data have been deposited in the SASBDB site and the SASBDB accession code is:
https://www.sasbdb.org/data/SASDTM5/g6n0zykb40

A project summary can be found here:
https://www.sasbdb.org/project/2180/7kwm7q99ip

The SAXS data have been deposited in the SASBDB site and it is currently under review. A new figure, Figure S15, has been added to the supplementary section. This is a ATSAS Crysol program overlay of the experimental SAXS profile of MprE7-TH1 with the calculated SAXS profile of the best NMR model as refined by the ATSAS Sreflex software. A reasonable Chi-square fit of 2.1 was calculated for this superposition.

Minor Notes:

Line 143: SAXS should not be classified as a spectroscopy technique.

The word spectroscopy has been applied to only CD in the revised manuscript.

Line 241 (SI): The X-ray beam energy should likely read 12 keV rather than 1.2 keV.

Corrections has been made in the revised Supplementary Information file. The energy of the X-ray beam was 8.04 keV (1.5418Angstroms), with the Kratky block attenuation of 22% and a beam diameter of ~100 μm.

In summary, the manuscript is scientifically sound and its contributions are valuable. However, several issues need to be addressed before it is ready for publication.

We thank the reviewer for their review and comments. Changes in the manuscript and the supplementary information have been made, as delineated above.

Reviewer #3 (Remarks to the Author):

This work uses various spectroscopic methods (SS-NMR and SAXS) as well as MD simulation to investigate proteusin peptide-brominase interactions. The proteusin has ordered and disordered regions and it is found that the ordered part can be excised without catalytic function loss. The LXXXXL motif is found to be crucial for Bromination as 1) SrpI interaction features the LXXXXL region and 2) XXXX mutation inhibits Bromination. Modelling is done to suggest the nature of the proteusin peptide-Brominase interaction.

The experimental methodology seems sound. But there are issues of interpretation that should be addressed before publication, in my view.

We very much appreciate the reviewer's constructive comments and valuable suggestions to improve our manuscript.

Some issues to address:

1) The three MD trajectories are strange in that they are so different. This is ascribed to "these observations are suggestive of an equilibrium heterogenous mixture of MprE7-TH1 conformations in solution". An MD trajectory in principle would sample in one trajectory all these states. It seems that what is actually seen are three non-equilibrated trajectories.

It could be worthwhile to look at BB RMSD excluding residues ~60-90. Are those three trajectories similar? It might give confidence that it is the C-terminus that is responsible for trajectory differences.

Note that it is RMSD (versus RMSF by residue) that would point to 'heterogenous mixture' (see following comment).

Towards addressing the reviewer's concerns, we have performed several new MD simulations and data analyses, which have been incorporated into the new Figure 3 of the revised manuscript.

First, we have performed additional MD replicates using the same parameters as defined in the original manuscript. The new replicates are independent trajectories (differential initial velocities) of the production stage of the simulation. The backbone RMSD values of all simulations in the final frame is in the range of 8 to 10 Å when all regions of MprE7-TH1 (N-terminal and C-terminal domains, residues 1–87) are analyzed, suggesting the simulations have properly equilibrated. There are expected fluctuations in the RMSD, but the average value does not increase past the first ~200 nsec of the simulations.

Second, as suggested by the reviewer, we have split the backbone RMSD analysis into the rigid N-terminal domain (residues 1–59) and dynamic C-terminal domain (residues 60–87) to deconvolute their contributions to the overall backbone RMSD. In agreement with the reviewer's predictions, we observe the structured N-terminal domain is highly rigid with consistent backbone RMSD values <5 Å, while the dynamic C-terminal domain shows varying backbone RMSD values between 5–10 Å. These analyses suggest that the conformational heterogeneity of C-terminal domain is the primary contributor to differences observed between overall backbone RMSD calculations for the different trajectories.

We appreciate the reviewer pointing out the important distinction between RMSD and RMSF. We have adjusted the corresponding descriptions in the main text.

2) Continuing in this vein, much of the analysis depends on a conformational selection (versus induced fit) hypothesis: “these observations are suggestive of an equilibrium heterogenous mixture of MprE7-TH1 conformations in solution.” That seems rather circumstantial. It means that there are large barriers in free energy separating conformers. While certainly possible, if the unstructured C-terminal region is genuinely unstructured it is hard to see that it samples distinct conformers. Instead of this hypothesis why isn’t induced fit just as sensible?

We appreciate the reviewer’s comment on this matter and for directing attention to this section of this manuscript. We agree fully that we do not have the data to preclude induced fit, or conformational selection at this point. We have made this point clear in the revised manuscript. Indeed, induced fit mechanism of leader binding to RiPP modifying enzymes has been demonstrated before by Koehnke and Naismith in which a helical region of the RiPP substrate leader changes conformation to form an additional beta strand of the RRE domain’s beta sheet upon binding to a RiPP modifying enzyme (ref. 4 in the manuscript). Firm experimental evidence for conformational selection is still elusive. As such, we do not have the data to exclude either possibility, but we agree with the reviewer that purely conformational selection is not likely to be operative here (and manuscript text has been altered to reflect this).

In the paper that the reviewer highlights below, it is stated that-

“It is well understood that the distinction between the induced fit and the original conformational selection models is not absolute; indeed, an increasing number of cases show that conformational selection is often followed by conformational adjustment.”. We think that such a scenario is operative here which fits the extended conformational selection model that this paper proposes. We do not want to get into the minutiae of this argument in our manuscript because we do not have an experimentally validated model of proteusin leader peptide binding to SrpI which would allow us to more definitely conclude, one way or the other.

3) It is worth citing some refs for conformational selection versus induced fit; e.g. “Induced fit, conformational selection and independent dynamic segments: an extended view of binding events. Csermely, P; Palotai, R and Nussinov, R”, TRENDS IN BIOCHEMICAL SCIENCES 35 (10) , pp.539-546 (2010).

(It has ~600 forward refs). And couching the discussion in these terms.

This reference has been added. We again thank the reviewer for bringing attention to this point.

4) While the results of this study are intriguing, what is missing is insight into the reaction mechanism. What chemistry is being carried out in the Bromination? With the geometrical constraints of the wild-type and mutated-residue complexes available, correlated with the reaction kinetics, it should be possible to suggest a reaction mechanism. To me, this is the raison d’être of this kind of study.

We thank the reviewer for this comment. Manuscript text has been modified to direct the audience towards the extensive literature that now exists which describes the reaction mechanism for flavin-dependent halogenases.

*Reaction mechanisms for flavin-dependent halogenases are well resolved, primarily by the work of Walsh, Naismith, and van Pee. In our earlier study (JACS, **2021**), we had demonstrated that mutation of the SrpI active site residues in line with the mechanistic understanding had the corresponding biochemical effects.*

The involvement of a flavin reductase and the catalase enzymes in the assay further corroborate the assertion that SrpI follows the well accepted reaction mechanism of flavin-dependent halogenases. Four references describing the mechanism of flavin-dependent halogenases have been added to this section, along with two other references that describe the application of two partner enzymes in the in vitro SrpI activity assays.

Typo: “determined for Pro residues are as”

This error has been corrected.

Reviewer #4 (Remarks to the Author):

In their manuscript Nguyen et al. characterize the structural properties of the unusually long bacterial proteusin RiPP leader peptide and its binding to the brominase SrpI. Since the physiological substrate of SrpI, SrpE, is difficult to assess recombinantly, the authors come up with an elegant strategy, by why they fuse the related MprE7 to the tumor homing peptide-1 (TH1), which can be C-terminally brominated in the absence of further post-translational modification. In a detailed NMR study, the authors discover, that a major part of the proteusin leader peptide is structured and they determine its solution structure using NMR spectroscopy and SAXS. They identify that the C-terminal part of the peptide is intrinsically disordered and that this part also presents the essential interaction site with SrpI and characterize SrpI activity as a function of binding site mutations using mass spectrometry. In their docking and MD-derived model of MprE7-TH1 binding to SrpI, the authors explain the importance of the identified LXXXXL peptide for the correct positioning of the C-terminus in the catalytic pocket and compare this model to binding of other known LXXXXL motifs involved in RiPPs.

The study is extensive, using many different techniques and providing new insights into the function of the long proteusin leader peptide. However, several points need to be addressed:

Firstly, this concerns the observation of two sets of NMR peaks at the C-terminal intrinsically disordered peptide of MprE7. One set is populated to 80% in the unbound state. The minor state (populated to 20% when unbound) resembles the bound state, and increases in population as the concentration of SrpI increases. This interesting observation appears in the manuscript far too late and then opens a couple of questions as to how previous data have been analyzed (e.g. secondary structure content, chemical shift perturbations, etc…). Additionally, mentioning the population of the minor state earlier would avoid confusion concerning figures that are cited earlier in the text, for instance the insets in Figure 4. The authors should revisit this critical point and restructure the results section of their manuscript accordingly.

We thank the reviewer for this comment. We have edited the manuscript where we first describe the NMR data to state that two different states were detected, and that we have used the more abundant state (~80%) data for structural assignment.

Secondly, the authors seem to identify that, despite the presence of a folded domain at the N-terminus of the analyzed MprE7 leader, the C-terminal unstructured region is the central interacting region for SrpI and activity measurements convincingly demonstrate that the folded part is dispensable. This seems to *(1)* partially conflict with the last phrase of the abstract that 'RiPP modification enzymes [...] not only [use] small unstructured precursor peptides, but also structured proteins as substrates'. Along these lines and maybe in context with their model of the MprE7-TH1/SrpI complex, *(2)* it would be interesting if the authors could come up with an idea why the folded part exists and what role it might play (or not) in correctly positioning the LXXXXL peptide.

We thank the reviewer for this comment; we will address reviewer's second statement first, and then come back to the first one.

The proteusin substrate peptide interacts with multiple modification enzymes, in addition to the halogenase SrpI. The folded part of the proteusin substrate may or may not be important for interacting with these

additional RiPP biosynthetic enzymes. In RiPP biosynthesis, there is so single universal rule for how substrate leader peptides interact with all the biosynthetic enzymes encoded in a biosynthetic gene cluster, as is delineated in Figure 1 in our manuscript. We think that the proteusin leaders complicate this scenario even further when only specific structured/unstructured parts of the leader specifically interact with a biosynthetic enzyme, such as the SrpI halogenase in this case. In short, we hypothesize that the structured part of the proteusin leader may be important for interaction with other biosynthetic enzymes.

In relation to the first comment, we humbly posit that rather than a conflict, this is a case for altered interpretation. Here, what we are intending to say is that the proteusin leaders have a well-structured domain, just as for globular proteins that have now been shown to be modified just as unstructured RiPP substrates. It is this structural analogy that we are attempting to draw attention to.

Additionally, the following specific points below will need to be addressed:

• The authors state that they assigned 72.5% of the chemical shifts. Can they explain why the remaining resonances were not/could not be assigned?

The reviewer asks a fair question. Incomplete NMR resonance assignment is commonly reported in the literature for ^{1}H detected experiments of IDPs or proteins with IDRs. As an example, authors studying WIP^{C} could achieve no more than 50% backbone assignment using traditional sequential assignment methods {https://pubmed.ncbi.nlm.nih.gov/23870269/}. The primary difficultly associated with NMR peak assignment of these proteins are extensive peak chemical shift overlap, repetitive sequences, signal loss of exposed amide groups due to solvent exchange induced line broadening, and lack of well-defined signal in 3D experiments due to conformational exchange induced line broadening. We experienced several these issues with MprE7-TH1, leading to 72.5% assignment.

Under optimal circumstances, we would attempt to increase the assignment completion through several methods. First, we would perform higher dimensional experiments (4D, 5D) where encoding more into a cross-peak reduces signal overlap {see https://pubmed.ncbi.nlm.nih.gov/26387100/}. Second, we would perform carbon-detected 3D experiments towards enhanced chemical shift dispersion in the direct dimension and optimized exchange processes (see https://pubmed.ncbi.nlm.nih.gov/24333248/). However, the low concentration of MprE7-TH1 (~300 µM) and the instability of the protein precluded us from performing these inherently insensitive experiments.

Despite the incomplete resonance assignment, we believe have sufficient coverage of both the N-terminal and C-terminal domains to support the conclusions of our manuscript.

• Can the authors be more precise (e.g. in the methods section), which data were used in the structure calculation, e.g. a list of constraints that were used, how these were fulfilled etc.

We apologize for not providing this information before. We have now provided the number of dihedral angle, NOE, and RDC restraints used along we any violations. This information has now been included in a new Table in the Supplementary Methods section for structure calculations.

• Which RDCs were used in the structure calculation? Could the authors plot the RDCs from Figure 2d (experimental and predicted) along the protein sequence? Is the quality of agreement similar between the structured regions and the disordered region?

All experimentally determined amide RDCs were used in the structure calculation. A total of 59 $^{1}D_{NH}$ RDC measurements ranging from residues 15–87 were used in the structure calculation. This information has now been included in the Supplementary Methods section for structure calculations.

As requested by the reviewer, we have modified the Figure 2d to explicitly delineate RDCs determined for the N-terminal domain (residues 1–59) and C-terminal domain (residues 60–87). The Q-factor for each fit is similar (in the 0.3 to 0.35 range), which highlights agreement in the quality of the fit between structured and disordered regions.

• The authors should explain briefly what the 'NMR/WAXSiS refined' model is (line 153) also in the main text (not only in the figure legend). Could the authors provide any means to judge the goodness of the fit into the SAXS-derived solvent envelope (Figure 2f)?

The correlation of the final model to the SAXS-derived solvent envelope was estimated using the volume data alignment in the Chimera software and calculated to be around 0.9. Lines 185-187 have been modified accordingly (line numbering per revised manuscript).

• The authors state that, C-terminal from beta2, the remainder of the leader peptide is disordered apart from helix alpha4. Can the authors detail, based on which data they draw this conclusion. In Figure 2c, alpha4 does not seem to be indicated in the SSI (while the RCI-S2 increases around this position). What is the reason for this? Can the authors also indicate (and show) based on which data they conclude that there are 'minimal contacts' (line 167) between alpha4 and the N-terminus of the leader peptide?

We determined that the C-terminal domain is disordered from several lines of corroborating experimental evidence. The CD spectroscopy data reveals a mixture of ordered secondary structure (alpha helical/beta character) and disordered character, in agreement with our experimental structure (Fig. 2b and Fig. S5). In agreement with the CD data, there is a lack of a well-defined secondary structure determined by solution NMR indicated in the SSI derived from chemical shift information (Fig 2c) and the low values (<0.6) in the S^2 model-free order parameter predicted from the RCI (Fig 2c). The MD simulations also point to the C-terminal region as flexible and conformationally heterogenous unlike the well-folded, rigid N-terminal domain (Fig. 3).

We think the signal from the chemical shift derived SSI is too weak to reveal a well-defined helix, but the observation that the RCI-S^2 values increase between residues 70–80 is consistent with a short region of more rigid structure. CS-Rosetta (the chosen method of structure determination) likely modeled an alpha helix at residues 70–80 for two reasons. First, chemical shift guided fragments obtained from the PDB for that section of amino acids are likely to correspond to alpha helical structures. Second, NOE patterns consistent with an alpha helix were observed between residues 73–77, indicating additional evidence of at least a transient alpha 4 helix. Realistically, we believe the alpha 4 helix is a weakly alpha helical structure that samples a range of secondary structures in solution (as determined by visualization of secondary structure changes for the alpha 4 helix across MD simulations – Fig. S18).

No well-defined contacts were observed between N-terminal and C-terminal domain of the leader peptide as revealed by the structure determined based on NOEs and RDCs. For example, there are no observable NOEs between alpha 4 helix and the N-terminal region in amide NOESY NMR experiment (see below figure).

In the revised manuscript, we have highlighted alpha 4 helix NOEs with a new panel in Fig. S18.

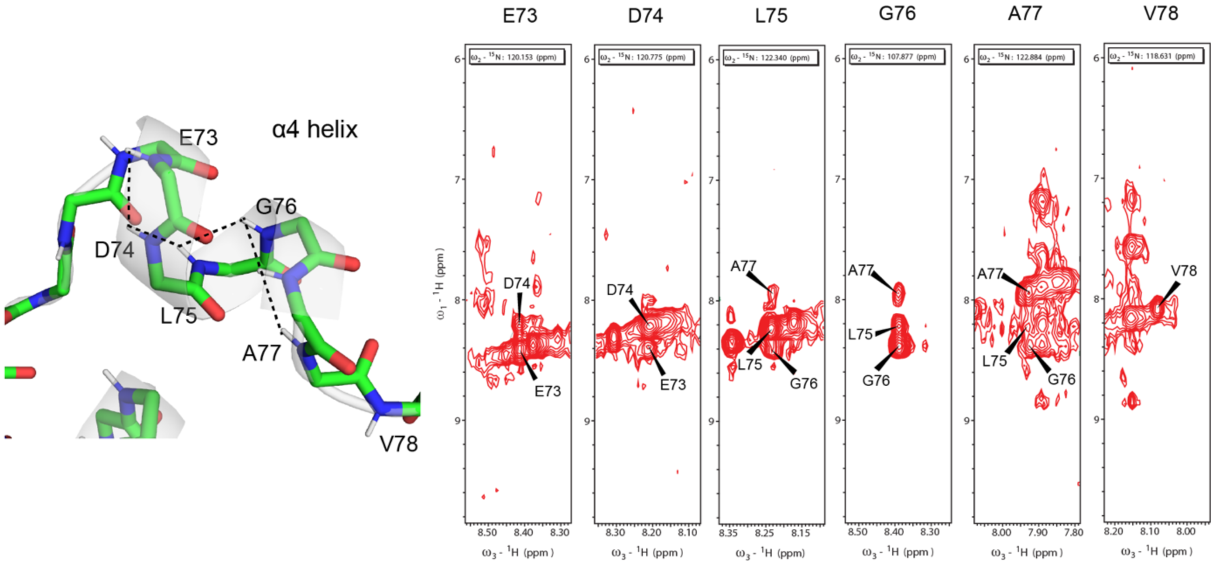

• Figure 3d: Could the authors provide two structures with two different (one long, one short) distances between L75 and T6 and annotate the actual distances onto the structures? This would help appreciate the movement.

We thank the reviewer for this helpful suggestion. We have included the requested analysis in the new Figure 3d of the revised manuscript. We now provide four representative structures of MprE7-TH1 throughout an example MD trajectory (at 0 ns, 8.5 ns, 125 ns, and 500 ns) and explicitly annotate the distances between T6 and L75 on these structures (distances ranging from 10.5 Å to 39.2 Å). We hope this provides a better picture of the large variations in conformations for the C-terminal domain observed throughput the simulations.

• In lines 207-214, the authors discuss NMR line broadening of MprE7-TH1 spectra in complex with SrpI. They first suggest 'intermediate exchange NMR line broadening', then that the line broadening is 'likely due to rapid transverse spin relaxation in the ~60 kDa […] complex.' As no data on the exchange behavior of the complex exists, the authors should be more open in their interpretation, speak about line broadening in a more general sense and only then discuss the different possibilities why this line broadening may occur.

To tone down this statement, we have modified the line as follows: "The observed NMR line broadening of MprE7-TH1 amide N-H signals could be due to rapid transverse spin relaxation in the ~60 kDa MprE7-TH1/SrpI complex, or other changes in the exchange behavior of MprE7-TH1 in the SrpI complex."

• Figure S18 shows an intensity ration between the spectra of MprE7-TH1 in complex with SrpI and alone, whereby no region seems to show specific line broadening, as the authors also mention in the main text. Can the authors state at which concentrations these intensity ratios were acquired and, whether they used the bound or free state peaks for calculating the intensity ratios? The label of the x-axis should be bigger.

We apologize for omitting this information. Peak intensity ratio analysis (I/I_0) of SrpI bound MprE7-TH1 relative to unbound MprE7-TH1 was performed at the 1:1 molar ratio of MprE7-TH1/SrpI titration point. At this titration point, MprE7-TH1 and SrpI are both at 42.1 µM. For peaks displaying one-state behavior in unbound MprE7-TH1, I/I_0 was determined by comparing intensity of "state A" NMR peaks in the unbound MprE7-TH1 versus SrpI bound MprE7-TH1. For peaks displaying two-state behavior in unbound MprE7-TH1, I/I_0 was determined by comparing intensity of "state A" NMR peaks in the unbound MprE7-TH1 versus "state B" (bound state) in the SrpI bound MprE7-TH1.

We have included this information in the Methods as well as the legend of Fig. S19.

In addition, as requested by the reviewer, the X-axis labels for Fig. S19 have increased in font size.

• The authors observe a minor state in the C-terminal disordered part of the leader sequence, which gets more populated as the concentration of SrpI increases. In view of this observation: Which peaks were used to generate the CSPs and intensity ratios (see also previous point)? Which peaks were used to calculate the SSI and RCI-S2 and what would using the respective other peak yield? Judging from the assignment strip plot, the carbon chemical shifts of the bound and the unbound states are the same. However, it would be interesting to know if this is indeed true. The chemical shift of the carbon dimension is not annotated on the plots (Figures S11-S14)

For CSP analysis, chemical shifts of "state A" NMR peaks in the unbound MprE7-TH1 were compared to the chemical shifts of "state B" NMR peaks in the SrpI bound states of MprE7-TH1. The intensity ratios were demined as outlined in the previous response to the reviewer (see above). "State A" NMR peaks in the unbound MprE7-TH1 were used by TALOS-N to determine SSI and RCI-S2 predictions, and towards structure calculation. We have now included all this information in the updated Methods section.

As noted by the reviewer, the CA, CB, and CO chemical shifts of "state B" are very similar to those of "state A". Here's an example comparing strips from the HNCACB for W87 "state A" vs W87 "state B", highlighting similarity in the CB chemical shift values between the two states.

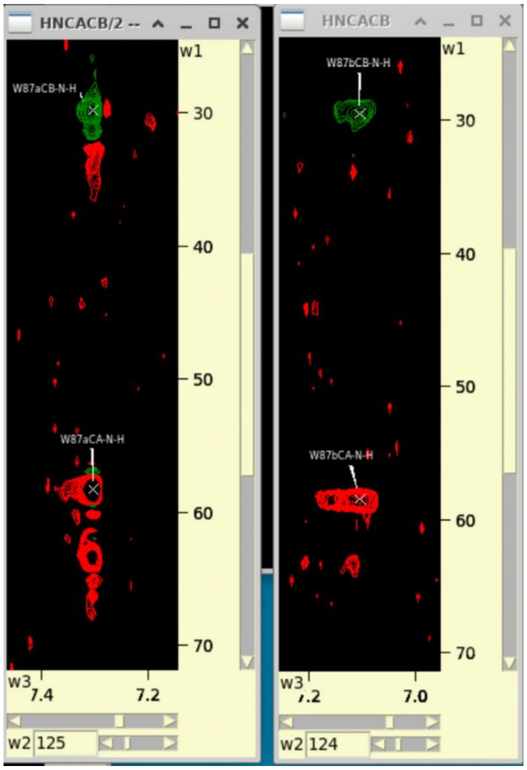

Thus, while the amide chemical shifts for "state A" and "state B" are different, we would expect TALOS-N derived SSI and RCI-S2 for the two states to be similar based on CA, CB, and CO chemical shifts.

• Based on previous data on OspA, the authors speculate that the N-terminal folded region might be important for the overall conformation of the proteusin peptide (and thus the disordered C-terminus). This could easily be tested for MprE7-TH1 using only the C-terminal disordered region. Would the authors be able to provide these data?

We thank the reviewer for this comment, but this experiment is technically not feasible to perform. As we discuss with Reviewer 1 as well, the truncated C-terminal leader peptide is insoluble when expressed in E. coli. It can only be purified as an MBP-fusion protein, or under denaturing conditions. In either case, performing NMR, or even CD spectroscopy experiments is not feasible and/or will not yield meaningful structural data. We hope that the reviewer will be sympathetic to this limitation. The insolubility of RiPP substrate peptides is an often-encountered problem in the field.

Minor points:

• Figure 1e: Why is the leader peptide brown and not blue as in the other sub-figures? This should be changed or explained in the legend.

The leader peptide in Figure 1e has been colored blue to be consistent with leader peptides in other Figure 1 panels.

Somehow, Figure 1e does not seem to fit to the other sub-figures, since it describes a reaction, not a mechanism and indeed, the authors state that SrpI follows the model from Figure 1c. Maybe the authors could find a way to set Figure 1e apart from the rest of the figure.

The authors appreciate the reviewer's motivation to make the manuscript easier to read; thank you! We have boxed the panel e in Figure 1 and added text to the figure to explicitly mention that panel e denotes the reaction catalyzed by SrpI.

• Lines 85 and 86: 'in the SrpE leader than has been…' should be changed to '...that has been…' and 'withs' to 'with'. The sentence also seems to be lacking a citation.

These errors have been corrected, and a citation has been added.

• Lines 94/95: 'Gratifyingly, leader sequences from other proteusin peptides, MprE7 and OspA, supported SrpI activity (Fig. 2a).' The reference to Figure 2a seems somewhat misplaced, since it shows sequence alignments rather than SrpI activity.

Reference to Figure 2a has been deleted from this sentence in the revised manuscript.

• Line 95: 'Of these, gene…', please insert 'a': 'Of these, a gene…'

This edit has been incorporated in the revised manuscript.

• Figure S5 would benefit from a more detailed legend. What are the different secondary structure elements that are considered in the fit? Which are beta-sheets, which are disordered for example, as indicated in the main text (lines 99/100)?

We have expanded the Fig. S5 legend with the following text:
The fit is from a "single spectrum analysis", used to distinguish different types of secondary structure elements from CD spectroscopy curves. From the BeStSel website: "The secondary structure basis components of BeStSel are derived from DSSP. Parallel and antiparallel beta-sheets are distinguished, and antiparallel beta-sheets are divided into three subgroups: left-hand twisted, relaxed, and right-hand twisted (anti1, anti2, anti3, respectively). The regular part of helices (helix1) and the distorted ends (helix2) are separated, similarly to SELCON3, however, only α-helices are counted. BeStSel sorts 310-helix to "others". The definition of turn is identical to that in DSSP. The figure shows the eight basis components of BeStSel in relation to DSSP." The pie chart in the top right of the figure shows the % of different secondary structure elements of MprE7-TH1 as determined by BeStSel from the MprE7-TH1 CD spectroscopy curve.

• Figure S8: There seem to be points in a different red/cyan/blue under the circles with a darker border. It does not become clear from the figure legend what this might be (raw data and Guinier fit?). The authors should clarify this and maybe choose a line or so for fitting (something that is distinct from the raw data).

We have added three panels for the Guinear fit analysis one for each concentration to clarify. As rightly suggested, the raw data is now represented by circles and the Guinear fit with a solid line.

• U-[15N/13C] should be explained.

U-[$^{15}N/^{13}C$] means uniformly ^{15}N- and ^{13}C-labeled protein. We now provide this definition in the main text.

• A citation for the random coil index seems to be missing (line 147).

*We thank the reviewer for pointing out this missing important reference (Berjanskii & Wishart JACS **2005** 127(43) 14970–14971). We have included this reference in the revised manuscript.*

• Figure 2e: It would be helpful to have a color legend for this figure.

Legend for Figure 2e has been expanded to include an explanation for the colors (blue to red from the N- to the C-terminus).

• Figure 4: The authors should explain how they analyzed the time dependent SrpI activity in sub-figures c to f briefly in the figure legend (especially as the rest of the figure is focused on NMR experiments); a few language errors need to be corrected in the figure legend.

The Figure 4 legend has been expanded to include these details. The Supplementary Information document has also been expanded to provide further details for data analysis.

• A legend for the movie seems to be missing.

We thank the reviewer pointing out the missing legend. The Supplementary Information document now includes a legend for the movie.

REVIEWER COMMENTS

Reviewer #1 (Remarks to the Author):

I would like to thank the authors for their very thorough revision of this already strong manuscript. I have no additional requests and look forward to seeing the manuscript published.

Reviewer #2 (Remarks to the Author):

I would like to express my appreciation to the authors for diligently addressing the concerns highlighted in my previous review. The manuscript has been significantly improved with the necessary corrections, and I believe it is now in a suitable state for publication. However, I would like to offer two minor suggestions for consideration, which are intended as recommendations rather than mandatory revisions:

1) In the Supplementary Information section, specifically regarding Small-angle X-ray scattering (SAXS) data acquisition and processing, the authors mention a wavelength of 1.54 Å and an X-ray energy of 8.04 keV. As these two parameters essentially convey the same information, I recommend considering the removal of one to streamline the text.

2) Additionally, it could be beneficial for the authors to consider uploading their P(r) functions, models and fits to the Small-Angle Scattering Biological Data Bank (SASBDB), alongside the raw experimental data. This would not only enhance the robustness of the presented data but also serve as a valuable resource for future readers and researchers in the field.
Please also consider adding an entry ID code or the full link to the main text of the article.

These suggestions are intended to further refine the paper and are not prerequisites for its acceptance.

Reviewer #3 (Remarks to the Author):

The authors have done a good job of addressing my concerns.
I have no objections to publication now.

Reviewer #4 (Remarks to the Author):

In their revised manuscript, Nguyen et al. replied to all my comments and provide a clear and comprehensive description of the long proteusin leader peptide, its structure, binding to SrpI and SrpI activity. Only few comments remain to be addressed.

(1) While the parts of the manuscript dealing with the unbound proteusin peptide now clearly describe the two states observed in the spectrum to 80% and 20%, respectively, the paragraph describing the interaction with SrpI (lines 247 to 254) has not been adapted to the fact that states A and B are now already described earlier in the manuscript. Only minor changes in the text are necessary to take this into account and should be made.
This also affects the analysis of the corresponding figures, which should be adapted:

• Fig. S19: I would encourage the authors to use the same set of peaks for the analysis of intensity ratios, i.e. state A in the bound form versus state A in the unbound form. And then, maybe as a second panel state B in the bound form versus state B in the unbound form (where the intensity should increase). The interaction site should be quite clearly delineated with this analysis.
• Fig. 4: The supplementary methods suggest that state A chemical shifts are compared to those of state state B. This is currently not expressed in the figure and should be taken over into the figure legend. As slow exchange already exists in the unbound form, the chemical shift difference between state A and state B will also exist in the unbound form. The most telling analysis in this case would be the difference in population between the two states in the unbound and bound experiment (as in Fig. S20), rather than the chemical shift difference.

(2) In the reply to the reviewers' comments, the authors explain that the alpha helix in the C-terminal tail is probably only populated transiently. This explanation seems logical and should also be emphasized in the manuscript.

(3) Citations for tools used in the manuscript seem to be partially missing, e.g. SELCON3 (Fig. S5), DSSP (Fig. S5), Bax DC server, DENSS, CRYSOL, OLIGOMER (Fig. 2)

(4) Fig. 4: The stretch 38-C leads to an even lower SrpI activity in this new data set than 67-C. Can the authors explain this? In this context, is the statement that the proline region might be important for the interaction still relevant?

Other points:

• line 156: I suggest to use 'most intense' rather than 'most abundant'
• line 240: please delete 'intermediate exchange' to be more neutral
• line 246: I suggest to use 'line broadening' rather than 'intermediate exchange behavior'

We thank the reviewers for their thoughtful comments and critique, addressing which has undoubtedly made the manuscript stronger and more accessible to a general audience. Reviewers 1 and 3 did not recommend further changes. Hence, we reproduce the comments from Reviewers 2 and 4, who had suggested further improvements, verbatim below and provide a point-by-point response in *dedicated font*.

Reviewer #2 (Remarks to the Author):

I would like to express my appreciation to the authors for diligently addressing the concerns highlighted in my previous review. The manuscript has been significantly improved with the necessary corrections, and I believe it is now in a suitable state for publication. However, I would like to offer two minor suggestions for consideration, which are intended as recommendations rather than mandatory revisions:

1) In the Supplementary Information section, specifically regarding Small-angle X-ray scattering (SAXS) data acquisition and processing, the authors mention a wavelength of 1.54 Å and an X-ray energy of 8.04 keV. As these two parameters essentially convey the same information, I recommend considering the removal of one to streamline the text.

The authors concur that the wavelength and X-ray energy values are redundant. Consequently, we have made the required adjustments and eliminated the segment 'X-ray energy of 8.04keV' in line 289 of the supplement.

2) Additionally, it could be beneficial for the authors to consider uploading their P(r) functions, models and fits to the Small-Angle Scattering Biological Data Bank (SASBDB), alongside the raw experimental data. This would not only enhance the robustness of the presented data but also serve as a valuable resource for future readers and researchers in the field.

Please also consider adding an entry ID code or the full link to the main text of the article.

The SAXS structure of proteusin peptide has been submitted with the SASBDB code, 'SASDTM5'. Presently, the entry is on hold and accessible only to individuals with URLs containing a key string following the SASBDB accession code (link below). The code and link have now been incorporated into the main text at line 420.
https://www.sasbdb.org/data/SASDTM5/g6n0zykb40

These suggestions are intended to further refine the paper and are not prerequisites for its acceptance.

Reviewer #4 (Remarks to the Author):

In their revised manuscript, Nguyen et al. replied to all my comments and provide a clear and comprehensive description of the long proteusin leader peptide, its structure, binding to SrpI and SrpI activity. Only few comments remain to be addressed.

(1) While the parts of the manuscript dealing with the unbound proteusin peptide now clearly describe the two states observed in the spectrum to 80% and 20%, respectively, the paragraph describing the interaction with SrpI (lines 247 to 254) has not been adapted to the fact that states A and B are now already described earlier in the manuscript. Only minor changes in the text are necessary to take this into account and should be made.

We appreciate the reviewer pointing this out. We have rectified this the revised main text.

This also affects the analysis of the corresponding figures, which should be adapted:

• Fig. S19: I would encourage the authors to use the same set of peaks for the analysis of intensity ratios, i.e. state A in the bound form versus state A in the unbound form. And then, maybe as a second panel state B in the bound form versus state B in the unbound form (where the intensity should increase). The interaction site should be quite clearly delineated with this analysis.

As suggested by the reviewer, we have modified Fig. S19 to include the following new analysis:

"state A" in SrpI bound MprE7 vs "state A" of free MprE7

"state B" of SrpI bound MprE7 vs "state B" of free MprE7

"state B" of SrpI bound MprE7 vs "state A" of free MprE7

As suggested by the reviewer, this analysis delineates the SrpI binding site to the C-terminal region of MprE7.

The corresponding figure legends and method sections have also been updated.

• Fig. 4: The supplementary methods suggest that state A chemical shifts are compared to those of state state B. This is currently not expressed in the figure and should be taken over into the figure legend.

This information has now been incorporated into the Fig 4 legend.

As slow exchange already exists in the unbound form, the chemical shift difference between state A and state B will also exist in the unbound form. The most telling analysis in this case would be the difference in population between the two states in the unbound and bound experiment (as in Fig. S20), rather than the chemical shift difference.

We have modified the corresponding text to put more emphasis on this finding. We also modified Fig. S20 to include the new "state A" nomenclature used throughput the text. The methods have also been modified to include how the comparison was performed.

(2) In the reply to the reviewers' comments, the authors explain that the alpha helix in the C-terminal tail is probably only populated transiently. This explanation seems logical and should also be emphasized in the manuscript.

As suggested by the reviewer, we have incorporated the idea of the transiently populated a4 helix into the revised manuscript on lines 213-218.

(3) Citations for tools used in the manuscript seem to be partially missing, e.g. SELCON3 (Fig. S5), DSSP (Fig. S5), Bax DC server, DENSS, CRYSOL, OLIGOMER (Fig. 2)

The following citations have been added to the revised manuscript:

SELCON3 (Fig. S5)
Estimation of protein secondary structure from circular dichroism spectra: inclusion of denatured proteins with native proteins in the analysis
https://pubmed.ncbi.nlm.nih.gov/11112270/

DSSP (Fig. S5)
Dictionary of protein secondary structure: pattern recognition of hydrogen-bonded and geometrical features
https://pubmed.ncbi.nlm.nih.gov/6667333/

Bax DC server
There's no citation for this only the linker to the server; the server linked has been provided
https://spin.niddk.nih.gov/bax/nmrserver/dc/

DENSS
Ab initio electron density determination directly from solution scattering data
https://www.nature.com/articles/nmeth.4581

CRYSOL
CRYSOL - a Program to Evaluate X-ray Solution Scattering of Biological Macromolecules from Atomic Coordinates
https://onlinelibrary.wiley.com/doi/abs/10.1107/S0021889895007047

OLIGOMER
PRIMUS: a Windows PC-based system for small-angle scattering data analysis
https://onlinelibrary.wiley.com/iucr/doi/10.1107/S0021889803012779

(4) Fig. 4: The stretch 38-C leads to an even lower SrpI activity in this new data set than 67-C. Can the authors explain this? In this context, is the statement that the proline region might be important for the interaction still relevant?

The 38–C truncated substrates does indeed show lesser conversion than the 67–C truncated substrate, as the reviewer points out, but the differences are not large (80% conversion vs 85% conversion, respectively). We are of the opinion that for a slow enzyme such as SrpI, these conversions are comparable. The take home point here is that the truncated peptides are still competent substrates.

We do think that the polyproline region might be important (though we lack the NMR CSP to show this). We think so because the 61–C truncation variant is a marginally better substrate than the 67–C variant (though, as before, the differences are not large, and the point remains that the truncation variants are competent substrates).

Other points:

• line 156: I suggest to use 'most intense' rather than 'most abundant'
We changed this as directed.

• line 240: please delete 'intermediate exchange' to be more neutral

This change has been made.

• line 246: I suggest to use 'line broadening' rather than 'intermediate exchange behavior'

We changed all instances of intermediate exchange to use "line broadening" instead.
